# Enhancing self-care education amongst medical students: a systematic scoping review

**DOI:** 10.1186/s12909-023-04965-z

**Published:** 2024-01-08

**Authors:** Darius Wei Jun Wan, Laura Shih Hui Goh, Mac Yu Kai Teo, Celestine Jia Ling Loh, Gerald Hng Kai Yak, Joanna Jing Hui Lee, Nila Ravindran, Nur Diana Abdul Rahman, Min Chiam, Eng Koon Ong, Nagavalli Somasundaram, Ying Yin Lim, Gillian Li Gek Phua, Lalit Kumar Radha Krishna

**Affiliations:** 1 Division of Palliative and Supportive Care, National Cancer Centre Singapore, 30 Hospital Boulevard, Singapore, 168583 Singapore; 2 Yong Loo Lin School of Medicine, National University Singapore, Singapore, 1E Kent Ridge Road NUHS Tower Block Level 11, Singapore, 119228 Singapore; 3 Obstetrics and Gynaecology, KK Women’s and Children’s Hospital (KKH), 100 Bukit Timah Road, Singapore, 229899 Singapore; 4 Duke-NUS Medical School, 8 College Road, Singapore, 169857 Singapore; 5 Division of Cancer Education, National Cancer Centre Singapore, 30 Hospital Boulevard, Singapore, 168583 Singapore; 6 Division of Medical Oncology, National Cancer Centre Singapore, 30 Hospital Boulevard, Singapore, 168583 Singapore; 7 Division of Palliative Care, Alexandra Hospital, 378 Alexandra Rd, Singapore, 159964 Singapore; 8 Lien Centre for Palliative Care, Duke-NUS Medical School, National University of Singapore, 8 College Rd, Singapore, 169857 Singapore; 9 Palliative Care Institute Liverpool, Academic Palliative & End of Life Care Centre, Cancer Research Centre, University of Liverpool, 200 London Road L3 9TA, Liverpool, UK; 10 Centre for Biomedical Ethics, National University of Singapore, Blk MD11, 10 Medical Drive, #02-03, Singapore, 117597 Singapore; 11 PalC, The Palliative Care Centre for Excellence in Research and Education, Singapore, PalC c/o Dover Park Hospice, 10 Jalan Tan Tock Seng, Singapore, 308436 Singapore; 12 Assisi Hospice, 823 Thomson Road, Singapore, 574627 Singapore

**Keywords:** Self-care, Medicine, Medical education, Medical students, Palliative care

## Abstract

**Background:**

Reports of emotional, existential and moral distress amongst medical students witnessing death and suffering of patients during their clinical postings have raised awareness on the need for better psycho-emotional support during medical school. Furthermore, the stress experienced by medical students stemming from the rigours of their academic curriculum underlines the need for greater awareness on mental health issues and better self-care practices across medical training. With such programmes lacking in most medical schools, we propose a systematic scoping review (SSR) to map and address our research question, “what is known about self-care education interventions amongst medical students?”.

**Methods:**

We adopted the Systematic Evidence-Based Approach to guide a systematic scoping review (SSR in SEBA) of relevant articles published between 1st January 2000 and 30th June 2023 in PubMed, Embase, PsycINFO, ERIC, Google Scholar, and Scopus databases. The included articles were independently and concurrently thematically and content analysed, with complementary categories and themes combined using the Jigsaw Approach. The domains created from the Funnelling Process framed the discussion.

**Results:**

A total of 6128 abstracts were identified, 429 full-text articles evaluated, and 147 articles included. The 6 domains identified were definition, topics, pedagogy, influences, outcomes and assessment. Most interventions were promising, though peer-led mindfulness-based interventions showed most promise in enhancing engagement, positively impacting personal wellbeing, and improving patient care. Overall, however, self-care education was poorly recognized, adopted and integrated into curricula.

**Conclusion:**

Greater dedicated time and conducive practice environments within medical school curricula is required to enhance medical student wellbeing. Host organizations must ensure faculty are appropriately selected to instil the importance of self-care, be trained to assess and personalize self-care interventions and provide longitudinal assessment and support. Further study into assessing self-care capabilities is required.

**Supplementary Information:**

The online version contains supplementary material available at 10.1186/s12909-023-04965-z.

## Background

Recent reviews into how medical students cope with caring for the dying and attending to patient deaths have raised questions into how medical schools support students across their training trajectories [1]. Pivotally, reports reveal that rising anxiety, distress and compromises to mental and general wellbeing amongst medical student predispose them to medical errors, and jeopardize patient communication and care [2–8]. This does not only underscore the need to review current curricula and support services, but also the need to improve and innovate education into self-care. However, self-care education escapes the focus of most medical school curricula and remains rudimentary.

The call for robust self-care education is further underlined by increasing evidence that medical students require individualized approaches to cope with their competing academic, research, clinical, administrative, social, relational, familial and individual commitments and existential and ethical dilemmas [9–13]. Here, empowering medical students to devise their own means of supporting themselves is key.

Acknowledging these gaps in the medical curricula, we undertook a review to map self-care education amongst medical students guided by our primary research question, *“What is known about self-care education interventions amongst medical students?*”. For the purposes of this review, self-care education is characterized as “*a spectrum of knowledge, skills and attitudes including self-reflection and self-awareness in identifying and preventing burnout with professional boundaries and handling grief and bereavement appropriately”* (p. 77) [8].

## Methods

**Fig. 1:**
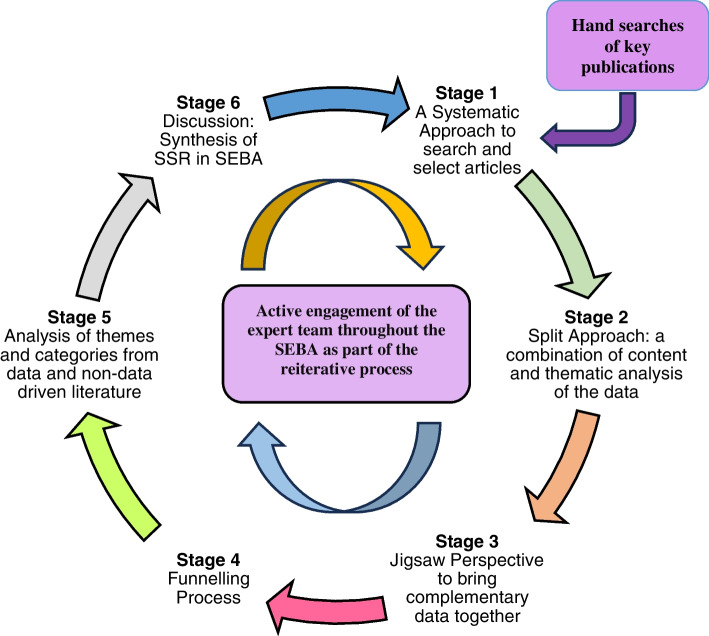
The SSR in SEBA process [23]

A Systematic Evidenced Based Approach guided systematic scoping review (henceforth SSR in SEBA) was adopted to map prevailing literature on self-care education amongst medical students [14–17]. This SSR in SEBA was overseen by an expert team comprising of medical librarians from the Yong Loo Lin School of Medicine (YLLSoM), and local educational experts and clinicians at NCCS, the Palliative Care Institute Liverpool, YLLSoM and Duke-NUS Medical School who guided, oversaw and supported all stages of SEBA to enhance the reproducibility and accountability of the process [14, 15, 17, 18] (Fig. 1). This SSR in SEBA is also shaped by SEBA’s constructivist ontological perspective and relativist lens, as well as the principles of interpretivist analysis to enhance reflexivity of the research analysis and discussions [19–22].

### Stage 1 of SEBA: systematic approach

i. Determining the title and research question and inclusion criteria

The PICOs format and the PRISMA-P 2015 checklist (see Additional file 1) were employed to guide the primary research question, *“What is known about self-care education interventions amongst medical students?”.* The secondary research questions were, “*How are self-care education interventions structured in medical school curriculum?, “What topics are included in self-care education curriculum in medical schools?”, “How is self-care in medical schools assessed?”* and *“What factors support and hinder self-care education interventions in medical schools?”* (Table 1).

**Table 1:** PICOs, inclusion criteria and exclusion criteria applied to database search

	Inclusion Criteria	Exclusion Criteria
Population	Undergraduate and postgraduate medical students	Medical students undergoing a gap year or leave of absence. Doctors Allied health specialties such as dietetics, nursing, psychology, chiropractic, midwifery, social work Non-medical specialties such as clinical and translational science, veterinary, dentistry
Intervention	Self-care education in medical school Including: method of delivery, administrative considerations, barriers and facilitators, content taught, and assessment methods to ascertain success.	Interventions that do not involve self-care education in medical schools and/or for medical students
Comparison	Comparison of the different efficacy of self-care intervention programs in medical schools Comparison of the different methods of measuring self-care efficacy intervention programs in medical school	
Outcome	Impact of self-care education on student’s well-being, mental health, welfare, and professional identity formation	
Study design	Qualitative, quantitative, and mixed study methods Systematic review, literature reviews, and narrative reviews, grey literature. Year: 1st January 2000 – 30st June 2023

ii. Searching

Searches were conducted on PubMed, Embase, PsycINFO, ERIC, Google Scholar and Scopus databases and key medical education journals, including BMC Medical Education, Academic Medicine, Medical Education, Medical Teacher, Medical Education Online and Canadian Medical Education Journal published between 1st January 2000 and 30th June 2023. It was conducted independently by authors DWWJ, LSHG, GLGP, CKRL, JAL, NAH, OEK, NS and LKRK. Variations of the terms “self-care education”, “medical students” and “medical education” were applied. This timeframe was selected to facilitate a viable and sustainable research process and to account for prevailing manpower and time constraints [23]. These searches were also accompanied by ‘snowballing’ of the references of included articles to ensure a more comprehensive review [24]. The full search strategy can be found in Additional file 2*.*

Each of the nine members of the research team consolidated their own lists of articles to be included. To reach an agreement on the final set of articles to be reviewed, the team then adopted Sandelowski and Barroso [25]‘s *‘negotiated consensual validation’* that saw *“research team members articulate, defend, and persuade others of the ‘cogency’ or ‘incisiveness’ of their points of view*”. The accepted list of articles was then consolidated into a master list for further sieving to determine their suitability.

iii. Extracting and charting

The titles and abstracts were subsequently independently reviewed by GLGP, LYY, DWWJ, LSHG, CKRL, JAL, NAH, OEK, NS and LKRK using an abstract screening tool. The team then discussed their findings for the deconflicting process, similarly applying ‘*negotiated consensual validation’* to finalize the list of articles to be included [25]. This process involved the screening of the abstracts and titles of the articles, followed by a deeper in-depth sieve of the full text of each article. Articles that did not fit in the inclusion criteria in any of these two stages were removed whilst articles that met the inclusion criteria proceeded to the data extraction and quality assessment stages.

iv. Assessing quality of articles

NDAR, MC, DWWJ, LSHG, GHKY, JJHL and CJL individually appraised the quality of the quantitative and qualitative studies using the Medical Education Research Study Quality Instrument (MERSQI) [26] and Consolidated Criteria for Reporting Qualitative Studies (COREQ) [27] (see Additional file 3).

### Stage 2 of SEBA: split approach

- Summary and tabulation of full-text articles

The Split Approach [28] was carried out by three teams. The first team (LSHG, MYKT, NR, CJLG, NS, YLL) summarized and tabulated the included full-text articles in keeping with recommendations drawn from Wong, Greenhalgh [29]‘s RAMESES publication standards and Popay, Roberts [30]‘s “*Guidance on the conduct of narrative synthesis in systematic reviews*”. A tabulated summary of the included articles is enclosed in Additional file 3.

b. Braun and Clarke’s thematic analysis
  Guided by Braun and Clarke [31]‘s approach to thematic analysis, the second team of researchers (DWJW, GHKY, GLGP, OEK, LKR) independently reviewed the included articles to extract relevant findings. They subsequently crafted a code book from the extracted data categorized according to the emerging themes. In an iterative step-by-step analysis process [32], the team combined each new emerging code with previous codes. This formed fresh themes that were derived from the raw data with no prior classification [33]. Thereafter, the team organized meetings to discuss their independent findings, shortlisting the final list of themes through ‘negotiated consensual validation’ [25].
c. Hsieh and Shannon’s directed content analysis

Concurrently, the third team of researchers (NR, JJHL, MC, NDAR) employed Hsieh and Shannon [34]‘s approach to directed content analysis. This entailed the identification and operationalizing of a priori *coding categories* [34–39]. Here, codes and categories were drawn from Drolet and Rodger’s study entitled, “*A Comprehensive Medical Student Wellness Program—Design and Implementation at Vanderbilt School of Medicine*” [40]. Known as the ‘coding agenda’ [41, 42], the research team adopted these codes as a template for coding the included articles. This served to diminish concerns on the inconsistency, incoherence and omission of negative results seen in thematic analysis [18, 43–50]. The team also prescribed new codes to any data uncaptured by the priori codes [41]. ‘Negotiated consensual validation’ was similarly practiced by the team to attain consensus on the final categories [25, 37].

### Stage 3 of SEBA: jigsaw perspective

The Jigsaw Perspective employed Phases 4 to 6 of France et al. [51]‘s adaptation of Noblit et al. [52]‘s seven phases of meta-ethnographic approach. This stage entailed DWJW, LSHG, MYKT, CJLL, GHKY, NR, OEK, GLGP, NS and LKRK contrasting themes and subthemes with the categories and subcategories identified. Upon verifying the similarities by comparing the codes contained within each group of data, the researchers then merged complementary categories and themes, as well as complementary subthemes and subcategories, to form larger ‘themes/categories’.

### Stage 4 of SEBA: funnelling

DWJW, LSHG, MYKT, CJLL, GHKY, NR, OEK, GLGP, NS and LKRK compared the ‘themes/categories’ with the tabulated summaries [51, 52] and included quality appraisals using MERSQI and COREQ [26, 27]. This led to domains that formed the basis of the discussion’s ‘line of argument’ in Stage 5 of SEBA.

**Fig. 2:**
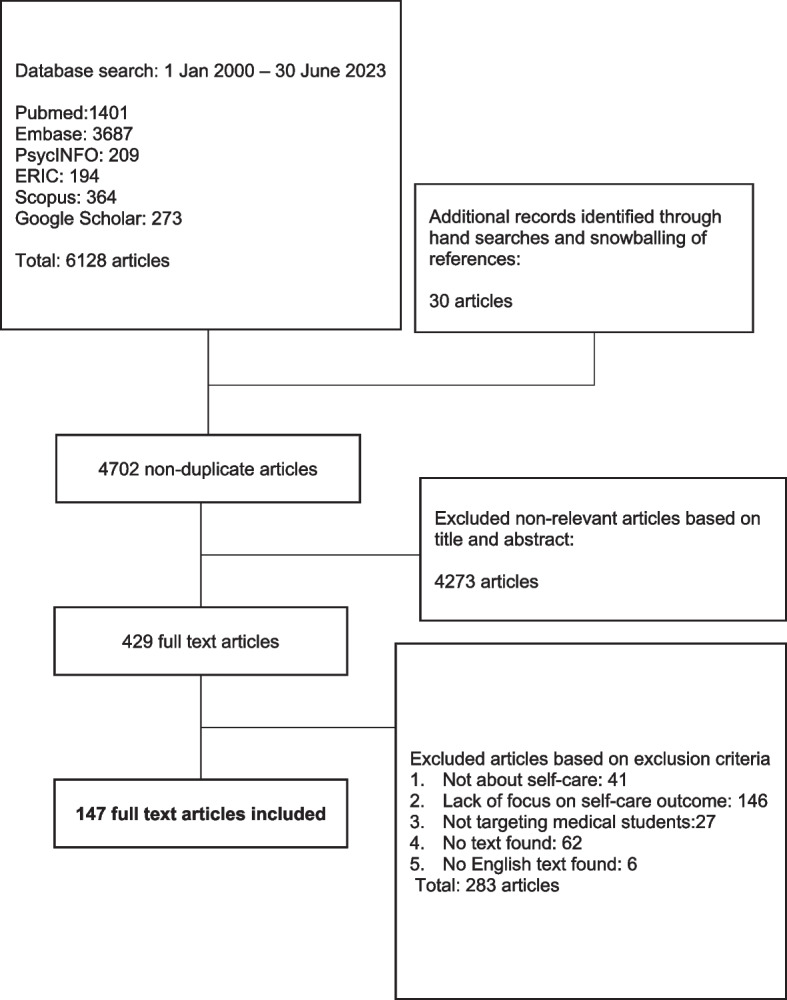
PRISMA flowchart

A total of 6128 abstracts were reviewed, 429 full text articles were evaluated, and 147 articles were included (Fig. 2).

## Results

The Funnelling process revealed six domains: definition, topics, pedagogy, influencers, outcomes and assessments. Many of these domains were merely listed in the included articles without any accompanying descriptions or clarifications. Thus, to enhance clarity and facilitate the review, we have summarized and presented the domains in tables.

### Domain 1: conceptualization of self-care education

Self-care education in medical schools can be characterized as interventions that seek to promote positive coping strategies and reflective practice to boost psychological, emotional, and physical well-being [5, 53–56] whilst fostering competent, caring, and resilient physicians [57]. This ‘catch-all’ characterization allows the inclusion of an expanding array of interventions and acknowledges the notion that a variety of options are being tried, adapted, or used on their own or in combination to meet the needs, goals, individual preferences, working styles, experience, attitudes and skills of medical students. This wide conceptualization of self-care education accounts for a mix of options to cater to a medical student’s individual historical, socio-cultural and contextual narratives, as well as psycho-emotional states, in different settings, stages of training, specialities, cultures and curricula.

### Domain 2: topics and intervention

A wide range of interventions are used to introduce self-care. Table 2 details the various contents espoused within the included publications for ease of review.

**Table 2:** Topics and interventions

Name of intervention	Content of intervention	References
Mindfulness	1. Mindful breathing 2. Mindful meditation 3. Mindful awareness 4. Mindful eating 5. Managing reactivity 6. Reflective listening 7. Mapping out feelings 8. Journaling 9. Mindful yoga 10. Grateful thinking 11. Muscle relaxation 12. Staying present 13. Understanding stress 14. Informal mindfulness practices 15. Components of cognitive behavioural therapy 16. Importance and influence of families 17. Developing skills in mindfulness 18. Body scan	[2, 4, 5, 12, 58–61, 63–66, 68, 79–128]
Mind body skills	1. Autogenic training 2. Biofeedback 3. Journal writing 4. Meditation 5. Deep breathing 6. Yoga 7. Reflections 8. Skills practice 9. Guided imagery	[3, 6, 54, 56, 67, 129–135]
Stress management	1. Mindful breathing 2. Mindful meditation 3. Muscle relaxation 4. Mindful awareness 5. Mindfulness based strategies 6. Guided imagery 7. Nutrition 8. Coping skills 9. Balancing life and school 10. Positive psychology 11. Support resources/content 12. Role of competition with colleagues 13. Role of conflicts with medical patients/professionals 14. Test taking skills 15. Time management 16. Assertiveness skills	[53, 69–71, 136–142]
Wellness	1. Mindful breathing 2. Muscle relaxation 3. Guided imagery 4. Nutrition 5. Coping skills 6. Positive psychology 7. Meditation 8. Time management 9. Emotional intelligence 10. Self-reflection 11. Improve wellbeing and mindfulness 12. Stressors and coping methods	[4, 96, 143–148]
Health enhancement program	1. Raise awareness of process and underpinning of stress 2. Raise awareness of process and underpinning of negative emotions 3. Raise awareness of process and underpinning of poor performance 4. Foster awareness 5. Foster conscious choice 6. Foster empathy 7. Foster behavioural change strategies	[73]
Lifestyle intervention	1. Individual reflective practices 2. Behavioural change strategies a. Physical activity b. Nutrition c. Sleep	[72–75, 149–153]
Self-development groups	1. Enhancing self-esteem 2. Identifying patterns of relationship that restricted full capacity	[154]
Visual art course	1. Strengthen observation skills 2. Recognisation and examining uncertainty 3. Recognising and examining cognitive biases 4. Promoting self-awareness 5. Expand their scope of vision 6. Strengthening capacity to tolerate ambiguity	[155–158]
Online mental health education	1. Skills for independent living 2. Academic life strategies and relationships 3. Stress management skills 4. Treatment and help seeking	[159, 160]
MAP train my brain	1. Meditation 2. Aerobic exercises	[161]
Mentorship programme	1. Provide holistic support to students	[7, 40, 162–164]
Cognitive behavioural therapy	1. Awareness of stress and its coping ways 2. Self-awareness 3. Physical methods for coping with stress 4. Exam preparation and time management 5. Training interpersonal relation skills	[55, 109, 165, 166]
Mental health program/ Psychoeducation	1. Reducing stigma associated with mental health disease 2. Discussion of current self-care practices 3. Resource book containing problem specific mental health services	[167, 168]
Unified protocol for the treatment of emotional disorders (UP)	1. Functional nature of emotions 2. Mindful emotional awareness strategies 3. Cognitive flexibility 4. Emotional avoidance and alternatives	[92]
Promoting resilience in medicine (PRIMe)	1. Meditation 2. Biofeedback 3. Visual art 4. Journal writing	[131]
Improving mental health through curricula changes	1. Pass fail system 2. Reduced contact hours across first 2 years 3. Longitudinal electives	[58, 169]
Peer support programs	1. Peer-led support of juniors in crises 2. Peer mentoring 3. Peer-led wellness initiatives	[59, 77, 170–172]
BEACCHES Orientation program	1. Panel discussion about clinician experiences of burnout and self-care 2. Educational strategies used to include exposure to novel scenarios, simulations, cultural narratives ad small group problem solving 3. Experimental clinical experience for remote beach setting 4. Indigenous culture	[173]
Mask-making exercise	1. Mask making session	[76]
DEAL model	1. Detection of stressors 2. Evaluation of stressors 3. Action toward stressors 4. Learning from stressors through self-reflection	[174]
REACH Curriculum	1. Mindfulness based training 2. Personal sharing by instructors	[175]
College student mental health education course (CSMHEC)	1. Basic theories in psychology 2. Self-awareness 3. Coping strategies to combat common mental problems	[176]
Computer assisted learning for the mind (CALM)	1. Evidence based self-help website	[78]
Vulnerability in Medicine Program (ViM)	1. Use of medical humanities 2. Recognise vulnerability in themselves 3. Reflect on their development as clinicians 4. Understand the personhood of their patients 5. Explore the therapeutic relationships	[177]
Mindfulness Based Art Workshop (MBAW)	1. Mindfulness, 2. Internal exploration, 3. Art activities	[178]
Mindfulness and Compassion Based Inter-Care Intervention	1. Cultivating inter-care resources 2. Recognising care needs for owenself, others and the community 3. Training on compassion communication skills 4. Reflection on one’s own value 5. Purpose and planning mutual care resources	[179]
Group psychological training on public health emergency response (PHER)	1. Environmental adaptation, teamwork, communication and stress relief during a public health emergency 2. Progressive relaxation training 3. Mental health education	[166]
Yoga	1. Physical and mental exercises to practice mind, body and spiritual connection	[180, 181]
“SeRenE”- Stoic Refection for Resilience and Empathy.	1. Offers a way to process the negative feelings one experiences whilst maintaining human connection 2. Predicint misfortune 3. Examining judgements 4. Developing empathetic reserves 5. Evening reflection	[182]
Balint groups	1. Consist of trained facilitator and medical students meeting to discuss mental health matters outside the clinic environment 2. Encourages thoughtful discussion and reflection on clinical encounters	[183]
Transforming Stress Program (TSP)	1. Use of Cognitive Behavioural Therapy and Didactical Behavioural Therapy models 2. Recognise their stress mindset, immediate thoughts, emotions and behaviours 3. Regulate emotions and make personal plans to cope with stress	[184]
Active Resilience Training (ART)	1. Defining resilience 2. Emotional resilience 3. Cognitive resilience 4. Physical resilience 5. Spiritual resilience 6. Practicing resilience	[185]
Counselling	1. Aimed at bringing out the best attributes of the individual leaner 2. Academic and life guidance	[186]
Transcendental mediation	1. 6 in person lectures reviewing of studies on the TM program 2. Practicing the transcendental technique 3. Personal reflective essay	[187]
Compassion Cultivation Training (CCT)	1. Guided group meditation 2. Real world assignments for practicing compassionate thought actions	[188]

The most prevalent intervention topic is mindfulness-based interventions perhaps due to growing interest in this field [58–62]; increasing social acceptability of this form of meditation [63–65]; its ease of use [4, 5, 66]; its proven efficacy in alleviating anxiety and depression amongst medical students [5, 67]; and its ability to promote attention, relaxation, and emotional intelligence [63–65]. Mindfulness-based interventions promote non-judgmental awareness and acceptance of internal and external events, thoughts, and emotions that foster the capacity to respond to situations with equanimity [63–65]. The versatile nature of this form of meditation sees it used in a variety of self-care interventions, including mindful breathing, mindful eating, mindful yoga [4, 5, 66], managing reflexivity, reflective listening, and journaling [4, 5, 66].

However, mindfulness is not a ‘one-size-fits-all’ solution and may not be uniformly accepted nor applicable to all users [68]. When poorly supported or inculcated within appropriate settings, it may precipitate negative effects [58]. Engaging in mindfulness may also pose a challenge for acutely stressed or anxious students [5, 67]. Reviews on mindfulness are also divided on its overall efficacy [58–61].

Other stress management interventions [59, 62] are also proffered. These interventions tend to inculcate elements of mindfulness and focus on instilling more effective coping mechanisms, recognizing the symptoms of stress, and capturing the negative effects of stress on their learning, personal health and patient care [69–71]. Additional interventions include lifestyle interventions, such as increasing physical activity and improving eating habits and sleep quality [72–75]. More recently, psychoeducation or the use of activities, such as mask making to promote self-reflection and development of personal identity, have been adopted [76–78].

### Domain 3: pedagogy

Current self-care interventions vary in duration, place in the curriculum, group size, facilitator, and delivery methods. The features of pedagogy utilized are illustrated in Table 3.

**Table 3:** Pedagogy used

Element		References
Duration of program	Weeks (under a month)	[55, 81, 92, 110, 146, 173, 174] [157, 168, 179, 182, 189]
	Months (Under a year)	[2–6, 54, 56, 63, 66–68, 70–73, 78, 80, 82–91, 93–95, 99, 100, 110–114, 129–132, 134–136, 138–140, 142–145, 149, 154, 155, 159, 161, 167, 170] [49, 60, 115, 118–125, 127, 128, 158, 172, 177, 180, 184, 188, 190, 191]
	Years	[7, 64, 65, 75, 97, 101, 148, 166, 169]
Year of study	1	[3, 7, 49, 54, 73, 80, 87, 88, 94, 115, 128, 136–138, 140, 143, 144, 155, 159, 169, 172, 184]
	2	[65, 125, 132, 149]
	3	[111, 154, 166, 177, 182]
	4	[2, 83, 95, 158, 167]
	Multiple years	[4–6, 55, 56, 63, 64, 66, 67, 70–72, 75, 76, 78, 81, 82, 84–86, 89–93, 96–101, 110, 112–114, 118–121, 123, 124, 127, 130, 131, 139, 142, 145, 146, 157, 161, 168, 170, 173, 174, 178, 179, 189]
Group size	Individual	[53, 56, 78, 84, 98, 99, 129, 138, 155]
	Small group (< 10 students)	[66, 84, 96, 115, 137, 139, 154, 166, 172, 177, 178, 188]
	Large group (> or = 10 students)	[3–7, 54, 55, 63–65, 67, 71–73, 75, 76, 80, 82, 83, 85–89, 91, 93–97, 113, 114, 130–132, 136, 140, 142–146, 149, 159, 167, 169, 170, 173, 174] [49, 60, 118–124, 128, 165, 168, 180, 184, 189, 190]
Delivery of program	Peer mentor	[81, 90, 115, 127, 139, 166, 167, 172, 178]
	Faculty members	[3, 6, 54, 67, 72, 73, 87, 91, 96, 130, 146, 161]
	Doctor, including psychiatrists	[64, 66, 75, 76, 83, 84, 92, 95, 96, 136, 143, 154, 169, 170, 177, 188]
	Nurses	[96, 144]
	Allied healthcare workers: social workers, occupational therapists, dietitians	[64, 72]
	Psychologists and psychotherapists	[5, 55, 63, 64, 80, 85, 89, 121, 136, 143, 144, 165, 166, 170, 179, 182, 184]
	Counsellor	[136, 143, 166, 179]
	Trained facilitators	[4, 49, 56, 70, 89, 94, 96, 110, 113, 114, 119, 120, 123, 127, 132, 140, 155, 157, 179, 187]
	Physical education instructors	[63]
	Researchers	[96, 111]
	Experts and healthcare workers (not further specified)	[88, 96, 138, 159]
Delivery method	Face to Face teachings (not otherwise specified)	[2–7, 55, 56, 63–65, 67, 70–73, 76, 80–85, 88–91, 94–97, 111, 112, 114, 115, 119, 120, 122, 124, 125, 130–132, 136, 137, 139, 143–145, 154, 155, 157, 158, 161, 165–167, 170, 172–174, 177, 180, 184, 187, 188] [49, 190]
	Lecture setting	[75, 86, 87, 92, 110, 112, 113, 149, 159, 170]
	Classroom setting	[54, 66, 93, 140, 142]
	Online	[64, 68, 78, 100, 118, 121–123, 129, 138, 157, 158, 168, 178–180, 182, 189]
	Mobile application	[99]
	DVD/CD/audiocassette	[65, 84, 85, 90, 98, 110, 112]
	Resource book	[167]
	Curriculum changes	[169]

Much of the debate on self-care pedagogy is premised on whether it should be voluntary or mandatory. Whilst mandatory self-care interventions maximize audience reach, such actions may render them counterproductive [113, 131]. When made mandatory, medical students may perceive it as a violation of their autonomy. This could reduce engagement and precipitate stress and feelings of resentment and coercion [4, 5, 64, 65, 87, 90, 111, 131, 137, 139]. Proponents of voluntary participation in self-care programs also argue that the effectiveness of such interventions far outweighs greater audience reach—in turn boosting active participation and enhanced engagement and better outcomes [113, 131].

### Domain 4: influences

Factors facilitating or hindering the success of self-care interventions occur at the student or program level. These are summarized in Table 4. Both options rely on the choice of program delivery, contextual considerations, approach, and the presence of a conducive environment that facilitates active and open sharing [154, 155]. A conducive environment is also inclusive of protected time to attend and actively engage in these interventions [68, 137, 161]. Indeed, when poorly supported, these programs become an additional source of stress [91, 145, 173].

**Table 4:** Influences upon self-care education

Facilitators of Success		References
Student-level	Interested students. • Older, with a greater level of maturity and appreciation for the need for self-care interventions • Students with higher stress levels pre-intervention	[78, 84, 129, 143, 174]
	Students’ adherence to intervention	[3, 7, 58, 64, 85, 89, 90, 99, 136]
	Voluntary participation	[54, 68, 76, 89, 114, 115, 132, 146, 178]
Program-level	Validated program	[123, 139]
	Occurred during more conducive periods	[49, 66, 75, 86, 114]
	Provision of individualised options	[160]
	Provision of incentives (e.g., financial, academic points)	[153, 158, 179]
	Safe space • Small group format • Open, non-judgmental, collaborative discussion to destigmatize mental health concerns • Personal sharing of own struggles • Empathetic facilitators • Peer/clinician led class • Inter-professional learning to breakdown traditional hierarchies between professions	[7, 56, 58, 64, 65, 83, 115, 138, 139, 144, 145, 154, 155, 167, 168, 177]
	Large group size or single session to stay within resource constraints	[68, 84, 87, 90, 92, 161]
	Continuous feedback and improvement processes	[58, 139]
	Tangible end product creating a sense of accomplishment	[158]
	Readily available online material (e.g., videos, audiotapes, readings) for students to learn at own pace, anonymously and to reach rural areas	[68, 100, 138, 159]
**Barriers to Success**		**References**
Student-level	Low adherence (e.g., due to personal stress)	[5, 6, 49, 63, 64, 67, 80, 85, 87, 90, 100, 115, 118, 121, 123, 126, 131, 149, 158, 179]
	Mandatory participation (e.g., leading to resentment for program)	[58, 113, 116, 131, 192]
	Lack of anonymity and concern about stigma	[58, 121, 160, 193]
	Poor understanding of intervention by students	[5, 82, 91, 126, 129]
Program-level	Did not cater to individual preference of self-care practices	[5–7, 116, 192, 193]
	Large group size	[75, 136]
	Poor scheduling (e.g. Scheduled near or in exam periods)_	[116, 192]
	Treating wellness as a skill which in doing so stigmatises medical learners and harm their wellness	[192]
	Targeting learners instead of learning environment sending the message that they are the problem and not the system	[192]
	Hierarchy between facilitators and students	[90, 138]
	Lack of support • Lack of faculty/trained facilitators • Lack of time allocated to discuss/practice intervention; intervention too short or infrequent	[5–7, 56, 58, 67, 68, 75, 90, 91, 96, 131, 137, 143, 145, 155, 161, 166, 173, 180]

At the student level, interest and adherence are pivotal facilitators to effective self-care interventions. Conversely, poor understanding of the interventions, mandatory participation and low adherence hinder success.

At a program level, smaller peer or clinician-led sessions are more successful in facilitating open, safe and collaborative discussions [154, 155]. Contrarily, large group sizes and the lack of time and resources impede engagement in these programs [68, 137, 161] and in some cases, become an additional curricular demand [91, 145, 173].

### Domain 5: outcomes

The positive impacts of self-care education are illustrated in Table 5. However, some reviews reveal equivocal or even negative outcomes [58]. The effects are categorized into student and patient levels.

**Table 5:** Benefits of self-care education

Benefits of Self-care Education		References
Student-Level	Improved ability to cope with negative emotions (e.g., stress, anxiety)	[55, 67, 71, 100, 119, 122–124, 138, 165, 166, 174, 180] [49, 59, 126, 177]
	Improvement in psychological symptoms • Anxiety • Stress • Distress • Hostility • Exhaustion • Sleep quality • Self-esteem • Burnout • Improved relaxation	[2, 3, 5, 6, 55, 56, 60, 65, 69–73, 76, 80, 81, 83, 84, 86, 88, 90–95, 98–101, 110, 111, 114, 115, 117, 118, 128–132, 135, 136, 140, 143, 154, 157, 159, 161, 166, 169, 170, 174, 177–180, 184, 185, 187, 188, 190, 194]
	Decrease in inappropriate coping mechanisms (e.g., drug, alcohol use)	[2, 3, 5, 6, 69, 71, 80, 81, 86, 88, 90, 93, 99, 101, 116, 131, 132, 136, 140, 143, 154, 158, 159, 165, 169, 170, 174, 184, 185]
	Decrease in mental health crises (e.g., suicide)	[2, 3, 5, 6, 69, 71, 80, 81, 86, 88, 90, 93, 99, 101, 131, 132, 136, 140, 143, 154, 159, 169, 170, 174]
	Improvement in physical health (e.g., nutrition, balanced diet)	[72, 125, 146, 149]
	Improvement in values and skills • Increased reflection ability • Increased self-regulation • Increased self-compassion • Increased empathy • Increased spirituality • Increased mindfulness/mediation	[59, 67, 86, 93, 119, 122, 129, 132, 136, 138, 172, 182, 187, 188, 193]
	Improvement in professional practice and academics • Increased study engagement • More positive study experience • Academic result • Increased ability to counsel patients on positive behavioural change, complementary and alternative medicine • Ethical conduct • Decrease dropout rates	[3, 6, 54, 55, 67–69, 75, 88, 94, 111, 122, 129, 146, 157, 182, 183, 187]
	Improvement in wellbeing • Improved quality of life • Increased community support	[6, 67, 73, 117, 118, 123, 125, 130, 167–169, 179, 187, 190]
	Creating a sense of community	[118–120, 172]
	Increased knowledge of mental health and help-seeking behaviour	[54, 71, 159, 168]
Patient-Level	Improvements in • Patient safety • Quality of care • Patient satisfaction	[2, 4–6, 93, 94, 96, 130, 137, 167, 177, 180]

At the student level, self-care programs enhance student wellbeing, reduce psychological distress and effects, inculcate positive values and skills, and increase academic performance [67, 88, 93, 111, 129, 132, 138, 140]. At the patient level, there is an improvement in patient safety, quality of care and patient satisfaction [2, 4–6, 93, 94, 96, 130, 137, 167].

### Domain 6: outcome assessment method

Current assessment methods are listed in Table 6. Most studies employ validated questionnaires, wherein the Perceived Stress Scale presents the most common tool used, as observed in 16 studies [66, 80, 83–85, 88, 91, 94, 95, 110, 130, 137, 159, 161, 169, 174]. Four studies utilize interviews [82, 91, 96, 111] whilst two studies employ laboratory tests, such as measuring salivary cortisol [3, 140], as their methods of assessment. The remaining studies adopt non-validated questionnaires and surveys [5, 54, 56, 75, 76, 84, 86, 87, 113, 131, 139, 145, 149].

**Table 6:** Methods of assessment

Categories	Intervention/Methods	References
Laboratory Test	1. Measuring salivary cortisol dehydroepiandrosterone-sulphate (DHEA-S), testosterone, and secretory immunoglobulin A (sIgA)	[3, 140]
Interview	1. Face to Face interviews 2. Semi-structured interviews	[82, 91, 96, 111, 119, 126, 182]
Non-validated questionnaire /Survey	1. Feeback questionnaire 2. Outcome assessment survey 3. Mindfuless impact 4. Behaviour change plan 5. Evaluation form 6. Particpant course evaluation survey 7. Group lead couse evaluation survey 8. Compliance 9. Reflective essay	[5, 54, 56, 75, 76, 84, 86, 87, 113, 115, 131, 139, 145, 149, 153, 160, 185–187]
Validated questionnaire /Survey	1. Multidimensional Experiential Avoidance Questionnaire [92] 2. Quality of Life Enjoyment and Satisfaction Questionairre [92] 3. Rosenberg Self-Esteem Scale [89, 92] 4. Interpersonal Reactivity Index [94, 131, 190, 192] 5. Overall Anxiety Severity and Impairment Scale [92] 6. Beck Depression Inventory [92, 136, 143] 7. Beck anxiety inventory [110, 136, 143] 8. Jefferson scale of physcal empathy [64, 67, 91, 174, 180, 182] 9. Cohen’s Perceived Stress Scale [67] 10. Self-Regulation Questionnaire [67, 180] 11. Self-Compassion Scale [67, 80, 174, 180, 188] 12. Coping Self-effiacy scale [136] 13. Social Readjustment Rating Scale Revised [136] 14. Duke religion index [87] 15. DASS 21—Depression, Anxiety, and Stress Scale [84, 87, 95, 110, 122, 165, 181, 188, 194] 16. WHOQOL-BREF—World Health Organization Quality of Life [73, 87] 17. Five Facets of Mindfulness (FFMQ (99)-BR) [87, 91, 122, 188] 18. Maslach Burnout Inventory (MBI) [85, 137, 174, 188] 19. Medical Outcomes Study Short Form (SF-8) [137] 20. Perceived Stress Scale (PSS) [66, 80, 83–85, 88, 91, 94, 95, 110, 130, 137, 157, 159, 161, 169, 174] [122, 123] 21. Connor Davidson Resilience Scale (CD-RISC) [64, 137] 22. Happiness and Gratitude Scale [137] 23. Resilience Scale (RS) [80] 24. Groningen Reflection Ability Scale (GRAS) [155] 25. Tolerance for Ambiguity (TFA) [155] 26. Best Intentions Questionnaire (BIQ) [155] 27. Cognitive and Affective Mindfulness Scale-Revised (CAMS-R) [56] 28. The Symptom Checklist-90-Revised1 (SCL-90-R) [70, 73, 85, 110, 122] 29. General Severity Index (GSI) [73] 30. Perceived Medical School Stress (PMSS) [154] 31. Hopkins Symptom Check List (SCL-5) [154, 180] 32. Mental Health Continuum-Short Form (MHC-SF) [88] 33. Utrecht Work Engagement Scale for Students (UWES-S) [88] 34. .Freiburg Mindfulness Inventory (FMI) [88, 89, 96] 35. Mindfulness Adherence Questionnaire (MAQ) [88] 36. Mindfulness Scale–Revised (CAMS-R) [129, 130] 37. Neff’s self-compassion scale [129] 38. 10-item Calm, Compassionate Care Scale [129] 39. Smith’s 6-item Brie Resilience Scale [129] 40. 5-item Santa Clara Brief Compassion Scale [129] 41. Mindful Attention Awareness Scale (MAAS) [95, 101, 114, 122, 157, 159, 165, 194] 42. General Health Questionnaire (GHQ-12) [94, 95, 146, 159] 43. Subjective Happiness Scale (SHS) [95] 44. Satisfaction With Life Scale (SWLS) [95] 45. Distress tolerance scale (DTS) [130] 46. Positive affect negative affect schedule (PANAS) [130] 47. Trier Inventory for the Assessment of Chronic Stress (TICS) [89, 159] 48. Brief COPE [89] [63, 142] 49. Brief Symptom Inventory (BSI) [63, 89, 122] 50. Response Style Questionnaire (RSQ) [89] 51. Skala Impulsives-Verhalten-8 (I-8) [89] 52. Frost Multidimensional Perfectionism Scale (FMPS) [89] 53. Short Scale for Measuring General Self-efficacy Beliefs (ASKU) [89] 54. Satisfaction with Life Scale (SWLS) [89] 55. Kala Internale-Externale-Kontrollüberzeugung (IE-4) [89] 56. The Help-seeking questionnaire [159] 57. Client Satisfaction Questionnaire (CSQ-I) [159] 58. The Stanford Personal Health Questionnaire Depres-sion Scale11 (PHQ-8) [91, 161] 59. Ruminative Responses Scale13 (RRS) [161] 60. The Quality of Life Scale14 (QOLS) [161] 61. Maslach Burnout Inventory [71, 110, 114] 62. The Coping Strategies Inventory [71] 63. Social Readjustment Rating Scale-Revised [143] 64. GAD7 [72, 78, 90] 65. PHQ9 [72, 78, 90] 66. Linear Analogue Self-Assessment [LASA], resilience [90] 67. RS15 [90] 68. Perceived Competence Scale [90] 69. Academic motivation (the Motivated Strategies for Learning Questionnaire [90] 70. Profile of Mood States (POMS) [65] 71. Student Self- Efficacy Questionnaire [140] 72. Brief Job Stress Questionnaire (BJSQ) [140] 73. General Self-Efficacy Scale (GSE) [84] 74. Warwick-Edinburgh Mental Well-being Scale (WEMWBS) [96]) [178] 75. Coping checklist [97] 76. Center for Epidemiological Studies Depression Scale [94, 169] 77. the Spielberger State-Trait Anxiety Inventory [169] 78. The Perceived Cohesion Scale [169] 79. Association of American Medical [169] 80. Colleges’ Graduation Questionnaire (GQ) [169] 81. Likert-type educational outcomes survey [91] 82. Empathy Construct Rating Scale (ECRS) [70] 83. State-Trait Anxiety Inventory [70] 84. Index of Core Spiritual Experiences [70] 85. Yonki task [114] 86. The Mayer Solvey Caruso Emotional Intelligence Test [114] 87. General Well-Being Schedule (GWBS) [99] 88. CS compassion scale [174] 89. Mental Health Continuum - Short Form [110] 90. Self-harm Behaviour Questionnaire [110] 91. Suicidal Ideation Questionnaire [110] 92. Suicide Behaviours Questionnaire [110] 93. Life Satisfaction Questionnaire [110] 94. Compassion Scale pommier (CSP) [188] 95. Pemberton Happines Index (PHI) [188] 96. Difficulties in Emotion Regulation Scale (DERS) [188, 194] 97. Brief Resilience Scale (BRS) [182, 188] 98. Situational Self-Awareness Scale (SSAS), [157] 99. Chronic Conditions Survey [171] 100. Medical Student Stress Questionnaire [165] 101. The State-Trait Anxiety Inventory (STAI-1 Form) [122, 123, 178, 180] 102. Ryff’s Psychological Well-Being Questionnaire [124] 103. Zung’s Self-rating Anxiety Scale (SAS) [166] 104. Zung’s Self-rating Depression Scale (SDS) [166] 105. The Somatic Self-rating Scale (SSS) [166] 106. The trait coping style questionnaire (TCSQ) [166] 107. Visual Analogue Scale (VAS) [125] 108. Sleep Quality Scale (SQS)20; modified version of the UCLA LS-8 scale21 [125] 109. NIH patient reported outcomes measurement information system (PROMIS) [178]	

### Stage 5 of SEBA: analysis of evidence-based and non-data driven literature

Evidenced-based data from bibliographic databases (henceforth evidence-based publications) were separated from grey literature and opinion, perspectives, editorial, letters and non-data-based articles drawn from bibliographic databases (henceforth non-data driven literature). The two groups of data were thematically analysed separately. The themes/categories from both groups were then compared against each other to determine if there were additional themes in the non-data driven group that could influence the narrative.

There was consensus that themes from the non-data driven and peer-reviewed evidence-based publications were similar and did not bias the analysis untowardly.

## Discussion

### Stage 6: synthesis of discussion

In answering its primary and secondary research questions, *“What is known about self-care education interventions amongst medical students?”*, this SSR in SEBA provides a sketch of the current state of self-care education in medical school curricula. Each key aspect is considered by its secondary research questions. Here, the secondary research questions, *“What topics are included in self-care education in medical schools?”*, *“How are self-care education interventions structured in medical schools?”*, *“How is self-care in medical school assessed?”* and *“What factors support and hinder self-care education interventions in medical schools?”* highlight the topics and interventions used in Domain 2 (Table 2), the pedagogy employed in Domain 3 (Table 3), the influences upon the training processes in Domain 4 (Table 4) and the outcomes and outcome assessment methods in Domains 5 and 6 (Tables 5 and 6) respectively.

In answering its secondary research question, *“How are self-care education interventions structured in medical schools?”,* current data suggests that such programs should be provided a formal place within the curriculum, accompanied by the provision of trained tutors, protected time for engagement, an appropriate setting, and opportunities for debriefs [5–7, 67]. It is likely that mandatory sessions will lack the desired effects but greater education on the matter would be useful to allow students to make an informed decision on participating [113, 131]. Programs should also provide general and personalized information on self-care. General education ought to cater to the goals of the program, the group size [64, 138, 139, 167], and the setting [6, 91, 131, 173] whilst individualized advice must consider the specific needs [64, 138, 139, 167], motivations [4, 5, 64, 65, 87, 131, 137, 139] and abilities of individual medical students.

Similarly, available resources should also be accounted for where considerations are made with regards to the training environment [68, 100, 138], structure [90, 111, 131], assessment methods and outcome measures [122, 123], as well as tutor support available. Critically, at a program-level, self-care education sessions must be supplemented with role modelling, mentoring, supervision and coaching to provide timely, personalized, appropriate, holistic guidance, support and remediation [5–7, 56, 67, 68, 75, 90, 91, 96, 131, 137, 143, 145, 155, 161, 173]. Faculty development and the presence of dedicated facilitators must also be a key consideration.

Returning to the context of medical students who are frequently exposed to patient death and suffering where psycho-existential distress has been recognized, awareness about issues on mental and emotional health should be raised. This then necessitates the availability and access to self-care interventions for those who choose to engage in these programs. We also underscore the importance of ensuring that there is sufficient time and support allocated to these programs, as well as effective means of providing longitudinal support post-medical school.

### Limitations

Focus upon guidelines published in English may have restricted the search results whilst data drawn from North America and the European countries may not be necessarily transferable beyond these regions where education, healthcare programs and healthcare financing differ.

## Conclusions

Whilst awareness of mental health issues ought to be underscored, as should its role in professionalism, and access to self-care education and interventions be made easy for those who choose to engage in these practices, we believe that one area of urgent concern is tutor training. Tutors who are expected to access and support students should be provided training and longitudinal support. Similar ties and access to psychological and psychiatric medical services, formal debriefs, coaching, remediation, and supervision programs should be made clear. Further study in changing the culture and perspectives of self-care and mental and psycho-emotional well-being in medicine should be the focus of future studies, as should the design of effective assessment tools.

### Supplementary Information

**Additional file 1.**

**Additional file 2.**

**Additional file 3.**

## Data Availability

All data generated or analysed during this review are included in this published article and its supplementary files.
