# Supplementary material for: Enhancing self-care education amongst medical students: a systematic scoping review
Source: BMC Med Educ. 2024 Jan 8;24:37. doi: 10.1186/s12909-023-04965-z (PMC10773141; doi:10.1186/s12909-023-04965-z)
Supplement: Supplementary file 2 — Additional file 2. [file 12909_2023_4965_MOESM2_ESM.docx]

**Additional File 2. Full Search Strategy**

**Date Range:** 1^st^ January 2000 – 30^st^ June 2023

| **PubMed** | **(("Education, Medical" [Mesh] OR "Schools, Medical" [MeSH] OR "Students, Medical" [MeSH] OR medical student*[tiab] OR medical school[tiab] OR medical schools[tiab] OR medical education*[tiab] OR medical undergraduate*[tiab] OR medical postgraduate*[tiab]))**  **AND**  **(("Self-Care"[MeSH] OR "Mindfulness"[MeSH] OR Selfcare[tiab] OR Self-care[tiab] OR Student welfare[tiab] OR Mindfulness[tiab] OR "anxiety management"[tiab] OR "Stress management"[tiab] OR "time management"[tiab))** |
| --- | --- |
| **Embase** | (('medical education'/exp OR 'medical school'/exp OR 'medical student'/exp OR 'medical student*':ab,ti OR 'medical school':ab,ti OR 'medical schools':ab,ti OR 'medical education*':ab,ti OR 'medical undergraduate*':ab,ti OR 'medical postgraduate*':ab,ti))  AND  (('self care'/exp OR 'mindfulness'/exp OR 'selfcare':ab,ti OR 'self-care':ab,ti OR 'student welfare':ab,ti OR 'mindfulness':ab,ti OR 'stress management':ab,ti OR 'anxiety management':ab,ti OR 'time management':ab,ti)) |
| **Psychinfo** | (exp Schools, Medical/ OR exp Students, Medical/ OR exp Education, Medical/ OR exp Education, Medical, Undergraduate/ OR ("medical student" OR "medical school" OR "medical schools" OR "medical education" OR "medical undergraduate" OR "medical postgraduate*").ti,ab.)  AND  (exp Self Care/ OR exp Mindfulness/ OR ("Selfcare" OR "Self-care" OR "Student welfare" OR "Mindfulness" OR "Anxiety management" OR "stress management" OR "time management").ti,ab.) |
| **ERIC** | (MAINSUBJECT.EXACT("Medical Education") OR MAINSUBJECT.EXACT("Medical Schools") OR MAINSUBJECT.EXACT("Medical Students") OR TI,AB (medical student* OR medical school OR medical schools OR medical education* OR medical undergraduate* OR medical postgraduate*))  AND   MAINSUBJECT.EXACT("Daily Living Skills") OR MAINSUBJECT.EXACT("Metacognition") OR MAINSUBJECT.EXACT("Stress Management") OR TI,AB(Selfcare OR Self-care OR Student welfare: OR Mindfulness OR Stress management OR Anxiety management OR time management)) |
| **Scopus** | TITLE-ABS-KEY ( ( "medical student*"  OR  "medical school"  OR  "medical schools"  OR  "medical education*"  OR  "medical undergraduate*"  OR  "medical postgraduate*" )  W/15   ( selfcare  OR  "self-care"  OR  "Student welfare"  OR  mindfulness  OR  "anxiety management"  OR  "stress management"  OR  "time management" ) ) |
| **Google Scholar** | allintitle: "medical student" OR "medical students" OR "medical education" OR "medical school" OR "medical schools" OR "medical school" "self care”  allintitle: "medical student" OR "medical students" OR "medical education" OR "medical school" OR "medical schools" OR "medical school" "mindfulness" |
