# Supplementary material for: Enhancing self-care education amongst medical students: a systematic scoping review
Source: BMC Med Educ. 2024 Jan 8;24:37. doi: 10.1186/s12909-023-04965-z (PMC10773141; doi:10.1186/s12909-023-04965-z)
Supplement: Supplementary file 3 — Additional file 3. [file 12909_2023_4965_MOESM3_ESM.docx]

**Additional File 3. Tabulated Summaries of Included Articles**

Number of included articles: 147

| Author/Year | Article Title | Type of study | MERSQI | COREQ | Study aim | Methodology | Key findings | Proposed solutions/conclusions |
| --- | --- | --- | --- | --- | --- | --- | --- | --- |
| Hassed et al 2009 | Enhancing the health of medical students: outcomes of an integrated mindfulness and lifestyle program | Quasi-Experimental Study | 13 | NA | Study aims to equip students with an integrated mindfulness and lifestyle program which improves student wellness and self-care before exams. | Study investigated effects of the program on psychological distress and quality of life among medical students. Using the following assessment tools: the depression, anxiety, and hostility subscales of the Symptom Checklist-90-R (including the Global Severity Index - GSI) and the WHO Quality of Life (WHOQOL) questionnaire. Data was collected pre-course (T1) during mid-semester and post-course (T2) before the exam week. | Positive enhancements in student well-being were observed across all measures, with statistically significant improvements noted for the depression, hostility. However, the anxiety subscale did not show significant changes. Significant outcomes were also identified in the psychological domain of the WHOQOL, but not in the physical domain. | While this study's results suggest that this intervention is effective, there are several limitations of the study design. Such as a short follow-up duration and an absence of a control group. Subsequent research should focus on delineating the specific influence of distinct program components, investigating long-term effects, and assessing how these interventions might shape future perspectives and clinical practices. |
| Mari Holm et al. 2010 | Self-development groups reduce medical school stress: a controlled intervention study | Non-Randomised Control Trial | 15 | NA | Study aims to examine the effectiveness of group interventions specially designed to mitigate distress during medical education. | 129 third year medical students were allocated to intervention and 153 to controls. The intervention group had access to two types of group intervention sessions. Self-development groups led by trained group psychotherapists and discussion groups facilitated by experienced general practitioners, focusing on doctor-relevant themes. Data was collected before the intervention (T1) and three months after (T2). Perceived Medical School Stress (PMSS) and Symptom Check List-5 (SCL-5) assessments were employed to measure distress levels. | The intervention group displayed a decrease in PMSS, while the control group maintained their PMSS levels. An adjusted multiple regression analysis, accounting for age and sex, indicated that the intervention significantly predicted a reduction in PMSS. When comparing the effects of self-development and discussion groups to the control group, the self-development group emerged as the sole significant predictor of PMSS reduction. There also was no observed reduction in overall mental distress over the same period. | After a three-month follow-up, it was evident that the intervention had a favourable impact on how students perceived stress related to medical school. Subsequent analyses revealed that this effect could be attributed specifically to the involvement of students in self-development groups. |
| Kakoschke et al. 2021 | The importance of formal versus informal mindfulness practice for enhancing psychological wellbeing and study engagement in a medical student cohort with a 5-week mindfulness-based lifestyle program | Quasi-Experimental Study | 10.5 | NA | Study aimed to explore the effects of a 5-week mindfulness-based course on student’s mental health, perceived stress, study engagement, dispositional mindfulness and whether the improvements correlated with the extent of formal and or informal mindfulness practice. | The study involved first-year medical students. Out of these, 205 individuals completed both pre- and post-course questionnaires within a 5-week mindfulness-based lifestyle intervention. Before and after the intervention, participants filled out several assessment tools, including the Mental Health Continuum-Short Form, the Perceived Stress Scale, the Utrecht Work Engagement Scale for Students, the Freiburg Mindfulness Inventory, and the Mindfulness Adherence Questionnaire. | Significant enhancements were observed in mental health, perceived stress, study engagement, and mindfulness scores. These improvements in were interconnected,for example, increases in Perceived Stress Scale (PSS) change scores exhibited negative correlations with all other change scores. Moreover, the improvements in these outcomes were positively linked to the quality of informal mindfulness practice, while improved FMI scores were associated with formal practice. | Participating in a 5-week mindfulness-based program is linked to enhancing psychological well-being and study engagement among medical students. Notably, these improvements are more pronounced when students engage in informal mindfulness practices as opposed to formal ones. |
| Kemper et al. 2015 | What Is the Impact of Online Training in Mind–Body Skills? | Prospective Cohort Trial | 13 | NA | Study aimed at assessing the impact of online Mind-Body Skills training on trainees’ stress, mindfulness, and confidence in providing calm, compassionate care. | Study utilized a prospective cohort trial design. Participants included trainees entering medical school, graduate programs in nursing, social work, and dietetics, as well as residents in family medicine and paediatrics. These individuals were invited to partake in online surveys before and 12 weeks after enrolling in elective integrative health courses centred around Mind–body skills (MBS) training. The elective courses had no credit value and were not bound by any mandatory deadlines for completion. | At initiation, the group of 60 individuals who participated in MBS training was comparable to the group of 43 who did not engage in terms of profession, gender, perceived stress levels, mindfulness, resilience, and compassion. After a span of twelve weeks, those who had taken part in MBS training exhibited notably greater improvements in measures related to stress reduction, mindfulness, and confidence in delivering calm and compassionate care compared to those who did not participate. | The online elective proves to be a viable approach for enhancing mindfulness, alleviating stress, and boosting confidence in offering composed and compassionate care. Further research is essential to ascertain the effects of mandatory versus optional courses, the most effective frequency and content of training, and the costs and benefits of online versus in-person training. |
| Keng et al. 2015 | Effects of a Brief Mindfulness- Based Intervention Program on Psychological Symptoms and Well-Being Among Medical Students in Malaysia: A Controlled Study | Randomised Control Trial | 15.5 | NA | Study aimed at assessing the effects of a 4-week mindfulness-based intervention on psychological health among medical students and if the effects were moderated by mindfulness. | 139 students participated in the study and completed measures of mindfulness and psychological symptoms pre- and post-intervention. | The results of the analysis indicated that the group receiving the intervention exhibited notably more substantial enhancements in multiple aspects: depressive symptoms, anxiety levels, general psychiatric symptoms, perceived stress, subjective happiness, and life satisfaction, in comparison to the control group. Notably, individuals with lower trait mindfulness at the study's outset displayed greater improvements in depressive and anxiety symptoms. Moreover, changes in mindfulness were identified as a mediator for various outcomes | The outcomes suggest that a short mindfulness-based intervention program effectively decreases psychological symptoms within medical students. It's conceivable that mindfulness plays a crucial role as a mechanism driving the changes observed due to the intervention. |
| Kraemera et al. 2016 | Mind–Body Skills Training to Improve Distress Tolerance in Medical Students: A Pilot Study | Non-Randomised Control Trial | 13 | NA | The aim of this study was to determine the impact of a mind-body skills training course on the distress tolerance of medical students. | Study involved 52 first- and second-year medical students. Participants were divided into either a mind-body group or a control group. The intervention consisted of an 11-week training group with each session lasting 2 hours. The participants completed mixed methods self-reported assessment before and after the 11-week period. | Student’s participating in the mind-body group demonstrated moderate enhancement in all facets of distress tolerances subscales over time. They also provided qualitative feedback indicating an enhanced capacity to manage emotional distress. The correlation between psychological symptoms and improvements in distress tolerance was observed in the intervention group but not the control. | These initial results offer backing for the idea that enhancing distress tolerance via mind–body skills training could potentially shield medical students from the detrimental effects of psychological distress on their functioning. Consequently, integrating mind–body skills training into medical school curricula might play a role in enhancing the mental well-being of medical students. However, further research employing more robust methodologies is needed to build upon these findings and draw more conclusive insights. |
| Kuhlmann et al.2015 | A mindfulness-based stress prevention training for medical students (MediMind): study protocol for a randomized controlled trial | Study protocol for Randomised Control Trial | NA | NA | Study aims to determine the effectiveness of a specifically developed mindfulness-based stress prevention training for medical students that includes selected elements of cognitive behavioural strategies. | This study is a randomized controlled trial with four evaluation points: baseline, post-intervention, one-year follow-up, and five-year follow-up. The participants were randomly assigned to one of three groups: MediMind, Autogenic Training, or a control group. Eligible participants are medical or dental students. The intended final sample size is 126 participants. The training sessions consist of five weekly sessions. After the five-year follow-up, MediMind will be provided to the control group participants. Descriptive and inferential statistical analysis were employed. | NA | Possible limitations are the voluntary participation, potential for attrition over time particularly among individuals assigned to the control group. On the positive side, the study design boasts several strengths. These include the use of random allocation, which enhances the reliability of the results, the incorporation of follow-up assessments to track changes over time, the inclusion of control groups to enable comparisons, and the involvement of participants at various stages of medical training, facilitating the potential for nuanced analyses based on these different stages. |
| Kuhlmann et al. 2016 | Coping with stress in medical students: results of a randomized controlled trial using a mindfulness-based stress prevention training (MediMind | Randomised Control Trial | 11 | NA | Study aims to determine the effectiveness of a specifically developed mindfulness-based stress prevention training for medical students that includes selected elements of cognitive behavioural strategies. | Prospective randomized controlled trial with three groups:(MediMind), standard treatment (Autogenic Training) and a control group without treatment. The population consisted of medical or dental students. The participants filled out self-report questionnaires at three time points: initially, post-training, and during a one-year follow-up. The primary focus was on measuring distress using the Trier Inventory for the Assessment of Chronic Stress (TICS), along with coping strategies through the Brief COPE, which was treated as a co-primary measure. Additionally, the impact on psychological distress (evaluated by the Brief Symptom Inventory, BSI) was anticipated to manifest a year after the training sessions. | At the beginning, 183 students were randomly assigned to different study groups. After one year, 80 participants were included in the analysis: 31 in the Medi Mind group, 32 in the Autogenic Training group, and 17 in the control group. There was a noticeable dropout of students with higher psychological symptoms (p = .020). Analysing the data using MANCOVA on TICS and Brief COPE didn't show significant interaction effects. However, concerning the BSI, there was a significant overall interaction effect (p = .002, η2partial = .382), although subsequent detailed analyses didn't yield significant results. The average Global Severity Index (BSI) scores hinted that Medi Mind might be linked to a reduction in psychological distress. | Because of the elevated and specific dropout percentages, it's challenging to extend the findings to a broader population, necessitating additional research. Despite a notable engagement in the training programs, there appears to be a requirement for more preventive initiatives. The study provides significant insights into how to effectively introduce and assess stress prevention within medical schools. |
| Kurki et al. 2021 | Digital mental health literacy -program for the first-year medical students’ wellbeing: a one group quasi-experimental study | Non-Randomised Control Trial | 14 | NA | Study aimed to assess the effectiveness of digital transitions, a MHL program for medical students that covered blended life skills and mindfulness activities. | Quasi-experimental study including 374 first year medical students. Intervention included two 60-minute lectures, four weeks apart with online self-learning material in between. Mental health knowledge, stigma and help seeking tendencies were assessed using respective questionnaires to gauge mental health literacy. The Perceived Stress Scale and General Health Questionnaire were employed to evaluated student’s stress levels and overall health. | Students' average mental health knowledge scores exhibited improvement and their emotional symptoms were immediately alleviated. These improvements remained consistent during the two-month follow-up. While the students' stress levels initially decreased, and their attitudes towards seeking help improved significantly after the program, these changes were not sustained at the two-month follow-up. However, there was no significant alteration in the stigma associated with mental illness throughout the study. | The digital Transitions program was seamlessly incorporated into the university curriculum and led to enhancements in both mental health literacy and overall well-being among the students. This program could potentially address the growing worldwide demand for universally accessible digital services, particularly in response to the lockdowns resulting from the COVID-19 pandemic. |
| Kushner et al. 2011 | Using Behaviour Change Plans to Improve Medical Student Self-Care | Non-Randomised Control Trial | 10.5 | 14 | Study aims to determine the effectiveness of behavioural change planes in improving medical student self-care. | A group of 343 second-year medical students from North-western University Feinberg School of Medicine participated in a mandatory Healthy Living unit. They engaged in a Behaviour Change Project (BCP), which involved selecting a personal behaviour to modify, such as exercise, nutrition, sleep, personal habits/hygiene, study/work habits, or mental/emotional health. They then set goals, tracked their progress, and self-assessed their success in achieving these goals. Using a one-group post-test-only design, the authors of the study conducted both quantitative and qualitative assessments of the students' BCPs and their attitudes towards the project. | 139 students successfully met their set goals, 170 did not attain them, and 34 were uncertain about their outcomes. Utilizing factor analysis, two distinct attitude scales were identified: utility and burden. Additionally, statistically significant success was observed primarily in the sleep behaviour category and associated with the utility attitude scale. Qualitative case reports were employed to offer further insights into the BCP targets, their management, and achieved outcomes. Prior to completing the assignment, a considerable 79.9% of students perceived themselves as healthier while 81.9% expressed their intention to use this process again in the future. | Engaging in a Behaviour Change Project (BCP) proves to be a valuable and efficient endeavour, providing medical students with a platform to put into practice various strategies and skills while also gaining insight into the challenges associated with altering health behaviours. |
| Lampe et al 2021 | Mindfulness-based intervention helps preclinical medical students to contain stress, maintain mindfulness and improve academic success | Non-Randomised Control Trial | 14.5 | NA | Study aimed to determine the interconnectedness between stress, mindfulness and academic performance. It also aimed to determine the effectiveness of a mindfulness-based intervention at reducing stress and improving academic achievements. | 143 medical students in their preclinical years were included in the studies. 41 participated in the intervention consisting of 6 two-hour sessions.86 students served as control. Perceived stress and mindfulness were assessed using the PSS-10 and the MAS respectively. Academic performance/success was assessed by results attained during the 6 exams occurring during the observation period. | Stress displayed an inverse correlation with mindfulness and with the outcomes of the most demanding examination. The implementation of mindfulness-based stress reduction intervention aided in mitigating stress and sustaining mindfulness over the observation period, and this influence endured for at least six months following the intervention's conclusion. In contrast, positive effects on academic achievement were temporary and only discernible upon completing the intervention | The positive outcomes observed in the short and immediate term due to six distinct interventions in mindfulness-based stress reduction are promising. These results emphasize the need for exploring different approaches to achieve lasting effects. |
| Lavadera et al. 2020 | MAP Train My Brain: Meditation Combined with Aerobic Exercise Reduces Stress and Rumination While Enhancing Quality of Life in Medical Students | Non-Randomised Control Trial | 12.5 | NA | Study aims to determine the effectiveness of MAP (a meditation combined with exercise) intervention on reducing stress and ruminations while enhancing quality of life in medical students. | Participants included first and second year medical students. The weekly sessions started with 30 mins of instruction training followed by 30 mins of silent meditation and ending with 30 mins of aerobic exercise. Outcome assessment including questionnaires of depressive symptoms, perceived stress, ruminative thoughts and quality of life pre and post interventions approximately 9 weeks apart. | After the interventions, participants showed significant reductions in ruminations in contrast to their non participating counterparts. The intervention group also reported an improved quality of life and reduced perceived stress. An overwhelming majority of students expressed their willingness to recommend these practices to their future patients. | In summary, Mindfulness Awareness Practices (MAP) training provides a practical and scientifically supported approach for medical students to sustain both their mental and physical well-being throughout their medical education and beyond. This approach also extends to benefiting their future patients. |
| Aherne et al.  2021 | Mindfulness based stress reduction for medical students: optimising student satisfaction and engagement | Quasi-Experimental Study | NA | 23 | Study aims to determine how first year (mandatory) and second year (voluntary) students in a graduate entry medical school perceive and rate their satisfaction with a mindfulness-based stress reduction (MSBR) program. | A mixed-method pre and post study was conducted involving Year 1 and Year 2) medical students who participated in a 7-week (MBSR) course. The study aimed to compare student satisfaction ratings before and after the course. Additionally, thematic analysis was employed to analyse the feedback provided by the students regarding their perception of the course. | The year 1 students who had mandatory attendance expressed lower satisfaction levels compared to the year 2 students who had optional attendance. Thematic analysis of the feedback provided by Year 1 students revealed themes such as appreciating the concept of the course but finding its execution lacking, and a desire for more practice and less discussion. On the other hand, Year 2 students' feedback themes included the session environment and their contentment with the tutors. | When the MBSR course was offered on an optional basis, it garnered significant levels of satisfaction and received positive feedback. Key to the success of a self-care program like this is tailoring it to meet the specific needs of each participant and fostering a secure and welcoming environment. |
| MacLaughlin et al. 2011 | Stress Biomarkers in Medical Students Participating in a Mind Body Medicine Skills Program | Non-Randomised Control Trial | 16 | NA | Study aims to determine the effectiveness of a Mind Body Medicine Skills Course at reducing stress in medical students. | To achieve the aim, saliva samples was collected at different time points: before the intervention, and after the completion of the MBMS, as well as from controls. Notably, the collections at T2p and T2c were conducted during the period of final examinations. The measured physiological parameters included cortisol, dehydroepiandrosterone-sulphate (DHEA-S), testosterone, and secretory immunoglobulin A (sIgA). | The average morning salivary cortisol levels at T2p were 97% of those recorded at baseline T1. This reduction was significantly more prominent compared to T2c. Similarly, the pattern for DHEA-S closely followed that of cortisol, with T2p levels being markedly lower than T2c levels (P < .001) for both morning and evening collections. The testosterone ratio at T2p (0.85) was also notably lesser than T2c (1.6). However, there were no statistically significant differences observed in sIgA levels. Upon direct comparison, the mean values for cortisol, DHEA-S, and testosterone were significantly distinct between T2c and T2p | Throughout the academic semester, participants in the intervention managed to keep their hormonal levels within the normal range. In contrast, the control experienced notable increases in hormonal levels, likely intensified by the stress associated with end-of-semester exams. |
| Bermudez et al. 2020 | Improved quality of life and reduced depressive symptoms in medical students after a single-session intervention | Quasi-Experimental Study | 13.5 | NA | Study aims to determine the effectiveness of a The Unified Protocol for Transdiagnostic Treatment of Emotional Disorders (UP), a cognitive-behavioural treatment protocol for neuroticism on psychiatric symptoms and the quality of life of medical students. | The participants medical students aged 18 and above who did not exhibit psychotic symptoms, severe depressive episodes, or acute psychiatric risk. These participants underwent a psychiatric clinical interview using the Mini-International Neuropsychiatric Interview (MINI). Assessments were conducted at the beginning (baseline), and then at 7 and 30 days following a single-session Unified Protocol (UP) intervention. This intervention encompassed elements like experimental avoidance, quality of life, self-esteem, empathy, and anxiety symptom scales. Additionally, a subsequent evaluation was carried out 90 days post-intervention. | A total of sixty-two students took part in the study. After ninety days from the intervention, noteworthy changes were observed. There were notable decreases in the number of students meeting the criteria for social anxiety disorder (p = 0.013) and panic disorder (p = 0.001). Furthermore, there were significant enhancements in various areas, including reductions in depressive symptoms and improvements in quality of life. | The UP protocol reduces anxiety and depressive symptoms in medical students. The utilisation of a single session group format could potentially lower cost and enhance its practicality. However, to establish the credibility of these results, future studies employing placebo-controlled designs are imperative. |
| Bond et al 2013 | Embodied health: the effects of a mind-body course for medical students | Quasi-Experimental Study | 16 | NA | The objective of this study was to assess the psychological impact of an elective course called "Embodied Health" (EH) on medical students. This course spanned 11 weeks and integrated practices like yoga and meditation with neuroscience education. | The study examined the impact of an 11-week elective course called "Embodied Health" on 27 first- and second-year medical students. The effects were assessed through surveys covering four aspects: empathy, perceived stress, self-regulation, and self-compassion. The following scales were employed for measurement: Jefferson Scale of Physician Empathy, Cohen’s Perceived Stress Scale, Self-Regulation Questionnaire, Self-Compassion Scale: Additionally, students expressed the impact of the Embodied Health course on their well-being through post-course essays. | There was an increase in self-regulation and self-compassion. As well as an increase in empathy and a decrease in perceived stress. The qualitative analysis of students' essays revealed recurring themes, which aligned with the quantitative effects of the Embodied Health course: Reconnection between mind and body. Building a sense of community within a competitive environment. Heightened mindfulness. Increased confidence in applying mind-body skills with patients. Enhanced stress management. | The mind-body course designed for medical students led to improvements in self-regulation and self-compassion. These enhancements were consistent with the qualitative themes that emerged from students' post-course essays, indicating a strong alignment between the observed effects and the students' reflections. |
| Brennan et al. 2016 | A Stress Management Program for Higher Risk Medical Students: Preliminary Findings | Quasi-Experimental Study | 16 | NA | Study aimed to determine the effectiveness of a stress management intervention on anxiety, depression and self-efficacy in first year medicals students. | Forty-two medical students volunteered to participate. An eight-session intervention was provided, concentrating on cultivating relaxation skills, adaptive coping mechanisms, and fundamental nutritional knowledge. Prior to and after the intervention, levels of anxiety, depression, and self-efficacy were evaluated. Interestingly, this particular group of students exhibited notably higher initial levels of depression and anxiety but lower levels of self-efficacy in comparison to a previous study involving medical students from the same institution (p < 0.03). | Following the intervention, notable improvements were statistically significant for anxiety (p < 0.05) and self-efficacy (p < 0.05). However, there was no statistically significant improvement observed for depression. | The initial levels of anxiety and depression among this group of students indicated that they were potentially vulnerable to developing clinical syndromes in the future. The intervention focused on mitigating the impact of stress and was linked to positive changes in markers of distress. This suggests that such interventions could potentially modify the long-term risk of developing clinical conditions. |
| Chung et al. 2018 | A Targeted Mindfulness Curriculum for Medical Students During Their Emergency Medicine Clerkship Experience | Quasi-Experimental Study | 15.5 | NA | Study aimed to determine the effectiveness of an innovative mindfulness-based curriculum designed for EM clerkship on managing stress and reducing the risk of burnout in senior medical students. | The curriculum encompassed the following elements:  1. Classroom Sessions: Four weekly sessions, each lasting 60 minutes, were conducted.  2. Prerequisite Reading: Participants were required to complete specific reading assignments prior to the sessions.  3. Individual Practice: Students engaged in daily meditation practice and maintained personal journals.  4.Wellness Plan Development: Participants worked with a mentor to create a personalized wellness plan. | A total of thirty students took part in the curriculum, with twenty of them being included in the final analysis. Each student completed surveys before, immediately after, and six months after engaging in the curriculum. The study identified noteworthy alterations in self-reported behaviours and attitudes among the students immediately after their involvement in the curriculum. These changes were observed to persist up to six months following the completion of the curriculum. | Although this was a pilot study, the intervention yielded a sustained self-reported behavioural impact on students. This intervention can be adapted to mitigate burnout and increase wellness in medical school. |
| Damiao et al. 2020 | Effects of a Required Large-Group Mindfulness Meditation Course on First-Year Medical Students’ Mental Health and Quality of Life: a Randomized Controlled Trial | Randomised Control Trial | 17 | NA | The aim of the study was to determine the effectiveness of a mandatory large group mindfulness meditation course on first year medical student’s mental health and quality of life. | The participants consisted of first year medical students who were randomised into two groups, an intervention and a control The intervention group received the 6-week mindfulness protocol. Outcome assessment includes the use of life (WHOQoL-Bref), stress, anxiety and depression (DASS 21) and the facets of mindfulness (FFMQ) at the start and at the end of the interventions. | 141 students were included with 70 in the intervention, and 71 in controls. The intervention group did not exhibit any significant improvements in mental health indicators, quality of life or FFMQ scores when compared to the control group. | Implementing a mandatory mindfulness course for large cohorts during the first semester of medial school does not demonstrate any significant enhancement in mental health and quality of life of medical students. |
| Danilewitz et al. 2018 | Feasibility and effectiveness of an online mindfulness meditation program for medical students | Quasi-Experimental Study | 16 | NA | The aim of this study was to determine the feasibility and effectiveness of an online mindfulness intervention for medical student wellness. | Participants consisted of 52 medical students. The feasibility of the study was determined by the ease of recruitment, number of modules completed, satisfaction with the program and adherence to a regular meditation practice. Outcome assessment consisted of the Maslach Burnout Inventory, the Jefferson Scale of Empathy- medical student version, the Five Face of Mindfulness Questionnaire-short form, and the Self Compassion Scale- short form which were completed before and after the intervention. | On average, participants completed approximately 4.85 modules (±2.7). The mean satisfaction level with the modules was rated at 7.07 out of 10 (±1.1). However, adherence to a consistent formal meditation practice was low, with an average meditation time of 34.14 minutes (±27.44) per module. Notably, self-compassion and the facets related to the practice of "observe and describe" in mindfulness demonstrated statistically significant increases from baseline. However, no notable changes were observed in levels of burnout and empathy. | The findings of this study suggest that medical students are interested in an online mindfulness meditation program. However, the results did not offer substantial evidence of the program's effectiveness. Despite this, we believe that further research and development are necessary to investigate its potential benefits and efficacy more comprehensively. |
| Dyrbye et al 2017 | The Impact of a Required Longitudinal Stress Management and Resilience Training Course for First-Year Medical Students | Quasi-Experimental Study | 15.5 | NA | This study aimed to determine the effectiveness of a mindfulness-based stress management intervention in reducing psychological distress in medical student. | The study consisted of two groups of medical students who took part in a compulsory stress management and resilience course. These students completed questionnaires both before and after the course. | The paired analysis of individual students revealed a decline in mental QOL, happiness and empathy along with an increase in stress courses over the span of a year. However, no significant changes were noted in burnout or resilience. Both cognitive and emotive empathy demonstrated a decrease in both cohorts. However, this decline reached statical significance in cohort 1. | The mandatory longitudinal mindfulness-based stress management course conducted among first-year medical students did not result in noticeable enhancements in the well-being or empathy of the medical students. These outcomes differ from studies involving voluntary medical students or physicians, where a similar curriculum showed potential in reducing burnout and stress. It is recommended that medical schools provide a range of effective options to students, allowing them to choose activities they are interested in participating in. |
| Erogul et al. 2014 | Abridged Mindfulness Intervention to Support Wellness in First-Year Medical Students | Randomised Control Trial | 17 | NA | This study aimed to determine the effectiveness of a 8 week mindfulness based stress reduction intervention on improving medical student wellness. | 58 participants were randomized to control and interventions groups. Outcome assessment measures includes the Perceived Stress Scale (PSS), the Resilience Scale (RS), and Self-Compassion Scale (SCS). These were measured at 3 different time points: at bassline, at conclusion, 6 months after conclusion. The intervention involves 75 minutes of weekly class times, suggested meditation at home and a half-day retreat in the last week. | The intervention group experience an increase in SCS scores which were significant both at the study’s conclusion and at the 6-month mark. In addition, the PSS scores significantly decreased at the study’s conclusion, although this reduction was not sustained at 6 months post-study. The study did not show any difference in RS. However, RS was found to have significant correlations between SCS and PSS scores. | The implementation of the intervention led to enhancements in perceived stress and self-compassion among first year medical students. This suggest that it could serve as a valuable component of the curriculum, contribution to both student wellness and professional development. |
| George et al. 2013 | Facebook-based stress management resources for first-year medical students: A multi-method evaluation | Randomised Control Trial | 17 | NA | This pilot study aimed to investigate the potential benefits of a stress management intervention conducted solely on Facebook. | During the orientation week, first year medical students were randomly divided into the intervention and control group. The intervention group focused on addressing challenging issues during the initial semester of medical school. The evaluation consisted of the use of descriptive statistics for demographics and frequencies and qualitative procedures for focus group data. | The convenience and user-friendliness of a stress management program based on Facebook were found to be beneficial for medical students, especially during the initial phase of the semester when participation and involvement were most pronounced. | Based on the initial findings, medical schools could explore the integration of an online networking element into their current stress management programs. The online approach might hold advantages for other healthcare professional and students across diverse health disciplines. |
| Gowda et al. 2018 | Art as Sanctuary: A Four-Year Mixed-Methods Evaluation of a Visual Art Course Addressing Uncertainty Through Reflection | Quasi-Experimental Study | NA | 22 | The study aimed to determine the effectiveness of a visual arts course in addressing uncertainty through reflection. | The course which was conducted over 4 years, consisted of a total of 47 students. Pre and post intervention, students filled in the following questionnaires: Groningen Reflection Ability Scale (GRAS) for reflective ability, the Tolerance for Ambiguity scale for ambiguity, and Best Intentions Questionnaire for personal bias awareness. The study also employed focus group interviews and narrative post-course evaluations as data collection methods. These data were then subjected to coding and thematic analysis to derive meaningful insights and patterns. | A statistically significant improvement was observed in GRAS scores. The qualitative analysis identified several themes, including the enhancement of students' observational skills, an increased awareness of the subjective and uncertain nature of perception, exploration of diverse perspectives, and the recognition of the course as a means of restoration and fostering connections with classmates. | Integrating visual art into medical education proves to be an efficient pedagogical approach to tackle core competencies crucial for training, such as observation, reflection, and self-care. |
| Greeson et al. 2015 | An Adapted, 4-Week Mind-Body Skills Group for Medical Students: Reducing Stress, Increasing Mindfulness, and Enhancing Self-Care | Quasi-Experimental Study | NA | 10 | This study aimed to assess the feasibility, acceptability and initial effectiveness of an adapted 4-week stress management and self-care workshop for medical students. | The study employed a prospective, observational, and mixed-methods approach, incorporating pre-test and post-test evaluations. The participant pool comprised medical and physician-scientist (MD/PhD) students from a prominent medical school in the south-eastern region. Feasibility was evaluated by examining workshop enrolment and completion rates. Acceptability was gauged through qualitative ratings and open-ended responses that solicited feedback on the perceived value of the workshop. Additionally, quantitative outcomes encompassed students' assessments of stress and mindfulness through validated self-report surveys. | Enrolment showed a progressive increase, with 6, 15, and 23 students attending the workshops in 2007, 2009, and 2011, respectively. 82% of students successfully completed the workshop, demonstrating the feasibility of the 4-session extracurricular format. Feedback was positive, with many students reporting that it equipped them to better manage the stress and emotional demands of medical school. Moreover, they noted an enhancement in self-care behaviours and decrease in perceived stress. These changes were significantly correlated, with stress and mindfulness changes. | Collectively, these outcomes indicate that a concise and optional mind-body skills workshop, tailored for medical students, is both attainable and well-received. Furthermore, the workshop has proven effective in decreasing stress levels, augmenting mindfulness, and promoting improved self-care behaviours among students. |
| Soulhaug et.al 2018 | Six-year positive effects of a mindfulness-  based intervention on mindfulness, coping and well-being in medical and psychology  students; Results from a randomized  controlled trial | Randomised Control Trial | 14.5 | NA | This study aimed to evaluate the longitudinal impact of a seven-week mindfulness-based course on medical and psychology students. | 288 Norwegian medical and psychology students took part in the trial. Among them 144 engaged in a 15-hour mindfulness course spanning seven weeks during their second or third semester, supplemented by a booster session twice a year. The remaining students were controls. The study assessed outcomes related to subjective well-being, dispositional mindfulness, and coping strategies using the Five Facet Mindfulness Questionnaire and the Ways of Coping Checklist. | After a six-year follow-up, students who had undergone mindfulness training reported higher levels of well-being. This was accompanied by enhancements in their levels of dispositional mindfulness and problem-focused coping. These positive effects persisted despite the fact that participants showed relatively low levels of adherence to formal mindfulness practice. | The results underscore the effectiveness of mindfulness training in enhancing well-being and fostering adaptive coping skills. This could have positive implications for the quality of care provided by healthcare professionals and contribute to their resilience and ability to persist in their roles. |
| Slavin et.al 2014 | Medical Student Mental Health 3.0: Improving  Student Wellness Through Curricular Changes | Quasi-Experimental Study | 8.5 | NA | This study aims to determine the effectiveness of curricular changes in improving wellness in medical school. | An integrated, comprehensive preclinical curriculum reform initiative was introduced through the Office of Curricular Affairs at the Saint Louis University School of Medicine, beginning in the academic year 2009-2010. At the start and end of the first academic year, students completed the following validated questionnaires: The Center for Epidemiological Studies Depression Scale, the Spielberger State-Trait Anxiety Inventory, the Perceived Stress Scale, and the Perceived  Cohesion Scale. In addition, we tracked students’ overall satisfaction with the wellness programs offered, using the Association of American Medical Colleges’ Graduation Questionnaire (GQ), | The study demonstrated that implementing significant and targeted changes to various aspects of the medical school curriculum, including course content, contact hours, scheduling, grading, electives, learning communities, and required resilience/mindfulness experiences, resulted in notable improvements. Students who participated in the expanded wellness program exhibited lower levels of depression symptoms, anxiety symptoms, and stress. Additionally, they reported higher levels of community cohesion compared to students who were enrolled before these changes were made. | This study highlights the significance and applicability of incorporating curricular changes into models aimed at enhancing medical students’ mental health. |
| Short et al. 2019 | An immersive orientation programme to improve medical student integration and well-being | Cross-Sectional  Study | 7 | NA | Beginning Education at Central Coast Hospitals (BEACCHES) is an orientation aimed at supporting students in the transition to regional clinical placements and promote student wellbeing through social cohesion. This study aims to gain insight into which aspects of the programs were perceived as the most valuable and impactful by participants, thus providing valuable information for refining and optimizing the program in the future. | The program was offered to medical students starting regional clinical placement at the Central Coast Medical School, Australia. The program places a strong emphasis on teamwork and self-care while fostering multidirectional interactions among peers, staff, and the local community. The evaluation of the program was conducted using semi-structured interventions mixed-method analysis of quantitative data (pre- and post-programme knowledge and confidence scores) and qualitative data (emergent themes). | There was an improvement in knowledge acquisition in all domains.­ This includes items such as psychological first aid, student support, connectedness with fellow students and staff and self-management of health and workload. These items could also aid in improving wellbeing. Furthermore, qualitative analysis indicated strong endorsement for the significance of interactions with peers and staff outside of the formal educational context. | The survey results underscore the potential of the BEACCHES program to promote student well-being by offering focused orientation and engagement strategies that enhance a sense of connectedness. The program's emphasis on interactive and experiential learning proves valuable for students who are embarking on new regional clinical placements. |
| Shiralkar et.al  2013 | A Systematic Review of Stress-Management Programs for Medical Students | Systematic Review | NA | NA | This study aims to identify all the controlled trials of stress management interventions for medical students and determine their effectiveness. | The authors conducted a search for articles in the English language using databases like PsycINFO and PubMed. They used a combination of search terms such as "stress-management," "distress," "burnout," "coping," "medical student," and "wellness." The focus was on identifying both randomized controlled trials and controlled non-randomized trials of stress-management programs. | 13 studies were identified. The intervention consisted a range of approaches such as self-hypnosis, meditation, mindfulness-based stress reduction, providing feedback on health habits, educational discussions, changes in curriculum length and type, and alterations in the grading system. Interventions that demonstrated effectiveness in reducing stress and anxiety among medical students included mindfulness-based stress reduction, meditation techniques, self-hypnosis, and adopting a pass/fail grading system. | There are notable prospects for advancing research in this field by creating a greater number of high-quality studies. Emphasis should be placed on refining randomization techniques and establishing standardized outcome measures. |
| Sharif et al. 2017 | The Relationship between Religious Coping and Self-Care Behaviours in Iranian Medical Students | Cross-sectional  Study | 11.5 | NA | The aim of the study was to determine the correlation between religious coping methods and self-care behaviours in Iranian medical students. | The participants consist of 335 randomly selected medical students. Assessment was completed using a collection tool consisting of the standard questionnaire of religious coping methods and self-care behaviours. A two sample T test was employed using equal variances. Additionally, adjusted linear regression was utilized to assess the independent relationship between religious coping strategies and self-care. | The adjusted linear regression analysis revealed a statistically significant and independent association between positive religious coping and self-care behaviour. | The results show a positive correlation between religious coping and self-care behaviour. More research with greater sample sizes and different populations is recommended. |
| Shapiro et.al 2019 | Mindfulness Meditation for Medical Students: a Student-Led Initiative to Expose Medical Students to Mindfulness Practices | Randomised Control trial | 13 | NA | This study aimed to determine the feasibility and potential impact of a short mindfulness training program for medical students. It also aimed to gain insights into the perceptions, expectations and challenges related to practicing mindfulness. | 41 medical students were randomly assigned to two groups. An introductory mindfulness class or an introductory mindfulness class followed by an 8-week mindfulness meditation course. Both qualitative and quantitative data was collected pre and post intervention. The qualitative data was closed analysed as part of a grounded theory guided content analysis, creating a list of codes which were consolidated into overarching themes. | Mindfulness and awareness were found to be inversely correlated with levels of stress and depression. Those who participated in the full mindfulness course demonstrated greater familiarity with and willingness to use mindfulness techniques. However, the group that took only the introductory mindfulness class demonstrated no significant difference in terms of wellness outcomes. Qualitative data revealed that the students were open to incorporating mindfulness as an alternative approach in medicine and medical education. They also acknowledged the challenges associated with implementing such an alternative in the medical field. | The study concluded that a mindfulness course designed for medical students is both feasible and holds potential as a wellness and educational intervention. The findings suggested that even shorter-duration mindfulness interventions can provide benefits, making mindfulness more accessible to students. |
| Shapiro et al. 2018 | Medical Students’ Efforts to Integrate and/or Reclaim Authentic Identity: Insights from a Mask-Making Exercise | Cross-sectional  Study | NA | 15 | This study aimed at evaluating the experience of a mask making workshop during a wellness retreat for medical students. | A portion of first- and second-year medical students who took part in a wellness retreat organized by a medical school participated in a workshop focused on creating masks. Collaborative teams consisting of faculty and students analysed the masks and accompanying narratives through visual and textual analysis methods. Additionally, a quantitative survey was used to gauge student perceptions of the entire experience. | The following overarching theme was identified: " “Reconciliation/reclamation of authentic identity.” The integration of nonverbal mask-making and narrative techniques provides a comprehensive understanding of medical students' experiences and thoughts. | This workshop encouraged introspection and self-care, all the while offering valuable insights into both personal and professional growth. |
| Shapiro et al. 1998 | Effects of Mindfulness-Based Stress Reduction on Medical and Premedical Students | Randomised control trial | 13 | NA | This study aimed at examined the effects of a meditation-based stress reduction on premedical and medical students. | Participants were split into two groups, an intervention and control. The intervention consisted of a 7-week mindfulness-based intervention. Participants in both groups were measured pre and post intervention. The following scales were employed: Empathy Construct Rating Scale (ECRS), The Hopkins Symptom Checklist 90 (Revised) SCL-90-R, Subscale 4 of the SCL-90, The State-Trait Anxiety Inventory (Form Y) STAI Form 1, The Index of Core Spiritual Experiences INSPIRIT. | The results suggest that engaging in the intervention leads to: (1) a decrease in self-reported state and trait anxiety, (2) a decrease in overall psychological distress, including depression, (3) an increase in overall empathy levels, and (4) an increase in scores on a measure of spiritual experiences at the end of the intervention. Furthermore, these findings were observed: (5) in both the intervention and wait-list control groups, and (6) consistently across different experiments. | The study highlights the potential effectiveness of mindfulness training in improving the psychological and spiritual well-being of premedical and medical students. Additionally, it suggests that mindfulness training can help develop skills that these students can apply in their future roles as physicians. |
| Shapiro et.al 2000 | Stress Management in Medical Education: A Review of the Literature | Literature Review | NA | NA | To conduct a systematic review of clinical studies that offer empirical data on stress-management programs within medical training. | The researchers conducted a comprehensive search on Medline and PSYCHINFO databases, covering the period from 1966 to 1999. They focused on identifying studies that assessed stress management programs specifically designed for medical trainees, including medical students, interns, and residents. Inclusion criteria required studies to provide empirical data and have been carried out in allopathic medical schools. | The findings of the review indicated that stress-management programs for medical trainees yielded several positive outcomes. These included improvements in immunologic functioning, reductions in depression and anxiety levels, increased levels of spirituality and empathy, enhanced knowledge about alternative therapies for future referrals, improved understanding of stress effects, increased utilization of positive coping strategies, and improved ability to address role conflicts. | Future research in this area should take into account the following considerations to enhance the quality and validity of findings: “(1) rigorous study design, including randomization and control (comparison) groups, (2) measurement of moderator variables to determine which intervention works best for whom, (3) specificity of outcome measures, and (4) follow-up assessment, including effectiveness of future patient care.” |
| Saunder et.al 2007 | Promoting self-awareness and reflection through an experiential Mind-Body Skills course for first year medical students | Quasi-Experimental Study | NA | 10 | The aims of the study were to assess the influence of a 11-week mind-body skills course on student’s self-awareness, self-reflection as self-care as part of their medical education experience. | This research employs a qualitative content analysis methodology for analysing data. The dataset consists of 492 direct verbatim responses collected from 82 students in response to six open-ended questions. These questions were designed to explore students' experiences and perspectives following their participation in a mind-body skills course. The questions specifically inquired about the students' attitudes towards mind-body medicine, complementary medicine, and how they envisioned incorporating these approaches into their future roles as physicians. | The analysis of the data uncovered five core themes that emerged consistently in the students' responses: connections, self-discovery, stress relief, learning, and the role of these aspects in medical education. | Mind-body skills groups offer a hands-on method for teaching techniques that allow students to develop self-awareness and self-reflection, enabling them to practice self-care and gain familiarity with mind-body medicine during their medical education. |
| Sekhar et al.  2021 | Mindfulness-based psychological interventions for improving mental well-being in medical students and junior doctors (Review) | Systematic Review and Meta-analysis | NA | NA | The aim of the study was to evaluate the effects of psychological interventions with a main focus on mindfulness on the mental wellbeing and academic performance of medical students and junior doctors. | The search was conducted across various databases including the Cochrane Central Register of Controlled Trials (CENTRAL), MEDLINE, Embase, and additional sources, up until October 2021. The selection criteria included randomized controlled trials involving medical students at any stage and junior doctors in postgraduate years one, two, or three. The interventions considered were psychological in nature and focused primarily on teaching mindfulness as a preventive measure. Anxiety and depression were the primary outcomes of interest, with secondary outcomes encompassing stress, burnout, academic performance, suicidal ideation, and quality of life. The review process followed established Cochrane guidelines, including the use of Cochrane's risk of bias 2 tool (RoB2). | 10 studies involving 731 participants were included. Compared to controls, mindfulness intervention s did not result in a significant different immediately post intervention for anxiety. The change in the DASS-21 was also not clinically significant. There was also no significant improves for depression post intervention.  The improves found in secondary outcomes were found not to be clinically important. The secondary outcomes included stress, burnout, academic performance and quality of life. | The efficacy of mindfulness interventions in our specific target group remains uncertain. Limited research has been conducted on mindfulness interventions for junior doctors and medical students, and the existing studies are relatively small in scale with potential risk of bias concerns. Consequently, the current body of evidence is insufficient to draw definitive conclusions regarding the impact of mindfulness interventions in this population. Additionally, there is a lack of evidence to assess the potential long-term effects of mindfulness interventions. |
| Sankoh et al. 2019 | Mindfulness in Medicine: Modified Mindfulness-Based Stress Reduction (MBSR) Program among Future Doctors | Non-randomised control trial | 13 | NA | The aim of the study was to evaluate the impact of an 8-week MSBR course on student burnout and social cognition. | Participants were divided into three groups: 1. An 8-week mindfulness education course, adapted from Kabat-Zinn's Mindfulness-Based Stress Reduction (MBSR) program.  2. An 8-week general stress reduction course created by the researcher.  3. A no-treatment control group that eventually received the mindfulness-based and general stress reduction courses after the study.”  Post intervention, all participants completed self-report assessments on mindfulness, a student burnout inventory to measure stress-related outcomes, a test to evaluate their affective theory of mind, and an emotional intelligence test to assess their social-emotional abilities. | Modified-MBSR intervention did not result in significantly higher levels of mindfulness compared to either a general stress reduction course or no intervention. However, there is evidence supporting the positive impact of mindfulness on student burnout. Students who participated in the mindfulness intervention reported less exhaustion related to their schoolwork. When the control groups were combined and compared to the mindfulness intervention, significant differences were observed in exhaustion and personal efficacy. Moreover, a significant correlation between mindfulness and student burnout was identified, irrespective of group assignment. | The study's results align with existing research demonstrating the effectiveness of MBSR in reducing self-reported stress among healthcare trainees. Students who underwent the mindfulness intervention reported lower levels of student burnout compared to both the active and inactive control groups. However, the mindfulness program did not lead to notably higher levels of mindfulness or emotional intelligence in its participants compared to the control group participants. |
| Maclean et al. 2020 | A pilot study of a longitudinal mindfulness curriculum in undergraduate medical education | Non-randomised control trial | 12 | NA | The aim of the study was to determine the effectiveness of a longitudinal mindfulness curriculum in undergraduate medial education. | Medical students were engaged in an online questionnaire assessment that covered various aspects such as mindfulness (measured by the Freiburg Mindfulness Inventory), empathy (evaluated using the Jefferson Scale of Physician Empathy), resilience (measured through the Connor-Davidson Resilience Scale), and perceived stress (assessed using the Perceived Stress Scale). Additionally, the students' demographic information, home practice, and personal experiences were collected through surveys. These assessments were conducted at the curriculum launch and annually for a span of three years. | Although the overall scores on the assessment scales did not show significant changes after implementing the curriculum, a noteworthy increase was observed in mindfulness, empathy and resilience scores. Furthermore, participants who reported engaging in about two hours of home practice per week experienced statistically significant improvements, including a 14% increase in mindfulness scores, a 6% increase in empathy scores, a 10% increase in resilience scores), and an 11% decrease in stress scores. Despite receiving positive feedback for both mandatory and elective sessions, elective session attendance was notably low among students. | Incorporating a mindfulness curriculum into the formal undergraduate medical education is a workable endeavour. The advantages of such a curriculum might primarily apply to students who actively implement the principles of the curriculum and engage in regular practice. However, additional research is necessary to delve deeper into these effects and their broader implications. |
| Malpass et al. 2019 | Medical Students’ Experience of Mindfulness Training in the UK: Well-Being, Coping Reserve, and Professional Development | Quasi-Experimental Study | NA | 26 | The aim of the study was to evaluate the experience of an 8-week mindfulness training course for medical students. | Students who took part in the program were asked to complete a survey at the conclusion of the course, providing their thoughts and feedback in free-text format. Furthermore, six in-depth qualitative interviews were conducted, lasting from 60 to 90 minutes each. These interviews followed a predetermined topic guide and were recorded and transcribed word-for-word. The data analysis process involved using the framework approach to organize and analyse the collected information. | Students felt that mindfulness training brought about a transformed perspective towards their thoughts and emotions, resulting in an enhanced sense of control and resilience. They reported being better equipped to handle their academic responsibilities, and they expressed increased acceptance of their own limitations as learners. The supportive small group setting played a crucial role in this experience. Students also emphasized that their empathy and communication skills had improved significantly as a result of cultivating inner awareness of their thoughts and feelings, recognizing their judgments, and fostering a keen sense of attentive observation. | The results of the study illustrate the potential for building resilience and coping mechanisms within medical education, highlighting the significant role of mindfulness in this developmental process. The researchers propose a conceptual model that outlines a learned cycle involving specific vulnerabilities. They describe how Mindfulness-Based Cognitive Therapy (MBCT) intervenes at different points in this self-reinforcing cycle by fostering the acquisition of novel coping strategies that embrace a concept of "allowed vulnerability." |
| Manning-Giest et al 2020 | Pre-clinical Stress Management Workshops Increase Medical Students’ Knowledge and Self-awareness of Coping with Stress | Non-randomised Control trial | 15.5 | NA | The aim of this study was to examine the impact of a stress management workshop on the knowledge of stress and potential coping strategies among medical students. | A panel discussion followed by small group sessions focusing on stress in clinical medicine, learning challenges, competition, managing stressful events, and recognizing burnout symptoms was conducted for medical students entering clerkships. The study utilized a longitudinal survey design to evaluate changes in knowledge, confidence, skills, and attitudes related to stress and coping strategies among participating students (N = 135) before, immediately after, and three months after the intervention. Paired t-tests and multivariate analyses were employed to compare survey responses using a 5-point Likert scale. | The survey response rates were 90.4% for the pre-workshop assessment, 77% for the post-workshop assessment, and 71.1% for the long-term post-workshop assessment. Comparing the results, students showed significant improvement across all four domains immediately after the workshop: knowledge (4.4 vs. 4.7, p < 0.05), confidence (3.6 vs. 3.9, p < 0.05), perceived skills (3.3 vs. 3.7, p < 0.05), and attitude (2.6 vs. 2.8, p < 0.05). While scores slightly decreased after three months compared to immediate post-workshop, they remained significantly higher than the pre-workshop scores. | Participating in a stress management workshop can enhance medical students' understanding of the effects of stress and their ability to employ effective strategies for coping with stress. |
| McGrady et al. 2012 | A Wellness Program for First Year Medical Students | Randomised Control Trial | 16.5 | NA | The aim of this study was to evaluate the effectiveness of a structured wellness program on measures of anxiety, depression and frequency of acute illness in first year medical students. | 449 first year medical students participated and were divided into an intervention and control group. The intervention consisted of 8 structured wellness and stress management sessions. Students who were considered at high risk were identified through their scores on psychological assessment tools and the number of recent life events (WLE) they had experienced. | Depression and anxiety scores, as well as the frequency of acute illness, were higher in females. Additionally, students who had experienced multiple recent life events (WLE) also exhibited higher levels of depression, anxiety, and acute illness. When accounting for the number of life events (WLE) as a factor, the intervention group students showed significant reductions in depression scores. Moreover, the high-risk group consistently demonstrated lower depression scores following the intervention compared to high-risk students on the wait list control. These changes persisted throughout the school year. However, there were no significant alterations in anxiety scores or the frequency of acute illness. | Wellness programs have the potential to be introduced in medical schools and could prove especially beneficial for incoming students who are experiencing heightened psychological distress. |
| McGrady et al. 2019 | Effects of a Lifestyle Medicine Elective on Self-Care Behaviours in Preclinical Medical Students | Non-Randomised Control Trial | 14.5 | NA | The aim of the study was to evaluate the impact of a lifestyle medicine elective on medical student’s self-care behaviour. | The participants included voluntary first- and second-year students. The elective consisted of four group sessions, with two sessions held at the beginning and two at the end of the program. In the initial session, students were educated about the impact of lifestyle on both mental and physical health. They were asked to complete screening assessments to evaluate their own levels of physical activity, nutrition, anxiety, and depression. In the subsequent class, students were presented with their screening scores and were given the option to select one of three focus groups: nutrition, physical activity, or stress management. In their chosen area, they set specific goals. The concluding sessions of the elective were centred around patient cases. A total of 63 students participated in the elective and provided their data. | When comparing the baseline scores between genders, it was found that women had significantly higher scores on the depression screener and lower scores on the physical activity assessment compared to men. In terms of the entire group, a comparison of pre and post elective scores revealed significant improvements in nutrition, depression, and anxiety. Upon further examination, the stress management group exhibited significant improvements in their anxiety scores. In the nutrition group, there was a significant decrease in fat consumption and an increase in the intake of fruits and vegetables. However, the physical activity group did not experience a significant increase in daily physical activity, although this assessment was limited due to missing data and wide variability. | The course received positive evaluations from students. Medical students were able to enhance their own lifestyle behaviours and simultaneously gain valuable information that could be beneficial in their future patient care endeavours. |
| Moir et al. 2015 | Computer Assisted Learning for the Mind (CALM): the mental health of medical students and their use of a self-help website | Non-Randomised Control Trial | 15.5 | NA | The objective of this study was two-fold: first, to create an evidence-based self-help website called Computer Assisted Learning for the Mind (CALM) with the goal of enhancing mental health among medical students; and second, to evaluate the proportion, demographics, and mental health status of the students who opted to use the website. | The participants consisted of 2^nd^ and 3^rd^ year medical students from one New Zealand university. The study involved comparing the demographic characteristics and mental health scores of medical students who utilized the CALM self-help website with those who did not. Baseline depression (PHQ-9) and anxiety (GADS-7) scores were assessed as outcome measures. To maintain anonymity, unique identifiers were assigned to track the usage of the website by the participants. | A total of 279 out of 321 eligible students completed the baseline questionnaires. Among the participants, 80 out of 321 students accessed the CALM website during a 5-week period. Comparing those who accessed CALM with those who didn't and could be identified through unique identifiers (49 accessed, 230 didn't), it was found that those who accessed CALM had significantly higher baseline anxiety scores. Although depression scores were not significantly higher among CALM users, 41% of students who were at risk of significant depression and anxiety at baseline went on to access CALM. | A quarter of medical students utilized the CALM website, primarily those with higher levels of anxiety. This self-selection of students towards a web-based resource could potentially offer support to those with greater needs, although additional research is required to evaluate its overall effectiveness. |
| Moir et al. 2016 | A Peer-Support and Mindfulness Program to Improve the Mental Health of Medical Students | Randomised Control Trial | 16.5 | NA | The aim of this study was to determine the impact of a peer support and mindfulness program on the mental of medical students. | An exploratory study was carried out involving 2nd- and 3rd-year medical students at a New Zealand medical school. The participants were divided into two groups. The intervention group received a program that included peer support and mindfulness meditation sessions taught by fellow students who were trained as peer leaders. The control group had access to existing mental health resources but did not receive the additional intervention. The main focus was on measuring changes in depression (PHQ-9) and anxiety (GAD-7) scores. Secondary measurements included quality of life, resilience (15-item resilience scale), academic self-concept, and motivation to learn. These assessments were conducted at the beginning and 6 months after the intervention. | Out of the 402 eligible students, 275 chose to participate in the study, and 232 completed it. The baseline characteristics showed that 53% of participants were female, with a mean age of 21 years. Their initial scores were a mean of 5.2 on the PHQ-9 scale for depression and 4.5 on the GAD-7 scale for anxiety. 12 students were selected as peer leaders. There was a good level of engagement, with a quarter of intervention students utilizing face-to-face peer support, and over 50% attending a peer social event and/or engaging in the mindfulness program. Although improvements in mental health were observed in the intervention group, the difference between the intervention and non-intervention groups did not achieve statistical significance. | While peer support and mindfulness have shown effectiveness in various settings, this preliminary study did not find a statistically significant impact. Future research avenues could explore extending the training duration for peer leaders, focusing on individuals with more pronounced mental health challenges, utilizing more intensive interventions, or increasing the sample size. Employing a cluster randomized study design could also help mitigate the risk of intervention contamination and provide more robust results. |
| Sahranavard et al. 2019 | The effectiveness of group training of cognitive behavioural therapy‐based stress management on anxiety, hardiness and self‐efficacy in female medical students | Quasi-Experimental Study | 17 | NA | The aim of the study was to evaluate the effectiveness of cognitive behavioural therapies (CBTs) for anxiety, hardiness, and self‐efficacy in female students of Birjand University of Medical Sciences. | This study utilized an interventional approach, involving 30 participants who were selected through available sampling and then randomly assigned to two groups: an experimental group receiving Cognitive Behavioural Therapy (CBT) and a control group, each consisting of 15 female students. Data was collected using the Beck Depression Inventory questionnaire, as well as the Beck Anxiety Inventory, Ahvaz Hardiness Inventory, and Schwartz's General Self-Efficacy Scale. These questionnaires were administered pre-test and post-test. The experimental group underwent a 6-session protocol of cognitive behavioural group therapy, while the control group did not receive any intervention. | The intervention group exhibited a significant decrease in the mean levels of anxiety (p < 0.001), while the mean levels of hardiness and self-efficacy increased in the post-test assessment for the experimental group (p < 0.001). | The findings from this study suggest that Cognitive Behavioural Therapy (CBT) is an effective approach for improving levels of anxiety, hardiness, and self-efficacy. This implies that by addressing anxiety, it's possible to enhance levels of hardiness and self-efficacy among university students, enabling them to better handle various challenges in their lives. |
| Sanchez-Campos et al. 2020 | Mindfulness in medical education: student’s perceptions and four recommendations for implantation on a mindfulness intervention | Quasi-Experimental Study | NA | 26 | The aim of this study was to investigate student’s perceptions and recommendations of the implementation of a mindfulness intervention in medical school. | A compulsory three-hour workshop was provided to third-year medical students as part of their family medicine academic week. Eleven students agreed to participate in two interviews, where their perspectives on mindfulness, the workshop's impact on their personal and professional well-being, and their opinions regarding the potential implementation of a continuous mindfulness curriculum were explored. | Students reported experiencing positive changes in terms of stress reduction, enhanced self-awareness, and personal growth, which they believed positively influenced their patient care. However, some barriers were identified, including time constraints, forgetfulness in practicing mindfulness, and a lack of understanding about mindfulness itself. Institutional benefits included the promotion of student well-being and the positive reputation associated with supporting such initiatives. On the other hand, the major institutional barrier was the crowded curriculum that posed challenges to expanding the mindfulness program. | The incorporation of mindfulness into the medical school curriculum through the workshop yielded perceived advantages on personal, institutional, and professional levels. However, there is a requirement for further research to quantitatively assess these benefits more comprehensively and to devise strategies for addressing barriers that emerge at both the individual and institutional levels. |
| Roy et al. 2019 | Well-being workshop: simple acts of care | Quasi-Experimental Study | NA | 16 | To determine the impact of a practical interprofessional well-being workshop for first year medical and nursing students. | Across six afternoons, a total of 251 students participated in groups of 12–15, engaging in our interactive workshop that revolved around three key themes: self-care, empathy, and communication. After finishing the workshop, we gathered written feedback and take-home messages from the participants and subjected them to thematic analysis. | Participants provided positive feedback on the interprofessional nature of the workshop and the practical strategies they learned. The take-home messages emphasized the importance of integrating well-being into the educational curriculum, with a specific focus on self-care. Students recognized the significance of "simple acts of care" directed both towards themselves and others as crucial for enhancing and sustaining well-being. | The interprofessional well-being workshops, aimed at first-year nursing and medical students, were effective in facilitating open conversations about self-care, empathy, and communication. Participants found practical advice for well-being valuable. The workshops highlighted the importance of understanding and practicing "simple acts of care," which has the potential to positively influence individuals and the broader culture they contribute to. |
| Rosenzweig et al. 2003 | Mindfulness-Based Stress Reduction Lowers Psychological Distress in Medical Students | Non-Randomised Control Trial | 16 | NA | The aim of this study was to determine the effectiveness of a mindfulness-based stress reduction course in reducing psychological distress in medical students. | A group of second-year students (n = 140) voluntarily enrolled in a 10-week Mindfulness-Based Stress Reduction (MBSR) seminar, while another group (n = 162) participated in a didactic seminar on complementary medicine. The participants' mood states were assessed using the Profile of Mood States (POMS) before and after the intervention. | At the start of the study, the MBSR group had a higher baseline total mood disturbance (TMD) compared to the control group (38.7 ± 33.3 vs. 28.0 ± 31.2; p < .01). Despite this initial difference, the MBSR group showed a significant reduction in TMD scores at the end of the intervention period (31.8 ± 33.8 vs. 38.6 ± 32.8; p < .05). Additionally, there were significant improvements observed in the Tension–Anxiety, Confusion–Bewilderment, Fatigue–Inertia, and Vigor–Activity subscales for the MBSR group. | The results suggest that Mindfulness-Based Stress Reduction (MBSR) could potentially serve as an effective stress management intervention for medical students. |
| Rockfeld et al. 2020 | An Interactive Lifestyle Medicine Curriculum for Third-Year Medical Students to Promote Student and Patient Wellness | Quasi-Experimental Study | 13 | NA | To determine the impact and feedback of a interact lifestyle medicine curriculum for 3^rd^ year medical students. | A series of three hour-long workshops were designed for third-year medical students, utilizing interactive lectures, small-group discussions, and reflective activities. The workshops aimed to educate students about the principles of lifestyle medicine and enhance their ability to counsel patients on lifestyle changes and behaviour modification. The curriculum also aimed to promote student well-being. The effectiveness of the curriculum was evaluated through measures of student satisfaction with the sessions, self-perceived changes in knowledge, skills, and attitudes towards lifestyle medicine and behaviours change, both before and after the workshops. | Over a span of two years, a total of 183 students engaged in the workshop series. The sessions garnered strong positive feedback, with an average rating of 4.2 on a 5-point Likert scale. The curriculum had a notable impact, leading to a notable improvement in students' comprehension of the correlation between lifestyle elements and patients' health. Furthermore, their confidence in providing guidance for behavioural change through counselling was significantly boosted as a result of participating in the curriculum. | Lifestyle medicine offers a well-founded structure for educating students on the effects of lifestyle changes on chronic diseases. By equipping students with knowledge and competencies to enhance patient care in areas like stress management, nutrition, and physical activity, this curriculum empowers them to apply these skills in their medical practice. Additionally, the curriculum's emphasis on personal health promotion enables students to reflect on their own well-being, potentially serving as a protective factor against professional burnout. |
| Vajda et al.  2016 | "Peer2Peer" – A university program for knowledge transfer  and consultation in dealing with psychosocial crises in  med-school and medical career | Quasi-Experimental Study | 4.5 | NA | To determine the impact of "Peer2Peer" – A university program for knowledge transfer  and consultation in dealing with psychosocial crises in  med-school and medical career. | The "Peer2Peer" initiative offers a two-fold approach: it delves into the existing research on psychological stress and psychosocial challenges faced by students during high-stress situations, while also providing preventive strategies for managing such situations. This is accomplished through elective courses, lectures, and workshops. Moreover, "Peer2Peer" offers consultation services facilitated by student tutors who provide personalized guidance when needed. These tutors undergo continuous training to enhance their expertise in both organizational and professional aspects. | Starting from the summer term of 2015, a total of 119 students have undergone training through lectures and elective courses. As part of the program, 61 instances of brief consultations and 33 instances of comprehensive consultations have been supervised. Among these, two psychotherapeutic and one psychosocial follow-up sessions were advised. Additionally, there are seven students who actively engage as tutors within the program. | The "Peer2Peer" initiative aims to provide an easily accessible resource for medical students who are dealing with psychosocial crises and struggling with stress and academic challenges. The program has observed a positive trend in the number of support contacts, showing an increase from the summer term of 2015 to the winter term of 2015/16. The initial evaluation of the program's various components began in the winter semester of 2015/16. The student tutors involved in the program have not only gained practical skills in assisting students in crisis but have also developed organizational skills. |
| Inge van Dijk et al. 2015 | Mindfulness training for medical students in their clinical clerkships: two cross-sectional studies  exploring interest and participation | Randomised Control trial | 16 | NA | In this study, the primary focus is to analyse the level of interest and engagement among students who have begun their clinical clerkships. Additionally, the study aims to compare different groups of students based on their interest in mindfulness training and participation in a trial on the impact of Mindfulness-Based Stress Reduction (MBSR). The study assesses various factors such as psychological distress, personality traits, cognitive styles, and mindfulness skills. | Study 1, involved 4th-year medical students on their clinical clerkships. The study was a cross-sectional pilot that examined their interest in undergoing Mindfulness-Based Stress Reduction (MBSR) training. Comparisons were made using t-tests with Bonferroni correction to analyse scores on various assessments such as the Brief Symptom Inventory, Neo Five Factor Inventory, and Five Facet Mindfulness Questionnaire. The goal was to determine differences between students interested in MBSR and those who were not interested. Study 2, involved 4th-year medical students starting their clinical clerkships. They were invited to participate in a randomized controlled trial investigating the effectiveness of MBSR. This phase compared scores on the Brief Symptom Inventory, Irrational Beliefs Inventory, and Five Facet Mindfulness Questionnaire of participating students with those who chose not to participate. | In Study 1, among the 179 participating students, 95 students (53%) expressed interest in undergoing MBSR training. Comparing the two groups, interested students exhibited notably higher scores in psychological distress (p = .004) and neuroticism (p < .001) when contrasted with the 84 non-interested students. In Study 2, 72% participated in the randomized controlled trial investigating the effectiveness of MBSR. Among these participants, scores were significantly higher in psychological distress, worrying, and problem avoidance, and notably lower in mindfulness skills, as compared to the 41 non-participants. | Interest in mindfulness training and participation rates in a (RCT) evaluating the effectiveness of MBSR among clinical clerkship students were found to be similar (Study 1) or even higher (Study 2) than what has been observed in studies involving pre-clinical students. Notably, students who expressed interest in mindfulness training and those who participated in the RCT reported higher levels of psychological distress and traits related to psychopathology. Furthermore, participants in the RCT tended to score lower on mindfulness skills in comparison to their counterparts. |
| Inge van Dijk et al. 2016 | Effects of Mindfulness-Based Stress Reduction  on the Mental Health of Clinical Clerkship  Students: A Cluster-Randomized Controlled Trial | Cluster Randomised Control Trial | 16 | NA | The aim of the study was to determine the impact of mindfulness stress reduction training on the mental health of medical students during clinical clerkships. | The trial compared two groups: one receiving clerkship as usual and the other receiving clerkships along with additional MBSR. The intervention consisted of eight weekly two-hour sessions, which included educational teachings, meditation exercises, and group discussions. The participants completed online assessments at various time points: baseline, 3, 7, 12, 15, and 20 months. Validated tools were used to evaluate several outcomes including psychological distress, positive mental health, life satisfaction, physician empathy, mindfulness skills, and dysfunctional cognitions. | Out of 232 eligible students, 167 students participated in the study and were randomly assigned to either the MBSR group (n = 83) or the CAU group (n = 84) based on their clerkship group. The MBSR group experienced a small reduction in psychological distress. Dysfunctional cognitions also showed a slight reduction in the MBSR group. Positive mental health increased significantly in the MBSR group. Life satisfaction improved moderately in the MBSR group. Mindfulness skills also saw a moderate increase in the MBSR group. However, there was no significant effect observed on physician empathy (between the two groups during the 20-month follow-up period. | MBSR was both feasible and well-received by medical clerkship students. It led to a notable improvement compared to the control group over a 20-month period. |
| Van Vliet et al. 2017 | Long-term benefits by a mind–body medicine skills  course on perceived stress and empathy among  medical and nursing students | Quasi-Experimental Study | 14.5 | NA | The aim of the study was to determine if a mind-body medicine course could lead to a reduction in perceived stress and increase in empathy and self-reflection among medical and nursing students. | The study involved piloting a mind-body medicine skills course with medical and nursing students. The course included experiential sessions of mind-body techniques and group reflections. The main focus was on evaluating changes in perceived stress (measured by the Perceived Stress Scale), empathy (measured by the Interpersonal Reactivity Index subscales for perspective taking, fantasy, empathic concern, and personal distress), and self-reflection (measured by the Groningen Reflection Ability Scale). Questionnaires were administered at different time points: baseline, post-intervention, and at 6- and 12-month follow-ups. | The study involved 74 medical students and 47 nursing students. Results indicated that participating medical students experienced increased empathic concern, increased fantasy, and decreased personal distress compared to the control group over the 12-month follow-up period. Similarly, participating nursing students showed decreased levels of perceived stress and decreased personal distress compared to controls over the 12-month follow-up. | The study provided evidence of the sustained positive impacts of the Mind-Body Medicine (MBM) course on perceived stress and empathy among both medical and nursing students over the long term. |
| Waechtera et al. 2021 | Mitigating medical student stress and anxiety:  Should schools mandate participation in wellness  intervention programs? | Randomised Control Trial | 14 | NA | The aim of this study was to investigate the impact of assigning medical students to a wellness intervention. The focus was on evaluating the effects of this assignment on factors like adherence to and engagement in the intervention, as well as its potential influence on psychological and academic outcomes. | The 12-week intervention involved attending one-hour wellness sessions, with participants randomly assigned to one of three groups: (1) yoga, (2) mindfulness, or (3) walking. These sessions were held twice weekly. The participants' psychological well-being was assessed using standardized measures both before and after the intervention. | Students who were assigned to the intervention group demonstrated higher levels of engagement, spending more minutes participating in the assigned activities compared to the control group. Additionally, significant differences were observed in the changes between pre- and post-intervention measures. | The allocation of biweekly wellness intervention sessions has a beneficial effect on medical students by reducing their state anxiety and perceived stress, without any adverse effects on their academic performance. The students showed commitment to attending the sessions and expressed positive feelings about them after experiencing the sessions. The level of actual participation in the wellness activities appears to be more significant than the specific type of wellness activity chosen. |
| Warnecke et al. 2011 | A randomised controlled trial of the effects of mindfulness practice on medical student stress levels | Randomised Control Trial | 15 | NA | The aim of this study was to investigate if the practice of mindfulness decreases the level of stress suffered by senior medical students. | This study was a multicentre, single-blinded, randomized controlled trial conducted at three clinical schools. 66 medical students were randomly assigned to either an intervention group or a usual care control group using a block-randomization method. The intervention involved the use of an audio CD containing guided mindfulness practice. Intervention participants were instructed to use this daily over the course of 8 weeks. All participants completed self-report questionnaires at baseline and again at 8 weeks. Additionally, the intervention group completed a follow-up questionnaire at 16 weeks. The primary outcome measure assessed the change in scores on the Perceived Stress Scale (PSS) over time. The secondary outcome measure focused on changes in scores on the subscales of the Depression, Anxiety, and Stress Scale (DASS) over time. | At baseline, the participants in the study had mean scores of 15.7 on the (PSS) and 13.2 on the stress component of the (DASS). Both of these scores exceeded the scores typically observed in age-matched normative control data. The participants intervention group experienced significant reductions in their scores on the PSS (mean reduction of 3.44) and the anxiety component of the DASS (mean reduction of 2.82). There was also a borderline significant effect observed for the stress component of the DASS (mean reduction of 3.69). Furthermore, follow-up assessments at 8 weeks post-trial indicated that the positive effects of the intervention were maintained, suggesting the potential durability of the intervention's impact on stress and anxiety reduction | The study's findings suggest that mindfulness practice effectively reduced stress and anxiety levels in senior medical students. Given the prevalence of stress among medical students and its potential adverse effects on both student well-being and patient care, this study highlights the significance of a simple, self-administered, evidence-based intervention for managing stress in this at-risk population. It is recommended that such interventions be widely utilized to support the mental health and overall well-being of medical students |
| Weingartner et al. 2019 | Compassion cultivation training promotes  medical student wellness and enhanced  clinical care | Quasi-Experimental Study | 12.5 | 16 | The aim of this study was to investigate the impact and effectiveness of a compassion cultivation training course for medical students. | During a span of three years, a total of 45 students took part in the elective. The course involved using the Kentucky Inventory of Mindfulness Skills to evaluate aspects like observing, describing, acting with awareness, and accepting without judgment, both before and after the course. Qualitative analysis of students' self-reported experiences was also conducted to gain insight into their perceptions of compassion training and how they applied the skills they learned from the elective. | The results of the study indicated that certain cohorts of students who participated showed significant improvements in specific mindfulness skills, such as observing and accepting without judgment. Additionally, the qualitative analysis of students' feedback revealed that participants from all cohorts found the elective to be valuable and applicable. They reported using the mindfulness, meditation, and compassion skills they learned in various aspects of their lives, including handling stress related to personal, academic, and clinical responsibilities. | The findings from this pilot study suggest that compassion training has the potential to be a promising approach for addressing burnout among medical students. Not only does compassion training promote student wellness, but it also contributes to improved interactions with patients. |
| Williams et al. 2020 | Non-Randomised Control Trial | Promoting Resilience in Medicine: The  Effects of a Mind–Body Medicine Elective  to Improve Medical Student Well-being | 12.5 | 17 | The aim of this study was to determine the effectiveness of a mind-body medicine elective in improving medical student wellbeing. | A total of 24 first- and second-year medical students were enrolled in an 11-week elective program across three cohorts spanning two years. The students completed surveys anonymously to gather information about their experiences. The surveys included the Freiburg Mindfulness Inventory (FMI), the Perceived Stress Scale-10 item (PSS-10), as well as a set of multiple-choice and open-ended questions specifically designed for this study. | Among the students who had both pre- and post-test scores available, there was no significant change in perceived stress levels. However, the average scores on the Freiburg Mindfulness Inventory showed improvement. The vast majority of participants (95.8%) rated the course as "definitely" worth it. The most notable improvements were observed in the areas of mindfulness, relationships with peers, and the creation of a supportive environment within the medical school. Students highlighted that learning mindfulness and meditation skills, as well as increasing social support, played significant roles in positively impacting their overall well-being. | The findings suggest that offering a mind-body medicine elective course could be a practical approach to enhancing medical student well-being and their capacity to provide patient care. To assess the long-term effects of such courses, future studies should consider incorporating follow-up testing to determine if the observed benefits persist over time. Furthermore, more research is needed to fully understand the cost-effectiveness of providing the intervention to all medical students. |
| Yang et al. 2018 | Non-Randomised Control Trial | Happier Healers:  Randomized Controlled Trial of Mobile  Mindfulness for Stress Management | 13 | NA | The aim of this study was to determine if 10-20 minutes of daily meditation for 30 days using a mobile phone application could reduce perceived stress and improve wellbeing for medical students. | 88 medical students were randomised into intervention and control groups. The intervention group used the Headspace mobile application, which offers audio-guided mindfulness meditation, for a duration of 30 days. All participants completed assessments using the Perceived Stress Scale (PSS), Five-Facet Mindfulness Questionnaire (FFMQ), and General Well-Being Schedule (GWBS) at three time points: baseline (T1), 30 days (T2), and 60 days (T3). The study employed repeated measures analysis of variance (rANOVA) to compare the intervention and control groups across these time points, focusing on differences in stress levels, mindfulness, and overall well-being. | The study found a significant interaction between the treatment group and time for both perceived stress and general well-being. In the intervention group, there was a significant decrease in perceived stress from Additionally, the intervention group showed a significant increase in general well-being compared to the control group from baseline (T1) to the 30-day follow-up (T2), and this increase was maintained through the 60-day follow-up. | The findings suggest that incorporating a mobile audio-guided mindfulness meditation program can effectively reduce perceived stress among medical students. This has potential implications for their well-being and, by extension, the quality of patient care they provide. Integrating mindfulness training into medical school curricula could have far-reaching benefits, including addressing burnout, anxiety, and depression among physicians, as well as improving overall physician-patient interactions and outcomes. |
| Yogeswaran et al. 2021 | Systematic review | Effectiveness of online mindfulness  interventions on medical students’ mental  health: a systematic review | NA | NA | The aim of this study was to determine the feasibility and effectiveness of online mindfulness intervention in improving medical student’s mental health. | Various databases including ProQuest, Medline, PubMed, PsycINFO, Web of Science, IEEE Explore, Cochrane, and CINAHL were searched. The search terms employed were related to mindfulness, cognitive behavioural therapy, acceptance and commitment therapy, online interventions, web-based platforms, virtual approaches, internet-based resources, medical students, residency students, and residents. Only English-language articles published within the last ten years were considered for inclusion in the review. The focus was on identifying studies that discussed online interventions targeting medical students or residents. | Two studies that explored the effects of online mindfulness interventions on the mental health were identified. There is a paucity in research in this area, existing qualitative and quantitative evidence indicates positive outcomes, including improvements in self-compassion, perceived stress, cognitive skills utilization, mindfulness, development of coping strategies, and increased awareness of emotions and feelings. However, no evidence was found regarding the effectiveness of online mindfulness interventions on depression, anxiety, and burnout. Notably, the studies observed low program usage, and participation tended to decline as the interventions progressed towards completion. Highlighting the need for further research and development in this field. | The systematic review indicates that while there is potential for online mindfulness interventions to positively impact certain aspects of medical students' mental health, there is a lack of sufficient evidence to conclude their effectiveness in addressing burnout, depression, and anxiety. The review underscores the need for more rigorous research, particularly through longitudinal studies with randomized controlled trials, to establish a stronger and more reliable understanding of the impact of online mindfulness interventions on medical students' mental health outcomes. |
| Yusoff et al. 2013 | Randomised Control Trial | An Educational Strategy to Teaching Stress Management  Skills in Medical Education: the DEAL Model | 13 | NA | The aim of this paper was to obtain feedback on the DEAL mode: an approach to teaching stress management skills in medical education. This feedback will aid in gauging the effectiveness, relevancy, practicality and applicability of this mode. | 171 medical students who consented to take part were randomised into an intervention (n=88) and control (n=83) groups. Feedback was obtained from the intervention group at the end of the intervention and analysed using the SPSS version 18. | Approximately 85.23% of medical students, totalling 75 individuals, participated in the intervention. Over 80% of these students found the intervention valuable, pertinent, achievable, and suitable for learning stress management skills. The average perception score wasn't linked to factors like gender, years of study, and intervention sessions (p < 0.05), except for the race of the medical students, which showed an association. | In summary, the intervention developed using the DEAL model was highly regarded by medical students for its usefulness, relevance, applicability, and feasibility. The authors suggest that the DEAL model holds potential as an effective educational approach to improve the teaching of stress management skills in medical education. However, further research is needed to confirm its effectiveness, impacts, benefits, and applicability across different educational environments. |
| Yusoff et al. 2010 | Non-Randomised Control Trial | Impact of Medical Student Well-Being Workshop on The Medical Student’s Stress Level: A Preliminary Study | 12.5 | NA | The aim of this study was to evaluate the effect of a stress management program on student’s stress level. | The study utilized a quasi-experimental before-after comparison design, implementing a half-day stress-management program. Convenient sampling was employed, with a calculated sample size of 38 participants for this preliminary research. The 12-item General Health Questionnaire (GHQ-12) was administered before the program and again four months later. Data analysis was performed using SPSS version 12. | A total of 34 participants were part of this study. The research demonstrated a significant variance in participants' GHQ-12 scores before and after the program (p < 0.001). Additionally, it observed a noteworthy decrease in the percentage of distressed participants after completing the program (p < 0.05). | The study revealed that the program holds promise as a stress-management intervention, with evidence of a positive influence on the mental health of medical students through stress reduction. Furthermore, the program gained acceptance among medical students, suggesting that a similar approach might be valuable for implementation in other contexts. |
| Zúñiga et al. 2021 | Quasi-Experimental Study | Dispositional Mindfulness Reduces Burnout and Promotes Flourishing  in Medical Students: a Two‐Wave Latent Change Score Model | 12.5 | NA | The primary objective of this longitudinal study was to pinpoint elements that could decrease students' burnout and promote their overall well-being. This information would serve as a foundation for creating effective interventions. | In 2015 and again in 2017, a group of 1,117 medical students from eight universities in Chile participated in a longitudinal study. They were required to fill out established questionnaires that measured distress, burnout, positive mental health, academic engagement, and dispositional mindfulness. Through logistic regressions and a two-wave latent change score model, the researchers examined how these factors influenced burnout and flourishing (a state of optimal mental health), both in terms of predictive ability and their interrelation over the two-year period. | 639 students (57.2%) completed the questionnaires at both time points in the first assessment (T1), 54.4% of students reported experiencing burnout, while in the second assessment (T2), this figure increased to 56.2%. Indicating that higher levels of dispositional mindfulness, were associated with a reduced likelihood of experiencing burnout after a 2-year period. Conversely, those who had already experienced burnout at T1 were twice as likely to encounter it again. Furthermore, students with greater dispositional mindfulness, academic engagement, and flourishing at T1 exhibited increased odds of flourishing two years later. However, the presence of depression at T1 decreased the likelihood of flourishing in the future. | The most influential predictor of both student burnout and flourishing was found to be dispositional mindfulness. Since dispositional mindfulness can be cultivated through practice, integrating mindfulness training into undergraduate medical programs has the potential to effectively mitigate burnout and enhance the well-being of future healthcare professionals**.** |
| Purnawati et al. 2021 | Randomised Control trial | The Effect of a Problem-focused Coping Stress Management Program on Self-efficacy, Psychological Distress, and Salivary Cortisol among First-year Medical Students of Udayana University | 17 | NA | The aim of this study was to evaluate the effects of a problem-focused copping stress management program on first year medical student’s self-efficacy, psychological distress and determine effect on salivary cortisol. | Forty students displaying elevated levels of academic stress and an external locus of control were chosen at random to participate in the study. Among them, 26 were assigned to the intervention group, while 14 were placed in the control group. The intervention consisted of a problem-focused coping stress management program, delivered as a 1½-hour training session once per week over a span of four weeks. | A notable distinction in self-efficacy emerged between the intervention and control groups, with a p-value of 0.029. After the intervention, the self-efficacy score exhibited a significant increase. Similarly, a significant difference in psychological distress was observed following the intervention. The intervention group also experienced a noteworthy reduction in salivary cortisol levels after the intervention. However, post-intervention, there was no significant distinction in cortisol levels between the intervention and control groups. | The stress management program that focused on problem-solving strategies led to enhanced self-efficacy and a reduction in psychological distress and salivary cortisol levels among first-year medical students in this study. |
| Popa et al. 2020 | Case control study | The Role of Acceptance and Planning in Stress Management for Medical Students | 12 | NA | The aim of this study was to investigate the role of two coping mechanisms, namely Acceptance and Planning, in stress management among medical students. | Two groups were involved: a target group consisting of medical students (N = 100) and a control group comprising physical education and sports students (N = 100). Among these groups, 40 students with high scores in academic stress and external locus of control were randomly assigned to either an intervention (n = 26) or control (n = 14) group. The intervention involved implementing a problem-focused coping stress management program. After completing the intervention, both the target group and the control group participants filled out the COPE questionnaire. | The analysis of statistical data indicated a significant difference between the two coping mechanisms, Acceptance [mean (M) = 10.73; t (19) = 3.79, p < 0.001; confidence interval (CI) -1.91, -0.60], and Planning [M = 9.47; t (19) = 4.70, p < 0.01; CI -1.99, -0.81]. No other significant differences were found among the remaining 13 coping mechanisms, as per the statistical analysis. | Medical students effectively employ the coping mechanisms of Acceptance and Planning to manage stress during exams. These strategies not only enhance their capacity for emotional regulation but also aid them in concentrating on problem-solving tasks. |
| Polle et al 2021 | Systematic Review | Mindfulness-based stress reduction for medical students: a narrative review | NA | NA | The aim of this study was to further evaluate the benefits of MSBR for undergraduate medical students. Mindfulness based stress reduction is widely utilised standardised approach for teaching mindfulness techniques. | A comprehensive literature search was conducted across databases including MedLine, Embase, ERIC, PsycINFO, and CINAHL to identify pertinent studies. This effort yielded a total of 102 papers. Following a careful evaluation and application of predetermined inclusion and exclusion criteria, nine papers were ultimately chosen for inclusion in the study. | The training of medical students in Mindfulness-Based Stress Reduction exhibited positive outcomes, including heightened levels of psychological well-being and self-compassion. Additionally, improvements were observed in stress levels, psychological distress, and mood. However, the impact on empathy yielded mixed results, and one study examining burnout did not show any significant effect. Furthermore, two studies identified qualitative themes that offered contextual insight into the quantitative findings. | Mindfulness-Based Stress Reduction (MBSR) proves advantageous for enhancing the well-being of medical students while simultaneously reducing psychological distress and depression among them. |
| Pipas et al. 2020 | Quasi-Experimental Study | A Curriculum to Promote a Culture of Wellness Among Medical Students and Faculty | 12.5 | NA | The aim of this study was to determine the effectiveness of a comprehensive wellness program for first year medical students. | We introduce a holistic wellness initiative titled "A Culture of Wellness," designed in collaboration and with inclusiveness in mind. This pilot program was extended to both first-year medical students and faculty volunteers at the Geisel School of Medicine at Dartmouth. The participants were provided with: (1) dedicated time—60 minutes per week for a duration of 8 weeks; (2) practical resources—weekly scenarios that spotlight evidence-based wellness techniques; and (3) encouragement—platforms to openly deliberate and put these strategies into practice on a personal level and within their community. | Analysis of both pre- and post-surveys demonstrates that the combination of allocated time, collaboration between students and faculty, and the practical utilization of strategies resulted in noteworthy reductions in burnout and perceived stress levels, while simultaneously leading to elevated mindfulness and improved quality of life among participants. The curriculum was acknowledged by all participants to contribute positively to their personal well-being. | This pilot initiative introduces a practical and promising model that can be replicated in other medical institutions, offering an avenue for enhancing individual health and fostering a culture of well-being among both medical students and faculty members. |
| Phang et al. 2015 | Single-group, Prospective Study | Effects of Brief Group Mindfulness-based Cognitive Therapy for Stress Reduction among Medical Students in a Malaysian University | 15.5 | NA | The objective of this study was to assess both the practicality and success of the program in diminishing stress levels among medical students within a Malaysian university. | A cohort of 135 fourth-year medical students who were engaged in psychiatric placements took part in the program. This initiative was carried out in seven separate groups over a span of two years. The study assessed several outcome measures before and after the intervention, including mindfulness, perceived stress, and general psychological distress. | There were significant reductions in perceived stress and an increase in mindfulness with moderate effect sizes from before to after the intervention. The proportion of participants reporting substantial general psychological distress from 36% (n = 48) before the program to 10% (n = 14) post-intervention. Furthermore, while significant decreases in perceived stress were observed among both Malay and non-Malay medical students, Malay students exhibited a significantly lower level of perceived stress after completing the program. | The study's results suggest that the b-GMBCT (brief Group Mindfulness-Based Cognitive Therapy) holds promise as an efficient program for reducing stress among medical students in Malaysia. |
| Phang et al. 2015 | Randomised Control trial | Effects of a brief mindfulness-based intervention program for stress management among medical students: the Mindful-Gym randomized controlled study | 17.5 | NA | The aim of this study was to investigate the effectiveness of a brief mindfulness-based intervention program for stress management in reducing stress among students in a medical school in Malaysia. | A total of seventy-five medical students engaged in the program. They were categorized based on their years of study and then randomly assigned to the intervention group (N = 37) or the control group (N = 38). The study measured various outcome variables both before and after the intervention, including mindfulness (assessed using the Mindful Awareness Attention Scale), perceived stress (measured through the Perceived Stress Scale), mental distress (evaluated with the General Health Questionnaire), and self-efficacy (assessed using the General Self-efficacy Scale). | Significant improvements were observed in all outcome variables one week after the intervention: mindfulness, perceived stress, mental distress, and self-efficacy. Six months post-intervention, individuals who participated in the program reported higher self-efficacy compared to the control group. However, there were no differences in the other outcome measures at this point. Remarkably, over 90% of the participants found the program applicable in aiding patients, and all participants expressed their intention to recommend the program to others. | This study suggests that the program holds promise as an effective stress management intervention for medical students in Malaysia. |
| Phang et al 2015 | Randomised Control trial | Effects of a DVD-Delivered Mindfulness-Based Intervention for Stress Reduction in Medical Students: A Randomized Controlled Study | 14 | NA | The objective of this study was to assess the impact of a mindfulness-based intervention delivered through DVDs on reducing stress levels among medical students in a university in Malaysia. | A total of 76 medical students were engaged in the intervention program involving the use of the Mindful-Gym DVD. The participants were categorized based on their year of study and then randomly assigned to either the intervention group (N = 38) or the control group (N = 38). The study assessed various outcome variables both before and after the intervention, which included mindfulness (evaluated using the Mindful Attention Awareness Scale, MAAS), perceived stress (measured through the Perceived Stress Scale, PSS), mental distress (assessed using the Depression, Anxiety and Stress Scale, DASS), and self-efficacy (evaluated with the General Self-efficacy Scale, GSE). | One week following the intervention, participants within the intervention group demonstrated significantly greater improvements in self-reported levels of mindfulness, perceived stress, mental distress, and self-efficacy when compared to participants in the control group. The Number Needed to Treat (NNT) in order to attain 'normal' levels of depressive, anxiety, and stress symptoms (as indicated by DASS scores) were found to be 5, 8, and 6, respectively. | The study's results suggest that the DVD-delivered mindfulness-based program has the potential to be a labour- and cost-effective, as well as easily accessible, stress reduction program for medical students. |
| Pasarica et al. 2016 | Quasi-Experimental study | Introduction to Mindfulness: Evidence-Based Medicine Lecture and Active Session | 13 | NA | The aim of this study was to investigate the impact of an introductory session for the mindfulness technique as an evidenced-based medicine lecture combined with an active mindfulness exercise on medical students. | During this session, a PowerPoint lecture was presented, introducing mindfulness—a concept unfamiliar to most students. The lecture focused on substantiated information and featured an interactive mindfulness exercise. To gauge the lecture's impact, a survey instrument was employed. The survey aimed to evaluate participants' comprehension and inclination toward learning and applying mindfulness before and after the session. Additionally, participants' interest in incorporating mindfulness into the medical school curriculum was explored. The survey encompassed six multiple-choice questions. Engagement in the survey was voluntary and anonymous, facilitated through the use of Turning Point technology—an audience response system. | The session was delivered to first- and second-year medical students who had no previous exposure to mindfulness training. The session was led by a physician who applies mindfulness in clinical practice. Following the session, it was observed that 90% of the students expressed an interest in delving deeper into mindfulness. Furthermore, a significant majority of students indicated their enthusiasm for the integration of mindfulness into the medical curriculum. | The outcomes imply that the session can be efficiently replicated in other medical educational settings to introduce students to mindfulness. It can serve as a means to gauge students' perceptions of their knowledge in this domain and their eagerness for more comprehensive training on the subject, as well as their interest in incorporating mindfulness into medical practice. |
| Oro et al.  2021 | Non-Randomised Control Trial | Effectiveness of a Mindfulness-Based Programme on Perceived Stress, Psychopathological Symptomatology and Burnout in Medical Students | 16.5 | NA | The aim of this study was to determine the effectiveness of a Mindfulness-Based Programme on Perceived Stress, Psychopathological Symptomatology and Burnout in Medical Students. | A quasi-experimental study involving two distinct groups (experimental and control) with two instances of repeated measurement (pre and post) was conducted. Over a span of 16 weeks, eight sessions, each lasting 2 hours, were conducted. A total of 143 students took part in the study, with 68 assigned to the intervention group and 75 to the control group. Alongside a sociodemographic questionnaire, participants completed the Symptom Checklist-90-R (SCL-90-R), the Perceived Stress Scale (PSS), and the Maslach Burnout Inventory-Student Survey (MBI-SS). | Our study demonstrated a noticeable enhancement in comparison to the control group concerning perceived stress and psychopathological symptoms. Specifically, improvements were observed in the Global Severity Index, Positive Symptom Total, and primary symptom dimensions such as somatization, obsessive-compulsive tendencies, interpersonal sensitivity, and anxiety, as measured by the SCL-90-R. These enhancements were statistically significant both within the groups and between them. However, no discernible impact was observed on the level of burnout. | The implementation of the mindfulness-based program yielded positive outcomes, notably improving psychopathological symptoms and reducing stress levels. However, there was no observable impact on burnout scores. This study has the potential to inform the creation of a training program that focuses on enhancing self-care practices and effective stress management strategies for medical students and physicians alike. |
| Sugumar et al. 2019 | Quasi-Experimental study | A mental health programme for medical students | 10 | NA | The aim of this study was to outline the development of a mental health programme for medical students and obtain preliminary feedback. | The program was delivered by peers to fourth- and fifth-year medical students. The program consisted of a five-session workshop and a supplementary resource book. After the workshop an evaluate form was administered. | 19 out of 20 students responded to the evaluation form. All participants expressed consensus that the program effectively diminishes the stigma of mental health disease in medicine. It also enhances peer support and self-care practices. All students concurred that they would endorse the program to their peers, highlighting its valuable role in facilitating open discussions about mental health and normalizing concerns related to mental well-being | The program serves as an illustration of a innovative and successful design that can serve as a model for medical educators seeking to provide comprehensive mental health skills support to upcoming medical professionals. |
| Stewart et al. 2018 | Quasi-Experimental study | Experiences with a universal mindfulness and wellbeing programme at a UK medical school | NA | 26 | The aim of this study was to investigate the impact of a universal, mental wellbeing and mindfulness programme at a UK medical school, | This study employed mixed methods, encompassing the assessment of mental well-being and mindfulness within two groups, observed at three intervals spanning 15 months. The research approach involved various analytical techniques, including descriptive, regression, and repeated measures analyses, followed by post hoc pairwise comparisons. Additionally, qualitative interviews were conducted with a selected group of 13 students after one year, and their responses were thematically analysed. The study also incorporated spontaneous and anonymous feedback on the course as part of its data collection process. | Appreciation for the course content increased during the initial year. A majority of students had actively integrated a well-being strategy by the course's conclusion, and around 25% were participating in mindful activities. Despite an overall decrease in well-being and limited involvement in mindfulness practices, heightened mindfulness levels served as a safeguard against this decline for both groups.  A small minority of students believed the course had little value, impacting their peers' engagement. The attitudes of these students influenced the participation of others. Students noticed and were influenced by the well-being and mindfulness practices demonstrated by the course facilitators, which affected their perceived outcomes. | The results are promising, indicating a moderate positive impact in terms of shifting attitudes and behaviours, along with a moderate protective influence on the well-being of students who actively participated. |
| Staffaroni et al. 2017 | Cross-Sectional Study | Long-term follow-up of mind-body medicine practices among medical school graduates | 12.5 | NA | The aim of this study was to investigate the continued utilisation of mind-body medicine (MBM) skills following the completion of medical school. | We conducted an online survey targeting graduates of Georgetown University School of Medicine (GUSOM) who had undertaken a minimum of one semester in a mind-body medicine (MBM) skills training course. Employing a mixed-methods approach that combines quantitative and qualitative analysis, we examined the graduates' personal and professional application of the skills learned, and identified potential factors affecting their continued practice and training post-graduation. | Existing individual practices were found to have a positive correlation with the extent of course completion and the frequency of practicing MBM skills during medical school (N=112). More than half of the participants reported ongoing MBM practice, including referring patients to MBM techniques. Furthermore, the specialization of physicians and their knowledge of MBM training at their respective institutions influenced their training and referral of patients for MBM practices. Participants noted a lack of comprehensive MBM training within their institutions, and they shared qualitative perspectives on the personal and professional significance of MBM training, along with obstacles to sustaining MBM practice. | The findings offer initial indications that undergoing MBM training during medical school could potentially have a lasting connection with the self-care practices of physician trainees and their approach to patient care. However, more rigorous investigations are required to thoroughly examine these associations in future studies. |
| Sparshadeep et. al 2019 | Quasi-Experimental study | Qualitative study on perception of first-year medical undergraduates toward mentorship program | 12 | 25 | The aim of this study was to investigate the perception of first year undergraduate medical students towards a mentorship program. | A parallel mixed-method design was employed, beginning with an initial quantitative survey, succeeded by a qualitative investigation through focused group discussions. Conducted over a span of 3 months among first-year medical students The survey involved a total of 96 participants who grouped into focus groups consisting of 7 to 9 students each. Three such groups took part in FGD sessions until data saturation was achieved. The collected data were subjected to analysis using both quantitative and qualitative methods. | Results revealed that while the program received low satisfaction ratings in terms of time management (12.5%) and stress management (19.8%), a significant percentage of students still held a positive liking for the program (69.8%) and acknowledged its necessity (72.9%). The discussions identified several factors that could facilitate success, including mentor involvement, genuine interest, mentors' positive attitudes, encouragement, guidance, as well as obstacles such as infrequent mentoring sessions, absence of follow-up, inadequate communication, and limited personal interaction. | Students' perspectives played a crucial role in recognizing both the strengths and shortcomings of a mentorship program. The insights gathered from focused group discussions served as a foundation for proposing strategies to enhance and refine the program. These recommendations primarily revolved around enhancing communication and strengthening the mentor-mentee relationship. |
| Dobkin et al. 2013 | Literature Review | Teaching mindfulness in medical school: where are we now and where are we going? | NA | NA | The aim of this study was to examine the literature and related website to determine the extent at which mindfulness is carried out with medical students and residents. | Through a comprehensive review of existing literature, it was discovered that a total of 14 medical schools are currently incorporating mindfulness training into their curriculum for medical and dental students as well as residents. | Various formats are employed to teach mindfulness, encompassing methods such as basic lectures, one-day workshops, and comprehensive 8 to 10-week programs like mindfulness-based stress reduction. Notably, two medical schools have particularly distinguished themselves by integrating mindfulness into their curricula: the University of Rochester School of Medicine and Dentistry in the USA, and Monash Medical School in Australia. Research indicates that students who participate in these programs report reduced psychological distress and an enhanced quality of life. | While the evidence underscores the value of instructing mindful practices, there are several important considerations that still need attention. For instance, determining the optimal timing within a doctor's career to introduce mindfulness training, identifying the most effective formats for specific contexts and individuals, and establishing methods for sustaining the acquired knowledge over time are crucial areas to address. Moreover, the question arises whether mindfulness training should be integrated into the core curriculum of medical schools. |
| Neufeld et al. 2020 | Quasi-Experimental study | Basic psychological needs, more than mindfulness and resilience, relate to medical student stress: A case for shifting the focus of wellness curricula | 14 | NA | The aim of this study was t investigate the association between medical student’s mindfulness, resilience, basic psychological needs and perceived stress/ | Out of all the medical students spanning years 1 to 4 in our institution, a total of 197 students (49% of the total) participated in an online survey. Aimed at gauging the levels of satisfaction and frustration associated with their fundamental psychological needs, which include autonomy, competence, and relatedness. The study also evaluated their mindfulness, resilience, and perceived stress. The collected variables were analysed in connection with perceived stress levels, with considerations made for the students' gender and academic year. | Elevated levels of mindfulness, resilience, and satisfaction of fundamental psychological needs were linked to lower levels of perceived stress. Conversely, the experience of frustration in meeting these psychological needs was associated with higher perceived stress. Among the different academic years, third-year students reported a higher sense of frustration in terms of autonomy compared to students in other years. In terms of gender differences, female students in the second and fourth years reported heightened stress levels, reduced mindfulness and resilience, and a lesser sense of competence fulfilment in comparison to male students. | The results from this study indicate that mindfulness and resilience play significant roles in promoting the well-being of medical students. However, it's noteworthy that the protective effects of these qualities against stress might weaken when the students' fundamental psychological needs are not adequately met within the medical school environment. As a result, it is advisable to address aspects of the learning environment that could potentially hinder these psychological needs. By doing so, it could potentially reduce stress levels among students and enhance their overall well-being. |
| Orosa-Duarte et al. 2021 | Randomised Control Trial | Mindfulness-based mobile app reduces anxiety and increases self-compassion in healthcare students: A randomised controlled trial | 18 | NA | The aim of this study was to assess and compare the effect of a mindfulness-based mobile application versus an in-person mindfulness-based training program in terms of reducing anxiety and increasing empathy, self- compassion, and mindfulness in a population of healthcare students. | The authors conducted a single-blind, randomized controlled trial featuring three distinct parallel groups. Participants were assigned to one of three groups: the mobile app group, the in-person mindfulness-based program (IMBP) group, or a control group. The study involved baseline assessments as well as post-intervention evaluations conducted at an 8-week interval. These evaluations encompassed measurements of anxiety levels, empathy, self-compassion, and mindfulness across all the groups. | Out of the initial 168 randomized students, 84 were included in the analysis on an intention-to-treat basis. This comprised 31 participants in the mobile app group, 23 in the (IMBP) group, and 30 in the control group. Notably, the mobile app group displayed a substantial effect size in terms of reducing trait anxiety when compared to the control group. A moderate effect size was observed in comparison to the IMBP group, although it wasn't statistically significant. Both the mobile app and IMBP groups exhibited a noteworthy increase in self-compassion and mindfulness levels in contrast to the control group. However, there was no notable change in empathy levels observed across all three groups. | The study suggests that a mobile app can be just as effective as (IMBP) in terms of reducing anxiety and enhancing self-compassion and mindfulness among healthcare students. The results indicate that both approaches yield comparable positive outcomes in these psychological and emotional aspects for the student population. |
| Moore et al. 2020 | Quasi-Experimental study | Determining the feasibility and effectiveness of brief online mindfulness training for rural medical students: a pilot study | 15.5 | 21 | The aim of this study was to determine the practicality and effectiveness of a mindfulness training program, delivered online to medical students at a Rural Clinical School. | In 2016, an 8-week online training program was administered to penultimate-year medical students at an Australian Rural Clinical School. Employing a mixed methods approach, the study evaluated participants' engagement with mindfulness meditation practice in terms of frequency and duration. Additionally, changes in perceived stress, self-compassion, and compassion levels were assessed, along with shifts in personal and professional attitudes and behaviours. This comprehensive approach aimed to capture both quantitative and qualitative insights into the effects of the training program. | A total of forty-seven participants were enrolled in the study. By the conclusion of the program, half of the participants were engaging in mindfulness meditation practice on a weekly basis. Even four months after the intervention, 32% continued to practice mindfulness meditation weekly. Notably, a statistically significant reduction in perceived stress levels was observed, while self-compassion levels notably increased during the 4-month follow-up period. Furthermore, participants shared their observations regarding the individual and professional effects of mindfulness meditation training. They also highlighted certain obstacles that they encountered in sustaining their practice. | The findings from this study offer initial indications that online mindfulness meditation training could potentially lead to decreased stress levels and heightened self-compassion among rural medical students. However, further comprehensive research is necessary to establish solid feasibility measures for implementing a mindfulness meditation program. This could involve more robust investigations to validate and refine the effectiveness of such training in enhancing well-being among this specific student population. |
| Wright et al. 2019 | Literature Review | Training medical students to manage difficult circumstances- a curriculum for resilience and resourcefulness? | NA | NA | The objective of this study was to develop a novel approach for thoroughly assessing a five-year undergraduate medical curriculum, focusing on the presence of teaching, learning objectives, and experiences that aim to cultivate resilience among medical students. It was then determined whether this methodology could facilitate curriculum discussions, leading to the enhancement of training for upcoming cohorts. As well as to explore the potential for introducing a dedicated curriculum designed to foster resilience and resourcefulness among medical students. | Drawing insights from the existing literature, a rating scale was developed to elicit quantitative data related to four aspects of resilience: internal resources, lifestyle factors, self-mediated external resources, and agent-mediated external resources. This scale was employed to assess the entirety of a five-year undergraduate medical curriculum offered by a northern England medical school. The methodology adopted a structured four-stage approach: Identifying the learning objectives, correlating these learning objectives with the predefined criteria mentioned earlier, evaluating these learning objectives against specific, well-defined standards that are planned, explicit, universal, and quantifiable, applying a rating system to the collected data based on the assessments made in the previous stages. | The assessment yielded a distinct and quantifiable overview of how the curriculum contributes to resilience development. This process successfully pinpointed both strong aspects and areas requiring improvement. Subsequent efforts were directed toward addressing these findings, resulting in proposed modifications. These insights played a pivotal role in fostering productive conversations with course leaders and planners. The feedback received was universally positive and instrumental in identifying new learning objectives, activities, and experiences. The implementation of these suggestions has already commenced, signifying a proactive step toward enhancing the curriculum to better promote resilience among students. | The "HYMS CARE Criteria" and the methodology we employed to evaluate it within the context of a medical school curriculum present a valuable standpoint that can assist in refining curriculum enhancements. This model, which outlines and organizes resilience-related learning experiences, serves as a potential framework for other educational institutions to contemplate and potentially adopt. |
| Rong et al. 2021 | Randomised Control Trial | Improvement of the management of mental well-being and empathy in Chinese medical students: a randomized controlled study | 16 | 15 | The aim of this study was to determine the efficacy of the intervention courses designed to enhance the mental health and empathy of senior Chinese medical students. | A total of 146 medical students were randomly assigned to either an intervention group (n = 74) or a control group (n = 72). Prior to this, a pilot study was conducted involving 5 pre-clinical students and 5 interns to determine the themes and content for the intervention courses. The intervention group received these designed courses once a month for a total of three sessions, while the control group did not receive any specific intervention. Five self-assessment questionnaires were administered, including the General Self-Efficacy (GSE) scale, Medical Outcomes Study Short Form 8 (SF-8), Patient Health Questionnaire-9 (PHQ-9), Maslach Burnout Inventory (MBI), and Jefferson Scale of Empathy-Health Care Provider Student version (JSE-HPS). These were completed both before and one month after the intervention courses, Qualitative data was collected through email correspondence two years after the intervention took place. | In comparison to the control group, the intervention group demonstrated notably higher scores in empathy (mean score: 111.0 [IQR 102.0, 118.0] vs. 106.0 [IQR 93.0, 111.5]; P = .01) and quality of life (mean score: 32.0 [IQR 28.0, 35.0] vs. 29.5 [IQR 26.0, 34.0]; P = .04). The incidence of depression was notably lower in the intervention group in contrast to the control group (13.5% vs. 29.2%; chi-square test, P = .02). However, there were no significant differences in self-efficacy (mean score: 25.6 ± 4.8 vs. 24.3 ± 6.3; P = .16) or burnout (27.0% vs. 34.7%; Chi-square test, P = .31) observed between the two groups. | The intervention courses yielded positive outcomes in terms of mental well-being and empathy among senior Chinese medical students. These findings hold the potential to contribute novel insights that could inform the inclusion of such intervention courses within the medical school curriculum. |
| Worobetz et al. 2020 | Non-Randomised Control Trial | A feasibility study of an exercise intervention to educate and promote health and well-being among medical students: the ‘MED-WELL’ programme | 13.5 | NA | The aim of this study was to determine the practicality of an exercise intervention to educe and promote health and well-being among medical students. | The 'MED-WELL' program consisted of a six-week curriculum comprising weekly hour-long sessions. Each session centred around a different type of physical activity, lasting 45 minutes. Each session began with a 15-minute interactive lecture on integrating exercise theory into daily medical practice. The study focused on first and second-year graduate entry medical students at the University of Limerick (UL). Three feasibility parameters were employed to evaluate the program: 1. Recruitment and retention of participants. 2. Acceptance of the program. 3.Efficacy concerning health and well-being, which was assessed by administering pre- and post-intervention questionnaires. The study utilized the following validated measurement scales: EQ-VAS; WHO-5 Well-Being Index; 3-item Loneliness Scale; Social Support Measure 3-item scale. Free-text sections in the questionnaires encouraged participants to provide feedback on the program's advantages. | Out of a total of 286 students, 26% (74 students) participated in the 'MED-WELL' program. Among those who took part, 93% (69 students) attended at least one session of the program and completed questionnaires both before and after the intervention. The study observed significant improvements in various aspects of well-being and health following the program. The WHO-5 Well-Being Index scores increased from 63.2 to 67.5. Sleep quality, as indicated by the sleep scale, improved from 3.1 to 3.5. Feelings of loneliness decreased, with the loneliness scale scores declining from 4.1 to 3.5. Additionally, students' levels of physical activity during a typical week increased from 3.7 to 4.0. These findings suggest that the 'MED-WELL' program had a positive impact on the well-being, sleep quality, loneliness, and physical activity levels of participating medical students. | The study has demonstrated the feasibility of implementing the 'MED-WELL' program within a medical school curriculum. Moreover, it suggests that the program offers benefits and is well-received by students. However, to further assess the program's effectiveness, its lasting impact, and cost-effectiveness, well-designed randomized controlled trials are warranted. These trials would provide more robust evidence regarding the program's outcomes and its potential to promote long-term well-being among medical students. |
| Lo et al. 2017 | Systematic Review | Group interventions to promote mental health in health professional education: a systematic review and meta- analysis of randomised controlled trials | NA | NA | The aim of this study was to analyse interventions to support mental health of health professional students and their effects. | The study conducted a search of the databases of Medline, PsycINFO, EBM Reviews, Cinahl Plus, ERIC, and EMBASE up until April 15th, 2016. The inclusion criteria encompassed randomized controlled trials involving undergraduate and postgraduate health professional students. These trials involved group interventions aimed at supporting mental health, and they were compared to alternative educational methods, usual curriculum, or no intervention. Post-intervention assessments were required for both intervention and control groups, focusing on measures of mindfulness, anxiety, depression, stress/distress, or burnout. The data were synthesized through meta-analysis. | Four main comparisons were identified, which included psychoeducation or cognitive-behavioural interventions compared to alternative education, as well as mindfulness or relaxation interventions compared to control conditions. Revealing the following outcomes for the different interventions: Cognitive-behavioural interventions were associated with a reduction in anxiety, depression, and stress. Mindfulness strategies were effective in reducing stress, but they did not significantly impact anxiety, depression, or burnout. Relaxation strategies showed reductions in anxiety, depression, and stress. It's important to note that the methodological quality of the included studies was generally assessed as poor, which could potentially affect the robustness of these findings. | The available evidence suggests that cognitive-behavioural, relaxation, and mindfulness interventions have the potential to support the mental health of health professional students. However, it's important to note that the quality of the research conducted so far has been assessed as generally poor. Therefore, there is a need for further high-quality research in this area to provide more definitive insights into the effectiveness of these interventions in promoting the mental well-being of health professional students. |
| Buck et al. 2020 | Quasi-Experimental study | The physician healer track: educating the hearts and the minds of future physicians | 11.5 | NA | The aim of this study was to investigate the effect of The Physician Healer Track, 500-contact-hour curricula integrated over 4 years, focusing on self-awareness, reflection, being-with- suffering, communication and professional identity development. | This program incorporates training in various areas, including mindfulness, cognitive-behavioural therapy, nonviolent communication, motivational interviewing, spirituality in healthcare, wellness, equanimity, and the concept of 'being with suffering.' These topics are reinforced throughout all four years of the program. The training places significant emphasis on community building and reflection, which are integrated into both monthly sessions and immersion courses. The program has experienced enrolment growth, starting with 26 students in the first year (11% of the class) and eventually expanding to a total of 258 students over the initial six years. On average, this represents a participation rate of 20-26% of each incoming class, indicating a substantial and sustained interest among students. | Graduates from the first two cohorts of PHT have exceeded the numbers in the eight other scholarly concentrations offered at UTMB, indicating a high level of student interest and engagement., Attrition was remarkably low, with less than 1% dropping out, indicating strong program commitment. Additionally, students consistently report ongoing growth in personal development, professional development, and their ability to empathize, suggesting that the program has a positive and sustained impact on their personal and professional growth. The introduction of PHT has not only benefited medical students but has also resulted in the expansion of training opportunities for medical residents, faculty, and physical therapy students. | Despite the rigorous demands on students' time, they are willingly and actively participating in a challenging program of integrated training. Their primary intention is to remain connected to their humanity throughout the demanding journey of medical school training. This commitment underscores the program's value and its positive impact on students' personal and professional development. |
| Chen et al. 2017 | Non-Randomised Control Trial | The effect of Mind Body Medicine course on medical student empathy: a pilot study | 14 | NA | The aim of this study was to investigate the effects of a Mind Body Medicine (MBM) course on perceived stress and empathy on first year medical students. | 13 first-year medical students voluntarily self-selected to participate in the (MBM) group (experimental), while seven students chose not to participate and formed the non-MBM (control) group. The study employed a prospective design and conducted pre- and post-test analyses using three measurement tools: the Jefferson Scale of Physician Empathy for Students (JSPE-S) to assess empathy, the Perceived Stress Scale (PSS) to measure stress levels, and the Personal Health Questionnaire (PHQ) to evaluate depression. These assessments were used to track changes in empathy, stress, and depression levels before and after the intervention or program. | The study's findings indicated a notable trend of increased stress levels and decreased empathy among both the Mindfulness-Based Medicine (MBM) group and the non-MBM group over the course of the study. | The results revealed an inverse relationship characterized by increased stress and decreased empathy among first-year medical students. Importantly, participation in the (MBM) course did not appear to mitigate these changes. However, while both groups experienced these stress and empathy changes, a statistically significant increase in depression scores was observed in the non-MBM group, but not in the MBM group. This suggests that the MBM course may have had a protective effect against the increase in depression seen in the non-MBM group. |
| Cameron et al. 2019 | Quasi-Experimental study | Executive/life coaching for first year medical students: a prospective study | 14 | NA | The study aimed to prospectively assess the feasibility and acceptability of a structured, theory-based executive/life coaching program specifically designed for first-year medical students in the United States. Secondary objectives included evaluating the program's impact on participants' coaching goals, resilience, and perceived stress levels. | This single-arm intervention study assessed the effectiveness of a program involving two group coaching sessions and two private coaching sessions. The program was administered during the first year, second semester, and targeted students from the Georgetown University School of Medicine Class of 2019. Data were collected from participants through surveys, including global and tailored questions, as well as assessments using the Connor-Davidson Resilience Scale and the Friedricksson-Larsson Stress Questionnaire. Data collection occurred both at baseline and post-intervention to evaluate the program's impact. | The majority of participants expressed a willingness to recommend the program. Most participants also recommended including the program in the curriculum. Statistically significant increases were observed in self-efficacy related to stress management and increased awareness of thoughts about stress and their management. Reported improvements in time management and energy for relationships and school did not reach statistical significance. The global resilience rating did not show significant changes, but significant changes were noted in the factors of control and spiritual influence. 40.6% of participants reported decreased stress, and another 40.6% reported no change in stress levels during this challenging preclinical semester. | The study demonstrated that a tailored executive/life coaching program designed for first-year medical students in the United States is not only feasible but also well-tolerated and safe. Adherence to the program was excellent, and participant feedback, as indicated by global utility ratings and willingness to recommend coaching, strongly supports its potential efficacy. However, it's important to acknowledge that more robust measures and larger-scale clinical trial designs are necessary to establish formal proof of the program's effectiveness and to provide more comprehensive evidence of its benefits. Further research is needed to confirm and expand upon these initial positive results. |
| Young et al. 2019 | Editorial | Requiring wellness: implementation of a comprehensive wellness curriculum | NA | NA | NA | NA | NA | NA |
| Ball et al. 2002 | Quasi-Experimental study | Self-care in Medical Education: Effectiveness of Health-habits Interventions for First-year Medical Students | 14.5 | NA | The aim of this study was to investigate how health habits, including sleep, alcohol consumption, and exercise, changed among first-year medical students. Additionally, the study aimed to assess the impact of an educational intervention that promotes self-care on the emotional well-being and academic adjustment of these students. | A total of 54 medical students participated. They were asked to complete questionnaires at three different points in time: at the beginning of the semester, at mid-term, and at the end of the semester. These questionnaires assessed various health habits, including sleep, alcohol use, depression severity, and areas of life satisfaction. Approximately half of the students received either written feedback or participated in an educational discussion group at the mid-term point in the semester. | There were increases in alcohol consumption and decreases in exercise and socialization across the semester. These changes in health habits were associated with both emotional and academic adjustment. Students who exhibited a decrease in positive health habits, particularly in terms of socialization, tended to report higher levels of depression at the end of the semester (finals). While the feedback and educational interventions did have some influence on sleep and exercise behaviors, there were no significant differences between the groups in terms of overall emotional or academic adjustment | First-year medical students experience significant changes in their health habits as they adapt to the demands of medical school. While an educational intervention showed promising effects in altering these patterns, it's clear that the concept of self-care needs further development to address the specific challenges associated with both the acute adjustment period and the long-term stressors that medical students face. This suggests that more tailored and comprehensive approaches to self-care are necessary to support the well-being of medical students throughout their medical education journey. |
| Moser et al. 2009 | Quasi-Experimental study | Teaching Behaviour Change Concepts and Skills During the Third-Year Medicine Clerkship | 5.5 | NA | The aim of this study was to determine the effectiveness of teaching behavior change concepts and skills during the third-year medicine clerkship. | The authors developed the Health Beliefs and Behavior (HBB) course with several key objectives. Including teaching students about the impact of unhealthy behaviors on health and wellness, broadening their understanding of the various factors that influence behavior, and equipping medical students with tools to facilitate health behavior change in their patients. The HBB course was integrated into the four-week, third-year internal medicine clerkship ambulatory block. This approach allowed students to apply the techniques they learned in both clinical and classroom settings. They were also able to gain insights into health behavior by applying health models to real patients and engaging in experiential exercises. | All but one of the students, totaling 149 participants, completed surveys during the academic year 2006–2007. The project received Institutional Review Board (IRB) approval from UMDNJ. The data from the 2006–2007 survey is summarized in Table 2. In general, the third-year students reported an enhanced ability to provide recommendations for health promotion and behavioral change strategies to their patients, along with an improved understanding of the main principles taught in the course. | This study represents the first report of a comprehensive course, complemented by clinical experiences, dedicated to teaching the theory and practice of behavior change. Third-year students at UMDNJ-NJMS who completed the (HBB) course reported an improved understanding of the course's core principles and an enhanced ability to recommend health promotion and behavioral strategies to their patients. However, further research is necessary to determine whether this shift in attitudes and increased confidence in utilizing behavioral change tools results in students applying these techniques more frequently in their current practices and future medical careers. |
| Phillips et al. 2015 | Short Communication | Including lifestyle medicine in undergraduate medical curricula | NA | NA | The aim of this study was establishing a comprehensive and standardized LM education in medical schools to address these health challenges. | A plan is currently being developed to integrate lifestyle medicine (LM) into medical school education, and this effort involves collaboration with various stakeholders. These stakeholders include medical school deans and students, medical curriculum developers and researchers, medical societies, governing bodies, and policy institutes Involving a series of three planning and strategy meetings, each addressing specific key areas of focus. Particular emphasis is being placed on nutrition, physical activity, student self-care, and behavior change. The goal is to develop concrete implementation guidelines and milestones to facilitate the integration of LM into medical education effectively. | Following the first two meetings, the proposed areas of focus for integrating lifestyle medicine (LM) into medical school education were identified as follows: Supporting deans and key personnel. Establishing federal and state policy commitments. Utilizing assessment as a driver for LM. Providing high-quality evidence-based curricular material on an easily accessible platform. Engaging student interest in Implementation strategies for each of these focus areas will be discussed and developed further during an upcoming planning meeting scheduled for early 2015. This collaborative effort aims to create a comprehensive plan for effectively integrating LM principles into medical education. | This initiative is anticipated to have significant public health implications as it aims to efficiently promote the prevention and treatment of non-communicable chronic diseases. The goal is to establish a scalable and sustainable model for educating physicians in both training and practice. By integrating lifestyle medicine principles into medical education, this initiative seeks to empower future and current healthcare providers with the knowledge and tools to address chronic diseases effectively, ultimately contributing to improved public health outcomes. |
| Coleman et al. 2021 | Quasi-Experimental Study | Brief, effective experience to increase first-year medical students’ nutrition awareness | 15.5 | NA | The aim of this study was to determine the impact of a brief nutrition-based education experience for first-year students offered at the start of the medical school curriculum, designed to increase their nutrition awareness. | The nutrition education experience consisted of five key components:1) Students were required to complete three 24-hour food recalls to assess their dietary habits. 2) These recalls were then compared to established nutrient standards to evaluate the nutritional quality of their diets. 3) The education emphasized strategies for making simple yet nutritionally sound food choices and effective food preparation. 4) Students were surveyed to gather information on their implementation of personal healthy nutritional strategies. 5) Finally, students were asked to provide recommendations for future modifications to enhance the educational experience. This comprehensive approach aimed to assess, educate, and engage students in improving their nutritional knowledge and habits. | The assessment revealed that the diets of most students did not meet the recommended levels for various nutrients, and these deficiencies were associated with specific inadequacies in food groups. A follow-up survey conducted three months later received responses from 40% of the students. Among these respondents, 46% reported implementing at least one of the strategies presented during the nutrition education program to improve their dietary intake. The changes they made typically involved adding or removing specific foods from their diets. Additionally, a significant majority, specifically 73%, recommended repeating the program in the future, indicating its perceived value and effectiveness. | This study demonstrates that a concise 2.5-hour nutrition wellness program can effectively raise nutrition awareness and encourage dietary improvements among incoming medical students. The majority of participants found the experience valuable, and many recommended providing a similar program to future classes. This suggests that even a relatively brief educational intervention can have a positive impact on students' nutrition knowledge and habits, highlighting the potential for integrating nutrition education into medical curricula to promote healthier eating behaviors among future healthcare professionals. |
| Mukunda et al. 2019 | Narrative Review | Visual art instruction in medical education: a narrative review | NA | NA | The aim of this study was to evaluate the current use of humanities, specifically visual arts training in medical school. | To gain insight into the current utilization of visual arts training in medical education, we conducted a comprehensive review of the existing literature. This review encompassed both qualitative and quantitative data. | Common themes that emerged included a focus on preclinical students; instruction promoting observation, diagnostic skills, empathy, team building, communication skills, resilience, and cultural sensitivity. Successful partnerships have involved local art museums, with sessions led primarily by art educators employing validated pedagogy such as Visual Thinking Strategies or Artful Thinking. There is evidence that structured visual arts curricula can facilitate the development of clinical observational skills, although these studies are limited in that they have been single-institution reports, short term, involved small numbers of students and often lacked controls. | There is a paucity of rigorous published data demonstrating that medial student art education training promotes empathy, team building, communication skills, wellness and resilience, or cultural sensitivity. Thus, further research on stronger and evidence-backed methods for incorporating visual arts education into the training of medical students should be performed. |
| Drolet et al. 2010 | Editorial | A comprehensive medical student wellness program--design and implementation at Vanderbilt School of Medicine | NA | NA | The aim of this study is to report the design and implementation of a comprehensive medical student wellness program at Vanderbilt School of Medicine. | This program comprises three fundamental parts: The Advisory College Program, The Student Wellness Committee, and VMS LIVE. While each of these core components has distinct individual programs, they work together collaboratively to achieve the overarching wellness objective of enhancing student health, well-being, and their full potential. | The VMS Wellness Program has had early success with substantial growth and outstanding student buy-in since its inception in 2005. Preliminary data indicate that nearly every student has participated in at least two components of the VMS Wellness Program. In addition to participation, student response has been highly satisfactory, as evidenced by their positive feedback. | The VMS Wellness Program represents the inaugural published model of a comprehensive wellness initiative tailored specifically for medical students. The program's development and structure, as detailed in this article, can serve as a blueprint or template for other educational institutions seeking to establish similar initiatives. |
| Kalén et al. 2010 | Quasi-Experimental Study | Mentoring medical students during clinical courses: A way to enhance professional development | 12 | NA | The aim of this study to evaluate undergraduate medical students’ experiences and perceptions of one-to-one mentoring and whether they felt that the mentorship promoted their personal and professional development. | In this study, a group of 118 medical students in their third and fourth years of study were provided with individual mentors for a duration of two years. After the mentoring program concluded, these students were surveyed using a questionnaire. Data analysis was carried out using statistical software, and responses to open-ended questions were examined through content analysis techniques. | Most participants reported that the mentoring program was beneficial for their professional and personal growth. They perceived the mentor's role as primarily providing support rather than just imparting knowledge. Students valued the opportunity to engage with a faculty member who wasn't directly involved in their coursework. The limited challenges to effective mentorship mainly revolved around scheduling logistics and the compatibility of personalities, often referred to as 'personal chemistry.' | The results suggest that individual mentoring during clinical courses positively influences the professional and personal development of medical students. However, further research is warranted to gain a more comprehensive understanding of the factors critical to the success of mentorship programs. |
| Drusin et al. 2013 | Quasi-Experimental Study | An advisory program for first- and second-year medical students: the Weill Cornell experience | 8.5 | NA | The aim of this study was to evaluate a program for first- and second-year medical student. | In this initiative, fifty faculty advisors were each allocated a group of one to three first-year students whom they would mentor for a span of two years. Clear responsibilities were communicated to both the faculty advisors and the students. The program's effectiveness was assessed through an anonymous questionnaire. | It was found that the majority of students met with their advisors at least once. The most common topics discussed during these meetings were related to adjusting to medical school, academic challenges, and insights into the advisor's professional life. Additionally, discussions about summer research opportunities and career prospects were prevalent. Overall, most students expressed satisfaction with the advising program, and their satisfaction tended to rise with more frequent meetings. For those who didn't initially meet their advisors, many students took the initiative to establish a mentorship relationship independently. | Weill Cornell Medical College implemented an advising program that received positive feedback from the majority of students. To maintain its effectiveness, it's crucial to conduct periodic evaluations of the program's format, considering the viewpoints of both students and advisors. This ongoing assessment is essential to ensure the program's continued success and relevance. |
| Fornari et al. 2014 | Cross-Sectional Study | Mentoring program design and implementation in new medical schools | 10 | NA | The aim of this study was to investigate the structure and implementation of mentoring programs in medical schools. | A survey was conducted among administrators from 14 medical schools in the United States, all of which were established after 2006. The survey aimed to gather information about the design and execution of mentoring programs within these institutions. | Most of the recently established medical schools had mentoring programs in place, but these programs differed in their structure and implementation approaches. While these programs were generally considered valuable by their respective institutions, several challenges were identified during their creation and implementation. These challenges included constraints on time for both faculty and students, as well as a shortage of financial and professional incentives for faculty members to participate actively in mentoring roles. | Similar to established medical schools, new medical schools also exhibited a lack of uniformity in their mentoring programs, which may be attributed to variations in curriculum and program objectives. To establish a best practice for mentoring in medical education, it's essential to introduce outcome measures that can assess the effectiveness of these programs. This way, the impact and success of mentoring initiatives can be evaluated systematically. |
| Abrams et al. 2020 | Editorial | Peer Support Expands Wellness Services and Reduces Mental Health Stigma | NA | NA | NA | NA | NA | NA |
| McEvoy et al. 2016 | Randomised Control Trial | Near-peer role modelling: Can fourth-year medical students, recognized for their humanism, enhance reflection among second-year students in a physical diagnosis course? | 12 | 18 | The aim of this study was to determine if fourth-year medical students, recognized for their humanism, enhance reflection among second-year students in a physical diagnosis course. | A total of 186 students were randomly divided into three different comparison groups. The first group was facilitated by a fourth-year student who was a member of the Gold Humanism Honor Society (GHHS). The second group, also, was facilitated by a volunteer student who was not a GHHS member. The third students and did not have facilitators. Before their sessions, second-year students established learning goals related to their patient interactions. Fourth-year students who were acting as facilitators received training materials for their role. These groups met twice during ten clinical site visits. At the final session, students completed a reflective assignment regarding their progress toward their goals. The study conducted mixed-method analyses to compare the three groups based on reflection scores from the in-session assignment, session satisfaction, and a thematic analysis of responses provided in the in-session assignment. | The study revealed significant differences among all three comparison groups in terms of students' reflective scores (p < 0.0003) and satisfaction (p < 0.0001). When comparing the groups facilitated by GHHS members and those facilitated by non-GHHS students using t-tests, it was found that GHHS-facilitated groups had significantly higher mean reflective scores (p < 0.033), but there were no differences in session satisfaction. A thematic analysis of students' reflections indicated that they made efforts towards self-examination but lacked depth in addressing their emotions. Common themes among the reflections included a strong emphasis on gaining comfort and confidence in their clinical skills performance. | The study found that near peers, particularly those acknowledged for their humanistic qualities, played a substantial role in enriching the reflections of medical students concerning patient interactions and humanistic development. In general, students favored peer feedback forums with facilitation over non-facilitated ones. This model shows potential for improving self-reflection in medical education, although more research is required to assess its impact on behavior. |
| Ferguson et al. 2022 | Quasi-Experimental Study | REACH: A Required Curriculum to Foster the Well-Being of Medical Students | 9 | NA | The aim of this study was to determine the impact of REACH: A Required Curriculum to Foster the Well-Being of Medical Students. | The REACH curriculum is a compulsory, ongoing well-being program for first- and second-year medical students at the Medical College of Wisconsin. The curriculum is based on principles, core ideas, and skills derived from the field of trauma stewardship. It leverages effective well-being interventions for medical students, including mindfulness training, and incorporates personal narratives shared by instructors during formal lectures and small-group sessions, which are seamlessly integrated into the regular MCW curriculum. The curriculum's effectiveness was assessed over the initial two years of implementation through surveys administered to students before and after their participation in the program. | The REACH curriculum was completed by over 700 students. The surveys conducted revealed that most students who participated believed that the curriculum content was important, that it met their expectations as a, and that they would recommend other institutions to incorporate a similar curriculum. Furthermore, the responses from the post-curriculum survey conducted showed that students felt that the REACH curriculum had a positive impact on their development of self-care skills (84%), mindfulness (76%), and the ability to seek help when needed (71%). | The initial findings indicate that introducing a mandatory well-being curriculum is not only possible but also well-received by medical students. The authors of the program intend to further investigate the connections between well-being metrics reported by students, their academic and clinical performance data, and how it influences the development of their professional identities. Additionally, they are in the process of developing electronic dashboards that will enable students to engage with their well-being data. This interactive platform aims to encourage students to seek assistance when needed and promote changes in their behavior to enhance their well-being. |
| Wang et al. 2020 | Quasi-Experimental Study | Implementation of the college student mental health education course (CSMHEC) in undergraduate medical curriculum: effects and insights | 15 | 17 | The aim of this study was to investigate the effectiveness of the college student mental health education course (CSMHEC) in undergraduate medical curriculum: | This study follows a quasi-experimental design, incorporating both quantitative and qualitative analyses. The participant pool consisted of 374 first-year medical students, with 188 students assigned to the experimental group and 186 students assigned to the control group. For the quantitative analysis, several standardized assessments were employed, including the Depression Anxiety Stress Scales-21 (DASS-21), Chinese College Student Academic Burnout Inventory (CCSABI), and the Satisfaction with Life Scale (SWLS). Additionally, a 5-point Likert scale was utilized to gauge students' overall satisfaction with the Chinese Student Mental Health Education Curriculum (CSMHEC). In the qualitative analysis, a thematic analysis approach was adopted to extract insights from the feedback provided by medical students. | The results indicates that medical students in the experiment group experienced significant improvements in their well-being and life satisfaction. There was also a significant decrease in psychological distress and academic burnout. Furthermore, most students in the experiment group expressed satisfaction with the Chinese Student Mental Health Education Curriculum (CSMHEC), and the thematic analysis revealed valuable insights on how the course could be enhanced. | The findings suggest that integrating a mental health education course like CSMHEC into the medical curriculum can have a positive impact on the psychological well-being of medical students. However, it's important to note that more research is necessary to refine and enhance the design of such courses for use in medical education. This ongoing research can help tailor these programs to better address the unique needs and challenges faced by medical students, ultimately promoting their mental health and overall well-being. |
| Talisman et al. 2015 | Cross-Sectional Study | The impact of mind-body medicine facilitation on affirming and enhancing professional identity in health care professions faculty | 14 | 18 | The aim of this study was to determine the impact of mind-body medicine facilitation on affirming and enhancing professional identity in health care professions faculty. | To investigate whether Mindfulness-Based Medicine (MBM) facilitation has an impact on professional identity, self-awareness, and perceived stress, a group of 62 facilitators, who had received training from the GUSOM MBM program, were asked to participate in this study. They were requested to complete two well-established surveys: the Freiburg Mindfulness Inventory (FMI) and the Perceived Stress Scale (PSS). Additionally, 42 of the participants provided responses to a six-item open-ended questionnaire that delved into their experiences within the context of their professional identity. | The facilitators' scores differed significantly from normative controls, showing lower scores on the PSS and higher scores on the FMI (with a significance level of P < .05). Additionally, there was an inverse correlation (-0.46, P < .01) between these two parameters. Qualitative analysis of the data identified three main themes: 1. Aspects related to their professional identity, which encompassed subthemes such as communication skills, building connections and community, demonstrating empathy and active listening, and fostering self-confidence.2. Emphasis on self-care. 3. Promotion of mindful awareness. | The initial results will be expanded upon through more extensive studies. These larger studies will focus on conducting longitudinal quantitative assessments to examine how communication, connection, and self-confidence evolve over time in MBM facilitators. Additionally, these studies will investigate the effects of MBM facilitation on burnout and resilience. |
| Dyrbye et al. 2019 | Cross-Sectional Study | Medical School Strategies to Address Student Well-Being: A National Survey | 7 | NA | The aim of this study was to provide an overview of the various approaches employed by medical schools in the United States to enhance the well-being of their medical students. | In October 2016, a survey was conducted, involving 32 medical schools in the United States. The survey aimed to gather information regarding these schools' initiatives, resources, and infrastructure dedicated to student well-being, their grading practices in preclinical courses, as well as the presence of learning communities. | 84% of schools responded. 59% of these schools had integrated a curriculum focused on student well-being. These activities were typically scheduled during regular class hours, with about 81% of them occurring monthly. Attendance for these sessions was a mix of optional and mandatory. The schools offered a diverse range of activities promoting emotional/spiritual, physical, financial, and social well-being. Evaluation methods predominantly relied on participation rates and student satisfaction. Common practices included having a dedicated individual overseeing student well-being, forming student well-being committees, implementing pass/fail grading in preclinical courses, and fostering learning communities. | Numerous educational institutions have integrated a wide array of well-being programs and activities designed to promote self-care, reduce stress levels, and cultivate social support systems for medical students. However, these efforts vary in terms of the resources they have at their disposal, the infrastructure in place, and how they evaluate their effectiveness. To ensure that time and resources are utilized effectively and to gauge the true impact of well-being strategies, it is recommended to introduce specific well-being competencies and conduct rigorous assessments. Improving the evaluation process represents a vital next stage in mitigating student distress and ultimately enhancing the well-being of students. |
| Redwood et al. 2007 | Quasi-Experimental study | Student-led stress management program for first-year medical students. Teaching and learning in medicine | 10 | NA | The aim of this study was to evaluate a student-led stress management program for first year medical students. | The Stress Management Program is a voluntary program in which first-year medical students participate in small groups, guided by second-year student leaders. These groups convene for one hour each week, consistently for seven weeks at the start of the fall semester. The program is supervised by two faculty members who are psychologists and take on the roles of program coordinators. | For 16 consecutive years, an average of 94% of first-year students actively engaged in the program, demonstrating remarkable participation levels. Furthermore, the assessments of the program consistently revealed exceptionally favorable outcomes. | Considering its enduring existence, high levels of student engagement, and favorable student responses, the program has proven to be a success. Moreover, the document includes guidance on how to establish and uphold programs of a similar nature. |
| Daya et al. 2017 | Systematic Review | Mindfulness interventions in medical education: A systematic review of their impact on medical student stress, depression, fatigue and burnout | NA | NA | The aim of this study was to determine impact of mindfulness interventions on medical student stress, depression, fatigue and burnout. | In the process of conducting a search, a research protocol was employed, utilizing online databases including Embase, PubMed, PsycINFO, and MEDLINE. Articles were considered for inclusion if they met the following criteria: (1) they provided a description of a Mindfulness-Based Intervention (MBI) or incorporated mindfulness exercises as part of an intervention, (2) they evaluated at least one of the following outcomes: stress, burnout, fatigue, or depression, (3) they reported quantitative results, and (4) they had been published in peer-reviewed journals and written in the English language. | A total of twelve articles were included to a review process, with seven of them demonstrating positive developments in at least one of the targeted outcomes. Out of the seven studies investigating stress, four revealed favorable alterations, while in the case of depression, five articles reported decreases. Specifically, one study addressing burnout noted a decline in one of its components. However, when it came to fatigue, one study did not observe any significant changes. It's worth noting that approximately half of the studies in the review predominantly featured female participants. | The results of the study provided a combination of positive and inconclusive findings regarding the utility of Mindfulness-Based Interventions (MBIs) in diminishing psychological distress in undergraduate medical students. Future research should prioritize the investigation of mindfulness's impact on burnout and fatigue and assess whether the observed positive effects can be consistently replicated among male medical students |
| Ponciano et al. 203 | Quasi-Experimental Study | Effectiveness evaluation of online Mindfulness in mental health and alcohol consumption in medical students during COVID-19 pandemic | 12.5 | NA | To evaluate the effectiveness of an online mindfulness-based intervention on stress, anxiety, depression and alcohol consumption in medical students | Quasi-experimental study was performed with 237 students. Psychopathology, alcohol consumption, mindfulness was measured pre- and post-intervention through DASS-21, AUDIT, and MASS. | There was no significant reduction in alcohol consumption or psychopathologies. Mindfulness also did not improve | Further research involving generation strategies where students are involved in and complete intervention programs are recommended. This will help enhance future online mindfulness programs. |
| Da Silva et al. 2023 | Systematic Review and Meta-Analysis | Effectiveness of training programs based on mindfulness in reducing psychological distress and promoting well-being in medical students: a systematic review and meta-analysis | NA | NA | To evaluate the effectiveness of mindfulness 2based training programs in reducing psychological distress and promoting well-being of medical students | RCTs published until March 2022 without time or language restriction were searched on Cochrane library, Embase, PubMed/MEDLINE, PsycINFO/PsycNet, LILACS/BVS, ERIC (ProQuest), Web of Science, OpenGrey, and Google Scholar. | 8 articles met the inclusion criteria and were included. There is good quality evidence that mindfulness training improved the following outcome assessments: mindfulness, stress, psychological wellbeing/health. The quality of evidence for improvement in anxiety, depression, resilience and empathy was very low. | Mindfulness based training improved mindfulness, psychological health, and stress. |
| Sakura Horiuchi et al. 2022 | Literature review | Current Approaches to Yoga in U.S. Medical Schools: Scoping Review of the Literature | NA | NA | To evaluate if US medical schools offer accessible yoga to medical students and the details of these yoga. programmes | A scoping literature review was conducted with terms including "medical school," "medical student," "medical education," "yoga," "asana," "pranayama," and "mindfulness." | Yoga is offered in schools for recreational, research and educational purposes. There are various positive impacts on medical students ranging from scores on medical knowledge assessments, perceived stress and psychological states. | More opportunities should be created to engage medical students in yoga throughout their training. |
| Teresa Fazia et al. 2023 | Randomised Control Trial | Improving stress management, anxiety, and mental well-being in medical students through an online Mindfulness-Based Intervention: a randomized study | 13.5 | NA | To evaluate the effectiveness of MBI in improving medical student’s personal wellbeing, reducing anxiety, emotional discomfort and stress | The intervention consisted of ten twice-a-week Integral Meditation classes, dietary guidance, and short yoga sessions. Two groups of medical students from Italian universities: With each group having a control and intervention. Nine questionnaires were used, including stress (PSS), state anxiety (STAIX-1), well-being (WEMWBS), mind-wandering (MW-S), overall distress (PANAS), emotion regulation (DERS), resilience (RS-14), and attentional control (ACS-C and ACS-D). These were administered pre and post intervention. | MBI was effective in reducing perceived stress, improving mental well-bring and emotional regulation, resilience, and reducing the tendency to wander with the mind, improving the ability to maintain attention and improves overall distress | The results obtained shows an overall improvement in wellbeing at the end of the treatment suggesting that mindfulness is effective. |
| Hathaisaard et al. 2023 | Systematic Review and Meta-analysis | Mindfulness-based interventions reducing and preventing stress and burnout in medical students: A systematic review and meta-analysis | NA | NA | The aim of the study was to obtain more reliable outcomes and summarise specific interventions that effectively reduce stress and burnout of medical students. | A search of medical databases such as Embase, Ovid and CINAHL was performed. Only randomised control trials involving mindfulness-based interventions for medical students were accepted. | Six studies involving a total of 689 participants were included. The stress measurement scores for participants in the mindfulness-based interventions were significantly better than those in the control groups immediately after the intervention.  Furthermore, at the 6-month follow-up, the groups continued to exhibit significantly better outcomes. | Mindfulness groups have significantly better results than the control. MBI is effective in reducing stress both in the short term and long term. |
| Nemati et al. 2023 | Quasi-Experimental Study | Mindfulness-based resilience training on the psychological well-being of medical students during the COVID-19 pandemic | 15 | NA | To assess the effect of a mindfulness-based resilience training program on the psychological well-being of medical students during COVID-19. | 30 students who were chosen randomly from the population of medical students at Tabriz University of Medical Sciences during the academic year 2021-2022. They were divided randomly into two groups, with each group consisting of 15 participants. To evaluate the participants' psychological well-being, the researchers utilized Ryff's Psychological Well-Being Questionnaire (PWB-18). Additionally, as part of the study, an integrated training program focusing on both resilience and mindfulness was administered to the participants. | The results of the study demonstrated that the integrated training program, which combined elements of resilience and mindfulness, had a positive impact on the psychological well-being of the students. Moreover, this intervention was able to account for 59% of the combined variation observed in the psychological well-being variables among the participants. | The current study's findings suggest that an integrated training program involving both resilience and mindfulness can lead to an enhancement in individuals' psychological well-being, particularly in challenging circumstances like the COVID-19 pandemic. |
| Augustine George et al. 2023 | Quasi-Experimental Study | The effect of autogenic training, mental imagery, mindfulness meditation and pranayama on zoom exhaustion and fatigue among medical students | 11 | NA | To assess the impact of relaxation techniques on reducing Zoom exhaustion. | Pre and post-test Zoom Exhaustion and Fatigue Scale were administered. Post-test was administered on a particular day after 6h of online classes, after which students were made to do their chosen relaxation technique. Students in experimental groups were motivated to practice chosen relaxation technique for the next 7 days for 5mins a day. After 7 days, the post-test was once again administered. | All interventions proved to be effective reducing exhaustion and fatigue with autogenic training producing the most promising results. | This study demonstrates the prevalence of Zoom fatigue and the impact of intervention programs such as autogenic training, mental imagery, mindfulness meditation, and bhramari pranayama on alleviating this fatigue. The results y revealed that while all of these relaxation techniques had a positive effect, autogenic training emerged as the most effective in significantly reducing Zoom fatigue among the participants. |
| Xinyi Dai et al. 2023 | Quasi-Experimental Study | Analysis of mental health status before and after psychological intervention in response to public health emergencies by medical students: a prospective single-arm clinical trial | 14 | NA | To determine the impact of psychological interventions on student’s mental health in response to public health emergencies. | 121 third-year medical undergraduate students from participated voluntarily in interest groups and received psychological interventions over the course of one year. To assess their mental well-being, various scales were used, including Zung's Self-rating Anxiety Scale (SAS), Self-rating Depression Scale (SDS), and Somatic Self-rating Scale (SSS), both before and after the interventions. Additionally, psychological coping styles were analysed using the trait coping style questionnaire (TCSQ). The students were evaluated every 3 months, with the initial survey results serving as baseline data and being compared to the final results.). | The findings indicated significant changes in symptoms, with percentages shifting from 25.62% to 7.44% for anxiety, 28.93% to 18.18% for depression, and 21.49% to 9.92% for somatization (all P<0.05). Additionally, participants exhibited noticeable alterations in their coping styles. Specifically, positive coping style scores increased from, while negative coping style scores decreased following the intervention These results suggest that psychological interventions had a positive impact on the mental well-being and coping strategies of individuals during the pandemic. | Implementing psychological interventions within interest groups, coupled with individual mental health counselling, can be an effective approach to enhance the mental well-being of college students. This combined strategy has been shown to have a positive impact on reducing anxiety levels among students, demonstrating the potential benefits of comprehensive mental health support during such challenging times. |
| Nielsen et al. 2023 | Quasi-Experimental Study | A student-driven mindfulness curriculum for first- year osteopathic medical students: a pilot study | 13 | NA | To determine the student satisfaction with our student selected and student led mindfulness activates incorporated into compulsory small group sessions and use of these activities outside mindfulness sessions. | First-year medical students participated voluntarily in mindfulness sessions led by fellow students. These sessions occurred once a week for eight consecutive weeks during regular class time. The mindfulness activities included various techniques such as yoga postures, the 4-7-8 breathing technique, progressive muscle relaxation (PMR), and values affirmation. within the 8-week period. Assessment included a survey aimed to assess their participation, any changes in stress levels, their satisfaction with the activity, and whether they practiced mindfulness activities outside of the sessions. | A range of 14 (9.1%) to 94 (61.0%) students actively engaged in the weekly mindfulness activities. It was noted that the 4-7-8 breathing technique was the most commonly practiced mindfulness activity outside the sessions, with 32.3% of students indicating they practiced it across all weeks. The mindfulness activity with the highest percentage of reported stress reduction was yoga postures during week 5, with 94.8% (reporting a decrease in stress. Moreover, both weeks featuring yoga activities had the highest student satisfaction rates, with 95.7% for week 1 and 92.1% (35 out of 38 students) for week 5. These findings indicate the potential benefits of these mindfulness activities in reducing stress and promoting well-being among medical students. | The results indicates that student-selected and student-led mindfulness activities have the potential to effectively reduce stress among medical students who actively engage in them. However, further research is necessary to better understand how to optimize the implementation of mindfulness curricula in medical education. There is room for improvement in how they are integrated into medical school programs to maximize their effectiveness in promoting student well-being. |
| Neufeld et al. 2023 | Editorial | Towards an autonomy-supportive model of wellness in Canadian medical education | NA | NA | To demonstrate the problems with creating an individual-focused 'wellness' intervention | Based on solid theoretical foundations and supported by empirical evidence, the primary contention put forth in this commentary is that (IFWs) are unsuitable, offensive, and have detrimental psychological effects on learners. Therefore, the author asserts that these involuntary flashbacks should be discontinued or eliminated. | Building upon previous research in this domain, the author begins by outlining three core issues associated with (IFWs). Subsequently, they propose a shift in the prevailing approach to addressing "wellness" within the context of medical education. | In conclusion, the author furnishes an evidence-based plan, rooted in self-determination theory, detailing how systemic enhancements can be implemented in a timely, enduring, and socially responsible manner. This plan is designed to yield benefits across the entire medical spectrum, encompassing leaders, educators, learners, and patients alike. |
| Rojas et al.  2023 | Randomised Control Trial | A compassion-based program to reduce psychological distress in medical students: A pilot randomized clinical trial | 13 | NA | To determine the effectiveness of a compassion-based program in reducing psychological distress in medical students | Medical students were assigned randomly to one of two groups: the Compassion Cultivation Training (CCT) group or the Waitlist control group (WL). They were required to complete self-report assessments at three different time points: before the intervention, immediately after the 8-week intervention, and again at a 2-month follow-up. The study aimed to assess various outcomes including compassion, empathy, mindfulness, well-being, resilience, emotional regulation, psychological distress, burnout, and concerns related to COVID-19. | In comparison to the Waitlist (WL) group, the Compassion Cultivation Training (CCT) group demonstrated substantial enhancements in several key areas. These improvements included increased levels of self-compassion, mindfulness, and emotion regulation. Additionally, there was a significant decrease in stress, anxiety, and the emotional exhaustion component of burnout among CCT participants. Importantly, some of these positive effects persisted even at the follow-up assessment, suggesting a lasting impact of the intervention. Importantly, no adverse effects associated with meditation practices were observed in the study. | Compassion Cultivation Training (CCT) has been shown to be effective in improving compassion skills and reducing psychological distress among medical students. This is particularly crucial for maintaining the mental well-being of future physicians while also fostering a culture of compassionate care for patients. The study underscores the importance of institutions considering the inclusion of this type of training in their medical education programs, as it can have significant positive implications for both medical students and the quality of care provided to patients. |
| Nader et al. 2023 | Prospective Study | A Larger Lens: Medical Students Benefit from Consciousness-Based Self-Care | NA | 21 | To determine the impact of transcendental meditation on medical students | A major medical school in the United States took a proactive step to tackle burnout and promote well-being among its students by introducing a 2-credit elective course centred around the Transcendental Meditation® (TM®) technique. This chapter presents findings from an extensive 8-year study that investigated the experiences of medical students who participated in this course. | The study findings indicate three significant benefits resulting from the intervention 1. Reduction of stress and anxiety  2. Increase in compassion, empathy and wellbeing 3. Enhanced Productivity and Interpersonal Relationships | Research results indicate that the TM technique may have significant potential as a core element in wellness programs for medical students and for the broader wellness industry as a whole. |
| Qureshi et al. 2023 | Cross-Sectional Study | Counselling of Medical Students a Supportive Tool | 12 | NA | To determine the effect of counselling and guidance on the academic performance of medical students through student perceptions | Students were divided into two groups, labelled as Group A and Group B, based on their academic performance during the 2021-2022 academic year in both professional and module examinations. 13 questions that were aimed to assess the students' perceptions regarding the impact of counselling and guidance on various factors influencing their academic performance were distributed to both groups. Group A included high-performing and high-achieving students, while Group B comprised students with average academic performance and those who had failed in their exams. | Most students from both groups expressed positive views regarding the effectiveness of faculty counselling and guidance across various aspects of their academic journey, including managing the demanding medical study schedules, preparing for professional exams, time management, dealing with social and domestic challenges, addressing attendance issues, and enhancing their overall academic performance. These results suggest that students with strong academic performance, as well as those with average to low academic performance, acknowledged and valued the support and guidance provided by faculty members in their academic and career pursuits. | Faculty members play a significant role in addressing both academic and non-academic challenges faced by students. The research findings highlight that students have acknowledged the valuable support provided by faculty members in enhancing their academic performance through guidance and counselling. This recognition underscores the importance of faculty involvement in student well-being and success. |
| Mugford et al. 2022 | Prospective Study | Medical Students’ Perceptions and Retention of Skills From Active Resilience Training | 10 | NA | To determine the long-term utilisation of learning resiliency skills | Medical students were surveyed approximately 1 to 18 months after undergoing Active Resilience Training (ART). The survey, conducted electronically, aimed to evaluate the program's effectiveness in achieving its intended goals and to gauge the frequency with which students applied the skills they had acquired during the training. | The results indicate that Active Resilience Training (ART) is highly successful in raising awareness about the advantages of resilience training. A significant majority of participants expressed their willingness to recommend the course to their fellow students. Moreover, students continued to apply the skills they acquired for an extended period, lasting more than 18 months after completing the training. These skills encompassed taking planned breaks, prioritizing sleep, establishing support networks, and practicing mindfulness techniques. | This study contributes to the existing body of literature by examining how participants value innovative resilience curricula. It highlights that students continued to apply the skills acquired through Active Resilience Training (ART) for up to 18 months after program completion. There is a need for further research to assess the precise impact of ART on traditional performance measures. |
| Qasim et al. 2022 | Cross-Sectional Study | Mindfulness and Psychological Distress in Medical Students: Mediating Role of Emotion Regulation | 13 | NA | The research aimed to investigate whether emotion regulation serves as a mediating factor in the relationship between mindfulness and psychological distress, which includes conditions like depression, anxiety, and stress. | A correlation research design was employed, and data were gathered using purposive sampling from both public and private medical colleges. a total of 216 participants, consisting of 96 men and 120 women were included. Assessment tools used were the Mindful Attention Awareness Scale 3, the Emotion Regulation Questionnaire, and the DASS 21 (Depression, Anxiety, and Stress Scale). Additionally, a demographic information sheet was used to collect relevant background information from the participants. Data analysis was conducted using SPSS 21. | The study's results indicated that there were significant relationships among all the variables examined. Specifically, it was found that emotion regulation played a mediating role between mindfulness and psychological distress, particularly in relation to anxiety. This suggests that the practice of mindfulness may influence psychological distress levels, specifically anxiety, through its impact on how individuals regulate their emotions. | This study's findings assist clinical psychologists in developing more effective training programs and therapeutic approaches for medical students. These interventions aim to reduce distress among medical students, ultimately enhancing their academic performance. |
| Neufeld et al. 2022 | Cross-Sectional Study | Need Fulfilment and Resilience Mediate the Relationship between Mindfulness and Coping in Medical Students | 11 | NA | The objective is to assess how meeting the fundamental psychological needs (autonomy, competence, and relatedness) of medical students influences their ability to practice mindfulness and effectively handle stress. This is in the context of Self-Determination Theory. | A total of 197 medical students from the University of Saskatchewan took part in this research, comprising 71 first-year students, 58 second-year students, and 36 third and fourth-year students. These students completed an anonymous survey that gauged their satisfaction with their psychological needs within the medical school environment, their level of mindfulness, resilience, and their frequency of employing different coping strategies, both adaptive and maladaptive. The study aimed to investigate whether the fulfilment of needs and resilience played a role in mediating the connection between mindfulness and coping styles. | The study found that the satisfaction of psychological needs and resilience played a complete mediating role in the link between mindfulness and adaptive coping. On the other hand, when it came to maladaptive coping, the mediating role of need frustration and resilience was partial. Additionally, it was observed that need fulfilment had a more significant mediating effect than resilience in both coping models. | In conclusion, the results indicate that providing greater support for the resilience and fundamental psychological needs of medical students could enhance their mindfulness. Consequently, this improved mindfulness may enable them to respond more effectively to the challenges and stressors encountered during their medical education, leading to more adaptive coping and a reduced likelihood of maladaptive coping strategies. |
| Qasim et al. 2022 | Cross-Sectional Study | Predictive Role of Mindfulness and Emotion Regulation for Psychological Distress in Pakistani Medical Students | 12 | NA | The purpose of this study was to explore the connections among mindfulness, emotion regulation, and psychological distress in medical students as well as to determine whether mindfulness and emotion regulation could predict levels of psychological distress. | The research utilized a correlational research design and employed purposive sampling techniques. Data were collected from medical colleges, including both public and private institutions. The total sample consisted of 216 participants, with 96 men and 120 women. Various tools were employed, including the Mindful Attention Awareness Scale, Emotion Regulation Questionnaire, and the Depression, Anxiety, and Stress Scale (DASS-21), in addition to a demographic information sheet, to measure the study variables. The collected data were analysed using SPSS version 21. | The study's findings indicated a noteworthy inverse association between mindfulness and emotion regulation on one hand and psychological distress (comprising depression, anxiety, and stress) among medical students on the other. Additionally, the study revealed that both mindfulness and emotion regulation significantly predicted levels of psychological distress, particularly in the case of anxiety. | The outcomes of this study have the potential to assist clinical psychologists in the development of more effective training programs and therapeutic interventions tailored for medical students. The primary goal is to reduce the distress experienced by these students, ultimately enhancing their academic performance. |
| Noorily et al. 2023 | Quasi-Experimental Study | The art of seeing: The impact of a visual arts course on medical student wellbeing | 12 | NA | To determine impact of a visual arts course on medical student wellbeing. | In this study, a total of 40 students took part between 2019 and 2021. Among them, 15 students were enrolled in the pre-pandemic, in-person course, while 25 students participated in the post-pandemic, virtual course. The study involved both pre and post-tests, which consisted of open-ended responses to artistic works that were subsequently analysed for themes. Additionally, standardized scales such as the Mindful Attention Awareness Scale (MAAS), Situational Self-Awareness Scale (SSAS), and Perceived Stress Questionnaire (PSQ) were utilized as assessment tools. | The study found statistically significant improvements among students in three key areas: the MAAS (Mindful Attention Awareness Scale) with a p-value of less than 0.01, the SSAS (Situational Self-Awareness Scale) also with a p-value of less than 0.01, and the PSQ (Perceived Stress Questionnaire) with a p-value of 0.046. Notably, improvements in the MAAS and SSAS were independent of the class format. Furthermore, the post-test free responses from students revealed increased focus on the present moment, heightened emotional awareness, and greater creative expression compared to their pre-test responses. | The course had a significant positive impact on mindfulness, self-awareness, and stress levels among medical students. These findings suggest that the course can be a valuable tool for enhancing well-being and addressing burnout in this population, whether it is delivered in-person or virtually. |
| Oláh et al. 2022 | Cross-Sectional Study | Transfer of Mental Health Services for Medical Students to Cyberspace during the COVID-19 Pandemic: Service Use and Students’ Preferences for Psychological Self-Help Techniques | 11 | NA | Our objective was to gain insight into the preferences of medical students regarding psychological self-help techniques. We aimed to achieve this by examining how they accessed online self-help resources and by studying the characteristics of the individuals who utilized these materials. | We analysed access to online materials from April 2020 to April 2021 among both Hungarian and international medical students. This analysis was conducted using the system's logging data. | Among all the students who logged in during the examination period (n = 458), approximately 36.6% to 40.4% accessed materials aimed at enhancing their study skills, while 23% to 29% viewed stress management materials. Notably, shorter audio-based techniques were the preferred format for stress management. The access rate for content related to coping with the mental health effects of COVID-19 ranged from 9.5% to 24%. Interestingly, there was a significant preference for support materials designed to improve study skills compared to interventions focused on reducing distress. | Among all the students who logged in during the examination period (n = 458), approximately 36.6% to 40.4% accessed materials aimed at enhancing their study skills, while 23% to 29% viewed stress management materials. Notably, shorter audio-based techniques were the preferred format for stress management. The access rate for content related to coping with the mental health effects of COVID-19 ranged from 9.5% to 24%. Interestingly, there was a significant preference for support materials designed to improve study skills compared to interventions focused on reducing distress. |
| Nguyen et al. 2023 | Quasi-Experimental Study | Transforming stress program on medical students’ stress mindset and coping strategies: a quasi-experimental study | 13 | NA | The aim of this study was to assess the impact of the Transforming Stress Program (TSP) on stress mindset and coping strategies in first-year medical students when they encounter stressors. | The study involved a total of 409 first-year students, divided into two groups: the intervention consisting of 205 students and the control consisting of 204 students. The study implemented a 10-week Transforming Stress Program (TSP) delivered as an extra-curricular course. This program was designed with a foundation in psychoeducation, drawing from Dialectical Behavioural Therapy principles, and incorporated mindfulness as a core practice in each component. The intervention group received the TSP training during the first semester, while the control group received an identical program during the second semester assess the effects of the program, measurements for Stress Mindset and Brief Coping Orientation to Problems Experienced were taken at three points in time: before the intervention (T0), immediately after the intervention for the intervention group (T1), and six months after the intervention for the intervention group (T2). | At T1, the intervention group displayed substantial improvements, with a 65% increase in stress mindset scores and higher scores in six coping strategy domains (Problem solving, social support, Humour, Religion, Venting, and Self-distraction). Additionally, there were decreases in three coping strategy domains (Avoidance, Substance use, and Self-blame). These changes were all statistically significant, indicating substantial impact. In contrast, the control group did not exhibit significant changes during the same period. By T2, the effects of the program had somewhat diminished in certain coping strategy domains (Avoidance, Substance use, and Self-blame). However, these effects remained significantly better than the baseline measurements at T0, indicating that the program had a lasting positive impact on the intervention group's stress mindset and coping strategies. | The Transforming Stress Program (TSP) has proven to be both feasible and effective, leading to significant improvements in stress mindset and coping strategies among medical students. Notably, the positive effects of the program were still evident six months after its completion. It's worth noting that this relatively intensive intervention requires the support and collaboration of the school administration and staff to be successfully implemented. |
| Abrams et al. 2022 | Quasi-Experimental Study | Impact of Providing Peer Support on Medical Students’ Empathy, Self-Efficacy, and Mental Health Stigma | 12.5 | NA | The aim of this study was to determine the impact of providing support to students in a medical school peer support program. | Ap pre-post, quasi-experimental approach was used to assess the impact of volunteering as peer supporters on medical students in their second through fourth years of medical school. They employed four measures: (1) empathy, (2) self-efficacy, (3) mental health stigma, and (4) willingness to assist peers dealing with mental health issues. The study involved 38 actively enrolled medical students serving as peer supporters during the 2020–2021 academic year at a U.S. allopathic medical school. The study's results are not provided in the provided text. | The results of the study indicated that medical students who served as peer supporters experienced positive changes in certain areas. Specifically, they demonstrated higher levels of empathy, as evidenced by a statistically significant increase in empathy scores and increased self-efficacy, supported by a statistically significant rise in self-efficacy scores following their participation in the program. However, there were no significant alterations observed in terms of mental health stigma or their willingness to assist peers facing mental health issues. | Peer-support programs offer an affordable and sustainable approach to enhancing the well-being of medical students. This study revealed that peer supports reported increased levels of empathy and self-efficacy. These results emphasize the significance of peer-support programs, not only benefiting the medical students utilizing the services but also those who volunteer as peer supporters. |
| Barsa et al. 2023 | Editorial | Medical school and mental health: Our student perspective | NA | NA | NA | NA | NA | NA |
| Boyd et al. 2022 | Randomised Control Trial | An online mindfulness intervention for medical students in South Africa: A randomised controlled trial | 13 | 20 | To determine the effectiveness of an online mindfulness intervention for medical students | In this study, a total of 45 participants were randomly divided into two 6-week groups, which were led by teachers. The research utilized a repeated measures analysis of variance (to examine the changes in outcome measures, including well-being, perceived stress, and self-compassion scores, at three different time points. Additionally, qualitative data was collected through thematic analysis of participant feedback, providing both quantitative and qualitative insights into the study's findings. | Both groups of participants displayed improvements in measures of well-being, perceived stress, and subjective stress management as the study progressed. Moreover, participants in the mindfulness group exhibited a statistically significant treatment effect in mindfulness by the end of the program. Interestingly, a decrease in self-compassion was observed in both groups as time went on, indicating a notable trend in this aspect. | Within the South African medical student group, both an online Mindfulness-Based Intervention (MBI) and a Stress Coping (SC) program are viable options and demonstrate promise in reducing stress levels, enhancing stress management abilities, and boosting resilience. It is advisable to conduct further research to gain a deeper understanding of these interventions' effectiveness and potential benefits. |
| Brami et al.  2022 | Cross-Sectional Study | Mindfulness training, a learning process towards three directions: oneself, others, the academic system in medical education: a qualitative study | 17 | NA | The aim of this study was to examine the real-life experiences of medical students immediately after they had completed a voluntary 8-week Mindfulness-Based Stress Reduction (MBSR) program. | The study involved conducting semi-structured face-to-face interviews with a total of eighteen medical students, ranging from those in the early stages of their medical education to those in residency. The collected interview transcripts were subsequently analysed using the Interpretative Phenomenological Analysis (IPA) method to gain insights into the participants' experiences. | 7 themes were identified and grouped into 3 overarching themes.  1. Theme of Self (Oneself): Seen as having a healing dimension, leading to behavioural changes and improved emotional regulation.  2. Theme of Others: A new way of listening and experiencing empathy as a result of mindfulness training  3. Theme of the Academic System The importance of university support in fostering a caring environment. Such support was seen as a crucial factor in building confidence among participants. | The MBSR program emerged as a valuable pedagogical tool for teaching self-care skills and fostering a sense of care for others within medical education. It was recognized as a multidimensional learning process. Future research is needed to better understand the specific process variables and potential risk factors involved. |
| Brami et al.  2023 | Cross-Sectional Study | Understanding students’ motivations for participating in a mindfulness course: a qualitative analysis of medical students’ views | 21 | NA | The aim of this study was to evaluate student's motivation for participating in a mindfulness course | The study involved the analysis of 29 transcripts from the initial session of an 8-week Mindfulness-Based Stress Reduction (MBSR) program, which was conducted in French for medical students. The transcripts underwent coding and analysis through a qualitative content thematic analysis approach, alongside the constant comparison method to identify patterns and themes within the data. | The analysis revealed three prominent themes that show the motivations of the medical students: Theme of Medical Education and the Physician's Role: Students were motivated to improve their interpersonal skills, acquire skills geared toward a more integrative approach to medicine, and increase their productivity in a highly competitive academic and professional environment. Theme of Caring for One's Health): Here, the students expressed a motivation to prioritize their own well-being. This involved seeking ways to reduce stress, regulate their emotions, and enhance self-compassion. Theme of a Quest for Meaning: The third theme centred on a profound quest for meaning. | The findings of this study emphasize the alignment between the motivations expressed by the participants and the existing evidence regarding the impact of mindfulness. This alignment is particularly evident in how mindfulness can facilitate self-care, foster the development of humanistic medical skills, and deepen the understanding of the meaning of care. |
| Brigg et al.  2022 | Cross-Sectional Study | Combatting Burnout by Maximizing Medical Student Participation in Exercise Events | 9 | NA | To explore how participation in exercise can combat burnout in medical students | Students from nine different medical schools in the United States were asked to participate in an online survey. This survey aimed to evaluate the effectiveness of various factors in encouraging and increasing participation in exercise events among the students. | Regarding factors that were considered most likely to increase participation: 56% of the students reported that financial discounts were a significant factor. 46% mentioned that having all logistics planned by event organizers was important. 40% indicated that opportunities to find informal mentors were likely to increase their participation. | To boost student engagement in wellness events, educational institutions should allocate their available funds strategically. The focus should be on initiatives that both save students time and money while fostering a sense of community across different levels of training and academic departments. |
| Brown et al. 2022 | Quasi-Experimental Study | Can stoic training develop medical student empathy and resilience? A mixed-methods study | 12.5 | 26 | To determine if stoic training can develop medical students’ empathy and resilience. | A study involving 24 third-year medical students was conducted to assess the impact of a 12-day online training program called SeRenE, which was based on Stoic philosophy and developed in collaboration with psychotherapists. The study used a mixed-methods approach to evaluate the program's effects. . | There were increases in Stoic ideation, resilience, and empathy among the participants. Correlational analyses indicated that improvements in resilience and empathy were linked, suggesting that as one increased, the other tended to increase as well. Qualitatively, the study identified the following themes: Negative Visualization for Preparedness, Stoic Mindfulness and Self-awareness, Stoic Reflection and Empathic Imagination, Evaluating the Accessibility of Serene. | In summary, our research suggests that Stoic-based psychological training can be a powerful tool for enhancing both resilience and empathy among individuals, with a particular emphasis on the positive effects of practices like negative visualization and their implications for academic success and empathic growth. |
| Butt et al. 2022 | Quasi-Experimental Study | Effectiveness of Mindfulness Based Cognitive Therapy in Reducing Stress among Senior Medical Students: A Single Subject Experimental Study | 12.5 | NA | To determine the efficacy of a mindfulness based cognitive therapy in reducing stress among senior medical students. | A research study was conducted using a single subject experimental design with multiple baselines on a group of five medical students To assess stress levels, the researchers employed the Medical Student Stress Questionnaire (MSSQ), while the levels of mindfulness were measured using the Mindfulness Attention Awareness Scale (MAAS). | The study's findings supported the initial hypothesis, as all participants exhibited a reduction in their stress levels and an improvement in their mindfulness. This suggests that Mindfulness-Based Cognitive Therapy (MBCT) was effective in helping medical students manage stress. | In summary, the study underscored the efficacy of Mindfulness-Based Cognitive Therapy in addressing stress. Furthermore, the results indicate the potential for exploring the application of MBCT in treating various other psychological symptoms and issues. |
| Villalón et al. 2023 | Randomised Control trial | Brief Online Mindfulness‐ and Compassion‐Based Inter‐Care Program for Students During COVID‐19 Pandemic: A Randomized Controlled Trial | 14.5 | NA | The aim of this study was to determine the effectiveness of a brief online mindfulness and compassion based inter-case intervention for medical students. | A randomized controlled trial was conducted at a Chilean university with 360 medical students. The study assessed their well-being, anxiety, and depression symptoms at three time points: at the beginning of the trial, one month later, and three months later. Intervention group participants were divided into one group received a mindfulness-based inter-care intervention (IBAP) consisting of one-hour sessions per week over four weeks, with homework assignments; the other group received a psychoeducational intervention (PSE) also consisting of one-hour sessions per week over four weeks with homework assignments. An additional group of 240 students did not receive specific interventions and served as a control group, receiving only the general intervention (GI). | At the beginning of the study, both the IBAP and PSE groups had higher scores in depression symptoms compared to the TAU-GI group. Over the course of the study, significant reductions in depression symptoms and anxiety symptoms (were observed in both the IBAP and TAU-GI groups, but not in the PSE group at both the first and third months. Furthermore, when compared to the TAU-GI group, the IBAP group exhibited a noteworthy reduction in depression symptoms at the first-month assessment. Additionally, an analysis of secondary variables revealed improvements in the factors of mental health continuum and common humanity on the Self-Compassion Scale among the participants. | Based on our findings, it appears that a short online intervention focusing on mindfulness and compassion, along with academic flexibility and breaks, was effective in enhancing the mental health of medical students during the COVID-19 pandemic. |
| Volpe et al.  2022 | Quasi-Experimental Study | Can an Arts Course Help Mitigate Medical Student Burnout? | 12.5 | 26 | To determine the effectiveness of an art course in mitigating burnout in medical students. | Mixed methods study was used to evaluate if a 9-month program for 4th year medical students named Art as Self Care (AASC) would help them develop practices which support their wellbeing. Online survey was used to collect data pre and post intervention. Focus group was used as well. | Qualitative results suggest that the course acted as a beneficial distraction which is calming and enabled students to move their focus from their stress in life. The quantitative results suggest a possible protective effect for medical students, as there as less worsening of psychological distress across the fourth year as compared to the control group. | Our research provides initial and practical information to enhance future studies and approaches regarding the utilization of art to promote the well-being of medical students. |
| Worobetz et al. 2022 | Randomised Control Trial | Exercise Compared to Mindfulness for Physical and Mental Wellbeing in Medical Students | 14 | NA | To compare the effectiveness of Physical Activity (PA) and Mindfulness-Based stress Reduction (MBSR) in improving mental wellbeing of medical students. | 2nd year medical students were voluntarily assigned to intervention or control. Outcome assessment consisted of pre and post-test measure of overall health, wellbeing, sleep quality, loneliness, current level of PA and confidence in prescribing exercise as medicine. | Both methods showed statically significant improvement as compared to their controls. However, there was no statistically significant differences in changes in outcome measure between groups. | Both the 'Med-Well" programme and MBSR produced similar enhancements in overall wellbeing and sleep quality |
| Van Dijk et al. 2022 | Cross-Sectional Study | Experiences of clinical clerkship students with mindfulness-based stress reduction | NA | 26 | To investigate the long-term effects of mindfulness-based stress reduction on clinical clerkship students. | 2 years after participating in MBSR, training, students were interviewed and quired about their current mindfulness practice and the long terms effects of MSBR training. | The majority of students still engaged in regular, predominantly informal mindfulness practice, although a minority no longer practiced mindfulness and reported an unchanged lifestyle. | Most students still engaged in mindfulness 2-year post intervention, contributing to both personal and professional changes. Due to the high demands of clinical clerkships, MSBR training is a valuable addition to the medical curricula. |
| Ungar et al. 2022 | Systematic Review | Online programs to strengthen the mental health of medical students: A systematic review of the literature | NA | NA | The aim of this study was to review the literature and provide an overview on the online program used to strengthen the mental health of medical students | A systemic search of PubMed, Eric, Cochrane and web of science was performed. Inclusion criteria consisted of web-based program that targeted medical students. For outcome, it consisted of mental health, burnout, symptoms of depression, anxiety and wellbeing. | The search yielded 723 articles with 11 meeting the inclusion criteria. Programs found were divided into their focus, mental health literacy, mindfulness-based CBT or peer support. | Online programs provided a effective was to support individual mental health promotion and prevention by strengthening protective characteristics. |
| Wang et al. 2022 | Randomised Control trial | Randomized controlled pilot study of feasibility and effectiveness of peer led remote Mindfulness‐Based Art Workshops on stress, anxiety, and depression in medical students | 15 | NA | To determine the feasibility and effectiveness of a peer led remote mindfulness-based art workshop on stress anxiety and depression in medical students. | The intervention combined two established interventions, mindfulness-based stress reduction and art therapy. 24 students were randomised into intervention or control groups. Outcome assessment included short term change in State Trait Anxiety (STAI) and The National Institutes of Health (NIH) Toolbox Perceived Stress survey. | There was a reduction in short-term anxiety and perceived scores post intervention. | The program has proven to be effective in significantly reducing short-term anxiety. Furthermore, it also demonstrates a potential trend in decreasing perceived stress levels that can last for up to 2 weeks after the intervention, although these results did not reach statistical significance. To validate these promising outcomes, future research should encompass a larger and more diverse population, affirming the positive results observed in this initial study. |
| Smith et al. 2022 | Editorial | Wholehearted: Medical Students Embracing Vulnerability and Finding Fulfilment | NA | NA | To determine the effectiveness of a lifestyle medicine program in enhancing medial student wellbeing | A 12-week program created by 2 student leaders addresses 6 pillars of lifestyle medicine as well as positive psychology. The program was offered to all first-year students. It was held over zoom weekly for 1-2 hours | There was a high attendance rate. And 100 percent of the respondent reported that they would recommend this program to another medical students. 71 percent reported that they would use the tools taught weekly. Growth mindset, gratitude, mindfulness, priorities and relationship building were the most used- wellbeing strategies. | A Zoom-based intervention rooted in the principles of the 6 pillars of lifestyle medicine and positive psychology is both practical within the context of medical school and warmly embraced by medical students. |
| Kaisti et al. 2023 | Systematic Review | The effects of mindfulness‑based interventions in medical students: a systematic review | NA | NA | The aim of the study was to collate quantitative results of mindfulness-based interventions and analyse their outcomes and characteristics of interventions. | A search on the different databases was performed in June 2020. The inclusion criteria consist of: (1): at least 50% of the participants are medical students, (2) included a mindfulness intervention (3) analysed an outcome relating to the mindfulness intervention (4) peer reviewed (5) written in English. | 31 articles were included. Half of the studies consisted of RCTs. A majority of the interventions consisted of Mindfulness Based Stress Reduction or Mindfulness Based Cognitive Behavioural Therapy. The general satisfaction of the interventions was positive. Furthermore, the meta-analysis revealed that the interventions groups had significantly fewer symptoms of stress and distress, as well as higher mindfulness compared to controls. This beneficial effect also lasted for follow ups over months to years. | There has been a surge in the number studies on mindfulness-based interventions. It offers a good method to enhance the wellbeing of medical students. |
| Kelly et al. 2022 | Quasi Experimental Study | ‘Things we are expected to just do and deal with’: Using the medical humanities to encourage reflection on vulnerability and nurture clinical skills, collegiality, compassion, and selfcare | 7 | 11 | The aim of this study was to describe The Vulnerability in Medicine (ViM) program as well as investigate its beneficial outcomes. | Students were surveyed in a two-part survey. The first part allowed students to indicate their overall satisfaction with the course (very satisfied, satisfied, neutral, dissatisfied and very dissatisfied). The second part allowed for free text responses.  The Vulnerability in Medicine (ViM) program was developed to create safe spaces for medical students to reflect on challenges in patient-doctor relationships, professional development and to recognise vulnerability in themselves. | The study reported improvement in professional practice and academics due to a shift from a biomedical focus to a ‘whole person’ approach. Improvements in psychological symptoms and the ability to cope with negative emotions due to the creation of safe spaces and collegiality. | The program provided a safe space and is restorative for both students and clinicians. It also allows for engagement in creative enquiry. |
| Lee et al. 2022 | Randomised controlled study | Yoga as an adjunct activity for medical students learning anatomy | 10.5 | NA | The study aims to investigate if Hatha yoga synced with anatomy lectures would enhance learning of anatomy. | Yoga sessions were taught by certified 200-h yoga teachers trained in Alignment yoga. Each yoga session complemented the anatomy lecture topics for the week. Students in the yoga intervention cohort completed eight yoga sessions. Before and after each session, self-reported confidence in the anatomy content covered in that session and stress on an 11-point score scale was collected. DASS-21 assessment was also complete. Before each anatomy exam, PSS-4 was collected. | There was no significant improvement in performance during the anatomy exam or significant reduction in stress levels before each exam. However, there were significant lower stress levels after each yoga session. There was also reportedly increased confidence in the anatomy after yoga sessions which focused on the back, upper extremity, head and neck, the abdomen and pelvis. Participation rate was much lower in the second group during the spring semester (1/32 participated), this is hypothesized to be caused by the lack of a concomitant main curriculum (i.e. the anatomy course) which can benefit from these adjuncts. | The use of yoga as an adjunct to an anatomy course did not lead to improvement of anatomy test course. However, these adjuncts did not detract from study time and was still effective in decreasing stress in students. The study also highlights the desire for medical students for an adjunct which is complimentary to their main curriculum. The main limitations are the decreased survey response rates as sessions progressed which was addressed using the mixed-effects model. |
| Leena et al. 2022 | Cross-Sectional Observational Study | Impact of a mental health awareness program on knowledge, attitude, and perceptions about mental health disorders among medical students | 6 | NA | The aim of the study was to determine the impact of a mental awareness program on the knowledge, attitudes, and perceptions about mental health among medical students. | An online survey was conducted after the one-week long programme. The questionnaire contained questions to assess awareness and attitudes towards mental health. | 78.7% of respondents found the one-week programme helpful in promoting awareness of mental health. 65.7% of respondents had improved help seeking behaviour. 88% of respondents had an improvement in sense of wellbeing. Posters were the most impactful event within the programme which promoted awareness of mental health. | Mental health awareness programs successfully improved the sense of well-being of medical students and fostering a positive attitude towards mental health. There should be suitable curriculum changes to include such programmes. |
| Henry et al. 2023 | Systematic Review | Measuring wellbeing: A scoping review of metrics and studies measuring medical student wellbeing across multiple timepoints | NA | NA | The aim of this study was to investigate the metrics and methods used to measure medical student wellbeing at multiple time points. | A search of 5 databases were performed between May and June 2021, including studies using survey-based metrics among medical students at multiple time points. The screening and extraction of the data was completed by two reviewers. | A total of 221 studies were included with 109 being observational and 112 being intervention based. 140 unique metrics measuring 13 different constructs were discovered. The most prevalent intervention method was stress management. | There is a need for further research to address the challenges and gaps surrounding medical student wellbeing surveys. |
| LoBasso et al. 2022 | Prospective Cohort study | The impact of a mindfulness bracelet on emotional affect in medical students: a prospective cohort study | 12 | NA | The study aimed to investigate the usefulness of a simple mindfulness reminder (in the form of a bracelet) in improving psychological symptoms and well-being in students. | All first-year medical students were invited to obtain a bracelet. There were three surveys obtained at three different timepoints (baseline, 1 month, 2 months). These surveys contained questions about frequency of use of the mindfulness bracelet as well as the PROMIS questionnaire. The PROMIS questionnaire evaluated the positive feelings and experiences which the student may have experienced. | Response rate fell from 100% to 75% to 69% from the first to third survey. There were no statistically significant benefits in positive emotions as measured by the PROMIS survey between students who wore the bracelet less and those who wore the bracelet more frequently. Though, the overall positive affect was greater in students who wore the bracelet at a higher frequency. The study also found a significant decline in positive affect among students during the initial one month of medical school. There was an improvement in the second month but did not recover to baseline. | The intervention did not yield positive results on the emotional effect of medical students. However, it does demonstrate the negative impact medical school has on first year students. |
| Loh et al. 2022 | Non-blinded Randomised Controlled  Trial | The Effects of a Brief Mindfulness Intervention on Mindfulness, Stress and Emotional Intelligence in Medical Students | 16 | NA | The study aimed to evaluate the effects of a brief mindfulness-based intervention programme in reducing stress and improving mindfulness and emotional intelligence (EI) among medical students. | The programme consisted of weekly group-based sessions which were supplemented by daily home-based exercises. The measures used to evaluate the effects of the intervention were validated scales. This includes MAAS, PSS and USMEQ-I. These were administered one-week pre intervention and one week post intervention. | Participants experienced a significant increase in mindfulness level, emotional intelligence as well as a significant decrease in perceived stress level following the intervention, despite the briefer duration of the programme. | This study provides preliminary evidence that this program is beneficial in enhancing mindfulness, perceived stress and emotional intelligence. It may be adapted into the medical curriculum to improve the wellbeing of medical students. |
| Mohmand et al. 2022 | Systematic Review | How are Medical Institutions Supporting the Well-being of Undergraduate Students? A Scoping Review | NA | NA | The study aimed to identify interventions that have been implemented to improve medical student well-being and reduce burnout, and to analyse the characteristics of these interventions. | A scoping review of the literature on medical education programs aimed at improving the wellbeing of medical students was conducted. | 1068 studies were identified with 19 meeting the inclusion criteria. They were then categorised into 4 categories: mindfulness-based programs, reflection groups, curriculum changes and “miscellaneous” The outcome assessment included stress/resilience as well as additional domains such as academic performance, mental health and interpersonal skills. There were also studies which determined the acceptability of the intervention by students. | There is a common theme that the programs are more successful when participation is voluntary. Adding mandatory content or activities can have the opposite effect. Further research with high quality intervention studies involving randomisation, blinding and rigorous controls is needed. |
